# The anti-tumor effects of the combination of microwave hyperthermia and lobaplatin against breast cancer cells *in vitro* and *in vivo*

**DOI:** 10.1042/BSR20190878

**Published:** 2022-02-09

**Authors:** Xiaohu Li, Xin Zhang, Inam Ullah Khan, Nina N. Guo, Bing Wang, Yifeng Guo, Bufan Xiao, Yueshan Zhang, Yimin Chu, Junsong Chen, Fang Guo

**Affiliations:** 1 Key Laboratory of Systems Biomedicine (Ministry of Education), Shanghai Center for Systems Biomedicine, Shanghai Jiao Tong University, 800 Dongchuan Road, Shanghai 200240, China; 2 Department of Urology, Shanghai General Hospital, Shanghai Jiao Tong University School of Medicine, No. 100 Haining Road, Hongkou, Shanghai 200080, China; 3 First Clinical Medical College, Nanchang University, Nanchang 330031, China; 4 Department of Oncology, Shanghai Putuo District Liqun Hospital, Shanghai 200333, China; 5 Digestive Endoscopy Center, Tongren Hospital, Shanghai Jiaotong University School of Medicine, Shanghai 200336, China; 6 School of Pharmacy, Shanghai Jiao Tong University, 800 Dongchuan Road, Shanghai 200240, China; 7 Shuguang Hospital Affiliated to Shanghai University of Traditional Chinese Medicine, 528 Zhangheng Road, Shanghai 201203, China

**Keywords:** apoptosis, breast cancers, lobaplatin, mechanistic target of rapamycin, microwave hyperthermia

## Abstract

**Background:** Breast cancer is the main lethal disease among females. The combination of lobaplatin and microwave hyperthermia plays a crucial role in several kinds of cancer in the clinic, but its possible mechanism in breast cancer has remained indistinct.

**Methods:** Mouse models were used to detect breast cancer progression. Cell growth was explored with MTS (3-(4,5-dimethylthiazol-2-yl)-5-(3-carboxymethoxyphenyl)-2-(4-sulphonyl)-2H-tetrazolium) and colony formation assays. Cell migration and invasion were investigated with a transwell assay. Cell apoptosis was probed with flow cytometry. The expression of apoptosis-associated proteins was examined with Western blots.

**Result:** Combination treatment decreased breast cancer cell viability, colony formation, cell invasion and metastasis. In addition, the treatment-induced breast cancer cell apoptosis and autophagy, activated the c-Jun N-terminal kinase (JNK) signaling pathway, suppressed the protein kinase B (AKT)/mammalian target of rapamycin (mTOR) signaling pathway, and down-regulated IAP and Bcl-2 family protein expression.

**Conclusion:** These results indicate that lobaplatin is an effective breast cancer anti-tumor agent. Microwave hyperthermia was a useful adjunctive treatment. Combination treatment was more efficient than any single therapy. The possible mechanism for this effect was mainly associated with activation of the JNK signaling pathway, inactivation of the AKT/mTOR signaling pathway and down-regulation of the Bcl-2 and IAP families.

## Introduction

Breast cancer is the most frequently diagnosed cancer and the main cause of cancer deaths among females worldwide. Breast cancer accounts for 15% of all cancer deaths and 25% of all cancer cases among females [1]. It is therefore necessary to develop novel therapies and anti-tumor agents. Lobaplatin is a third-generation platinum (D-19466, 1,2-diamino-methyl-cyclobutane-platinum (II)-lactate) drug [2]. It is considered a potential drug for the treatment of multiple solid tumors, with encouraging anti-cancer activity and low organ toxicity [3–5].

Hyperthermia, raising the temperature of tumor-loaded tissue to 40–43°C, has been applied as an adjunctive therapy with various established cancer treatments, such as radiotherapy and chemotherapy [6]. Combining hyperthermia with other cancer treatment modalities makes them more effective [7]. Microwaves and ultrasonic waves were utilized to treat superficial tumors, and whole-body hyperthermia was used to enhance the effects of chemotherapy [8].

Cell death may occur through many mechanisms, such as apoptosis, necrosis or autophagy [9]. Autophagy acts as a double-edged sword in breast cancer cell death. It is believed that over-stress will lead to cancer cell death through autophagy. On the other hand, many reports revealed that the inhibition of autophagy augments cancer cell sensitivity to diverse therapies [10]. Our study indicated that the combination therapy of microwave hyperthermia and lobaplatin-induced autophagy and apoptosis in breast cancer cells. Cell death induced by combination therapy played a key role in breast cancer treatment. Microwave hyperthermia usually induces tumor cell death by apoptosis and necrosis. However, mild hyperthermia therapy mainly induces breast cancer cell death through apoptosis [11].

Lobaplatin has been used in preclinical studies of several cancer treatments, such as breast cancer, head and neck tumors and pancreatic cancer [3–5,12–14]. The combination of microwave hyperthermia and radiotherapy or chemotherapy demonstrated better effects on solid tumors than monotherapy [15,16]. However, the molecular mechanism behind combination microwave hyperthermia and lobaplatin is still ambiguous.

The present study found that combination therapy was more effective than a single chemotherapy or microwave hyperthermia *in vivo* and *in vitro*. Combination treatment induced breast cancer cell (4T1 and MDA-MB-231) death through apoptosis. The present study also demonstrated the molecular mechanism behind the combination therapy of microwave hyperthermia and lobaplatin in breast cancer cells.

## Materials and methods

### Cell lines

4T1 and MDA-MB-231 breast cancer cells (ATCC, Manassas, VA) were cultured in DMEM (HyClone) containing 10% Fetal Bovine Serum (FBS) and 1% penicillin/streptomycin. Cells were maintained in a 37°C and 5% CO_2_ incubator.

### Preparation of lobaplatin

Lobaplatin (Hainan Changan International Pharmaceutical Co., Ltd.) was stored at 4°C. Lobaplatin was prepared as a sterilized stock solution (30 mg/ml) and stored at −20°C. The stock solution was diluted with DMEM or phosphate buffer saline (PBS) in experiments.

### Mice and tumor models

Female 6-week-old (20–25 g) BALB/c mice were purchased from the Shanghai SLRC Laboratory Animal Center. Mice were fed in a specific pathogen-free (SPF) animal facility. Identical viable 4T1 cells (5 × 10^5^) were administered into the mammary fat pad of female BALB/c mice. After 7 days, mice were randomized into four groups: Control, Heat, Lobaplatin, Lobaplatin + Heat. Lobaplatin was intraperitoneally injected (5 mg/kg), and an equal volume of PBS was used as control. One hour after lobaplatin injection, mice were heated in a microwave machine (Shanghai Huayuan Hyperthermia Technology Co., Ltd.) at 43°C for 1 h, and kept at a temperature that fluctuated within 0.5°C. Lobaplatin was administered once a week, while microwave hyperthermia was performed twice a week. Tumor was measured with a caliper twice a week when the mice were given microwave hyperthermia. The formula used to calculate tumor volume was π/6 × length × width/2. A month after cancer inoculation the mice were sacrificed, their tumors were stripped, and tumor metastases were counted. The experiment was approved by the committee for the Humane Treatment of Animals Shanghai Jiaotong University. The animal work took place in Shanghai Jiao Tong University Laboratory Animal Center and was compliant with all of the relevant ethical regulations regarding animal research. The Experimental Animal Ethics No. A 2017054.

#### Anesthetics

A total of 0.125 g of 2,2,2-Tribromoethanol and 0.25 ml of 2-Methyl-2-butanol were added into 10 ml sterile water to make working concentration anesthetic. Each mouse was injected with ∼300 µl (125–400 mg/kg). Mice were sacrificed by CO_2_ inhalation.

### Evaluating tumor cell colonization

BALB/c mice were randomly subdivided into four groups, with six mice per group. Identical viable 4T1 cells (5 × 10^5^) were injected into mice via tail intravenous injection. Four groups were Control, Heat, Lobaplatin, Lobaplatin + Heat. Lobaplatin was intraperitoneally injected (5 mg/kg), and an equal volume of PBS was used as control. One hour after lobaplatin injection, mice were heated in a microwave machine (Shanghai Huayuan Hyperthermia Technology Co, Ltd) at 43°C for 1 h, and kept at a temperature that fluctuated within 0.5°C. Lobaplatin was administered once a week, while microwave hyperthermia was performed twice a week. On the 16^th^ day the mice were sacrificed, and their lungs were subjected to postmortem. Colonized nodule diameters were evaluated under the dissecting microscope. The total number of lung surface colonizations were recorded. Lung colonized nodules with a diameter of more than 1 mm were counted. The experiment was approved by the committee for the Humane Treatment of Animals Shanghai Jiaotong University. The animal work took place in Shanghai Jiao Tong University Laboratory Animal Center and was compliant with all of the relevant ethical regulations regarding animal research. The Experimental Animal Ethics No. A 2017054.

#### Anesthetics

A total of 0.125 g of 2,2,2-Tribromoethanol and 0.25 ml of 2-Methyl-2-butanol were added into 10 ml sterile water to make working concentration anesthetic. Each mouse was injected with ∼300 µl (125–400 mg/kg). Mice were sacrificed by CO_2_ inhalation.

### Cell viability assays

The 4T1 and MDA-MB-231 breast cancer cells were treated with lobaplatin or lobaplatin combined with hyperthermia. Cell proliferation was measured with the MTS (3-(4,5-dimethylthiazol-2-yl)-5-(3-carboxymethoxyphenyl)-2-(4-sulphonyl)-2H-tetrazolium) assay (Promega). 4T1 and MDA-MB-231 cells were seeded in 96-well plates with 3000 and 5000 cells per well, respectively. Treatments consisted of different concentrations of lobaplatin 0, 5, 10, 20, 40 (μg/ml), and heated at 43°C for 1 h. After 24, 48 and 72 h of treatment, cell proliferation was measured with the MTS method. Absorbance was measured with a Thermo Scientific Varioskan flash (Thermo Fisher Scientific) at 490 nm.

### Flow cytometry

Cells were seeded in six-well plates and treated with lobaplatin 10 μg/ml and microwave hyperthermia at 43°C for 1 h. After 24 h, the cells were incubated with Propidium Iodide (PI) and Annexin V for 15 min at room temperature in a dark place. The cells were then suspended and examined with flow cytometry. Apoptotic cell quantification was performed according to the Annexin V-FITC Apoptosis Detection Kit (Thermo Fisher Scientific) protocol.

### Migration and invasion assay

Breast cancer 4T1 and MDA-MB-231 cells were treated with lobaplatin 10 μg/ml, microwave hyperthermia at 43°C for 1 h and combination treatment. After being washed with PBS, cells (5 × 10^5^/100 μl DMEM) were seeded in the upper well (8-μm polycarbonate membrane, Falcon) of a 24-well plate and 500 μl of 20% FBS DMEM was added to bottom well. After incubation for 24 h, the membranes with the migrated cells were stained with 0.1% Crystal Violet. Migrated cells were photographed, counted and statistically analyzed. For the invasion assay, the upper wells with the polycarbonate membrane were coated with 60 μl of Matrigel (3 mg/ml, Becton, Dickinson and Company). Cells were seeded, treated and statistically analyzed according to the transwell migration assay protocol.

### Antibodies and Western blot analysis

Phosphorylated extracellular-regulated protein kinases (P-ERKs), LC3B (MBL), p62 (Novus), protein kinase B (also called AKT), P-AKT (Ser^473^), P-AKT (Thr^308^), poly-ADP ribose polymerase (PARP), Caspase3, Bad, Bid, Bim, Bax, Bak, Bcl-2, Bcl-xl, myeloid cell leukemia-1 (Mcl-1), cellular inhibitor of apoptosis protein 1 (c-IAP1), c-IAP2, X-linked inhibitor of apoptosis protein (XIAP), mammalian target of rapamycin (mTOR), P-mTOR (Ser^2448^), P-mTOR (Ser^2481^), adenosine 5′-monophosphate (AMP)-activated protein kinase (AMPK), CypA, HMGB1, P-AMPK, c-Jun N-terminal kinase (JNK), P-JNK, P-P38, p70 ribosomal protein S6 kinases (P70S6Ks) and P-P70S6K were purchased from Cell Signaling Technology Co. After being washed with ice-cold PBS three times, cells were lysed with RIPA lysis buffer (1% Triton X-100, 0.1% SDS, 50 mM Tris pH 7.4, 150 mM NaCl, 0.5% sodium deoxycholate) and supplemented with 10 mM NaF, 2 mM PMSF, 2 mM Na_3_VO_4_ and protease inhibitors (Roche). Equal amounts of protein (30 μg) was loaded on to 12% SDS/PAGE, and transferred to polyvinylidene fluoride (PVDF) membranes (Millipore).

### Hematoxylin and Eosin staining

Lungs were fixed with 4% paraformaldehyde (PFA), dehydrated, paraffin embedded and sectioned (4 μm). Lung sections were stained with Hematoxylin and Eosin (H&E).

### Statistical analysis

All statistical calculations were performed using analysis of variance (ANOVA). A statistical significance value of *P*<0.05 was regarded as statistically significant. Data are expressed as the mean ± standard deviation (SD).

## Results

### The effects of lobaplatin and microwave hyperthermia on tumor cell colonization and tumor invasion

**Figure 1:**
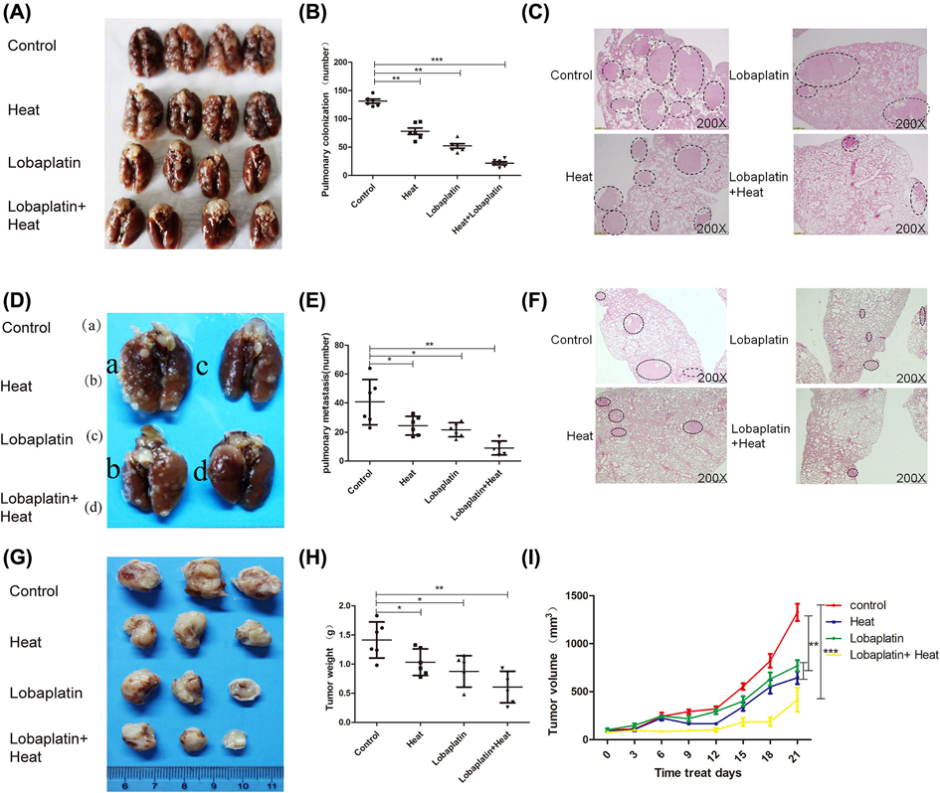
The inhibitory effects of the combination therapy of microwave hyperthermia and lobaplatin on tumor colonization, growth and metastasis *in vivo* The lung metastasis of 4T1 cells was inhibited by the combination therapy of microwave hyperthermia and lobaplatin in an pulmonary colonization model of BALB/c mice (**A**,**B**). Lungs were stained with H&E (**D**–**I**). The effects of the combination therapy of microwave hyperthermia and lobaplatin on tumor growth and metastasis in an orthotopic transplantation tumor model. Combination treatment of microwave hyperthermia and lobaplatin significantly inhibited 4T1 mammary cancer cell distant metastasis compared with a single therapy (D–F). (F) Lungs were stained with H&E. (A) Control: PBS was administered via intraperitoneal injection. (B) Heat: microwave hyperthermia was administered. (C) Lobaplatin: Lobaplatin was administered by intraperitoneal injection. (D) Lobaplatin + Heat: Lobaplatin and heat were administered by intraperitoneal injection and microwave hyperthermia, respectively. Combination therapy significantly suppressed the tumor volume and decreased the tumor weight compared with those of the vehicle-treated control mice (G–I). Magnification: ×200 (*n*=6 per group, *P<0.05, **P<0.01, ***P<0.001 based on Student’s *t* test). Data are presented as the mean ± SD.

The pulmonary colonization model was set up in BALB/c mice (Figure 1A–C). Pulmonary colonized nodules (diameter ≥ 1 mm) were counted. The greatest incidence of pulmonary colonized nodules was observed in the control group. Lobaplatin or microwave hyperthermia therapy effectively reduced the number of pulmonary colonized nodules. Furthermore, the combination of lobaplatin and microwave hyperthermia therapy significantly reduced the number of pulmonary colonized nodules (diameter ≥ 1 mm) compared with that after control treatment, lobaplatin treatment or microwave hyperthermia therapy alone. Pulmonary colonized nodules were verified with H&E staining (Figure 1A).

An orthotopic transplant tumor model was used to study the inhibitory effects of combination therapy on tumor invasion and metastasis (Figure 1D–I). Lobaplatin or microwave hyperthermia monotherapy suppressed tumor volume and growth. Furthermore, combination therapy significantly suppressed the tumor volume and decreased the tumor weight compared with those of the vehicle-treated control mice (Figure 1G–I). In addition, the results showed that the combination treatment of microwave hyperthermia and lobaplatin significantly inhibited 4T1 mammary cancer cell distant metastasis compared with a single therapy (Figure 1D–F). These results showed that the combination treatment of microwave hyperthermia and lobaplatin significantly suppressed breast cancer progression *in vivo*. The microwave therapy device schematic diagram was shown in Figure supplementary 5.

### The inhibitory effects of the combination therapy of microwave hyperthermia and lobaplatin on breast cancer cell invasion and migration *in vitro*

**Figure 2:**
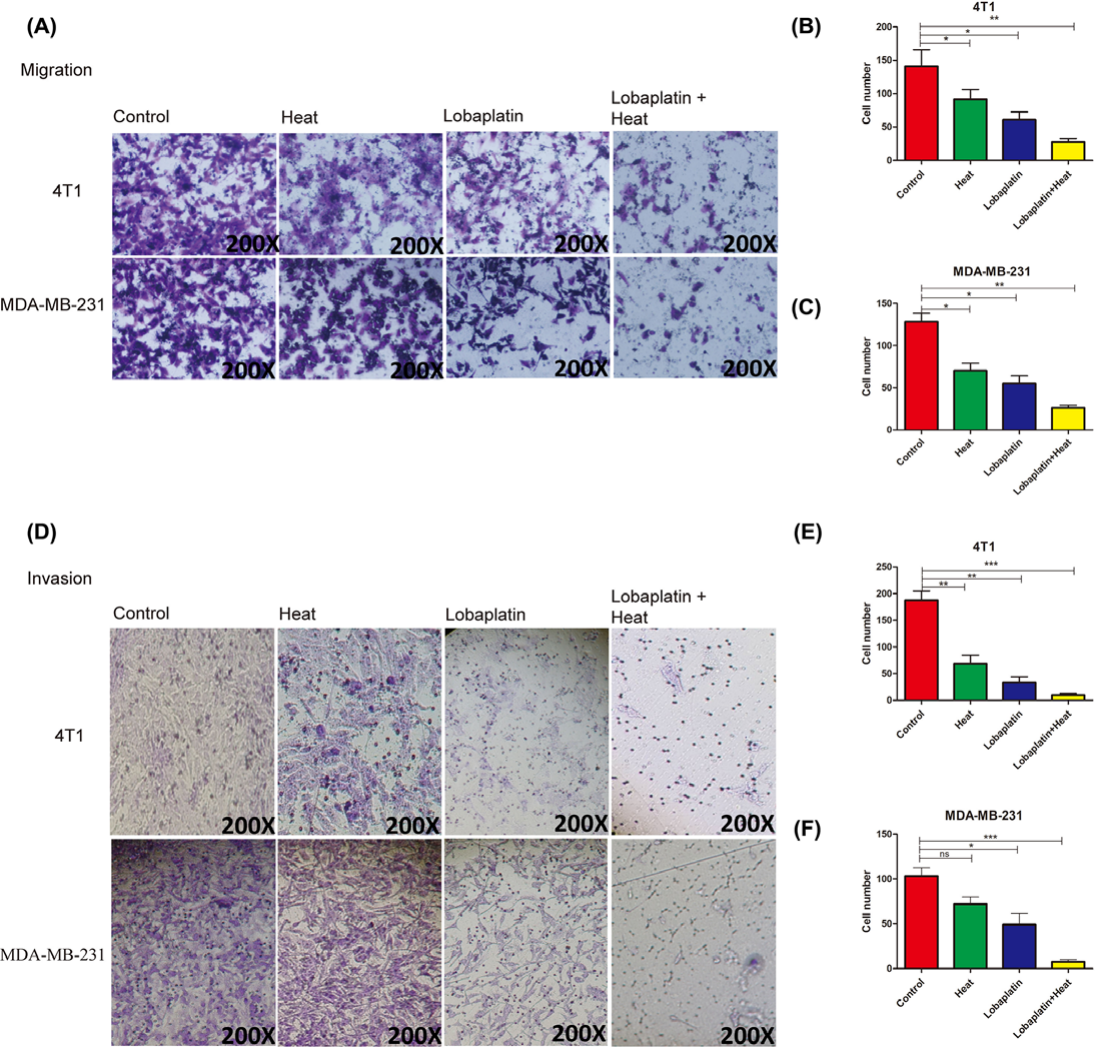
Combination treatment suppressed the migration and invasion of breast cancer 4T1 and MDA-MB-231 cells Cells were treated with lobaplatin, hyperthermia and combination therapy (A). After treatment, cells were incubated in 5% CO_2_ at 37°C for 24 h. Cells were seeded in transwell without Matrigel. Twenty-four hours later, cells were photographed and counted (**A–C**). Cell invasion of 4T1 and MDA-MB-231 cells. Cells were treated as in (A), and in transwell with Matrigel (**D**). Twenty-four hours later, cells were photographed and counted in (**E**,**F**). (*P<0.05, **P<0.01, ***P<0.001 based on Student’s *t* test). Data are presented as the mean ± SD. Means and standard deviations in the bar charts are from three independent experiments.

A transwell assay was used to investigate breast cancer cell (4T1 and MDA-MB-231) migration and invasion. 4T1 cells were treated with lobaplatin, microwave hyperthermia and combination treatment (Figure 2A). Migrated cells were counted in each image as shown in Figure 2A–C. The combination treatment group had the lowest migration rate, while lobaplatin and hyperthermia had a secondary inhibited migration rate of 4T1 and MDA-MB-231 cells (Figure 2A–C). Invasion was analyzed using a transwell assay with Matrigel. Breast cancer cell behavior was tested. Breast cancer cells were treated as in Figure 2A,D. Transwell with Matrigel, and cells under transwell membrane were counted (Figure 2E,F). The invasion rate of 4T1 and MDA-MB-231 cells were lower than that of single treatments and control. These results indicated that combination markedly inhibited breast cancer cells migration and invasion *in vitro*.

### The inhibitory effects of the combination of microwave hyperthermia and lobaplatin on breast cancer cell proliferation

**Figure 3:**
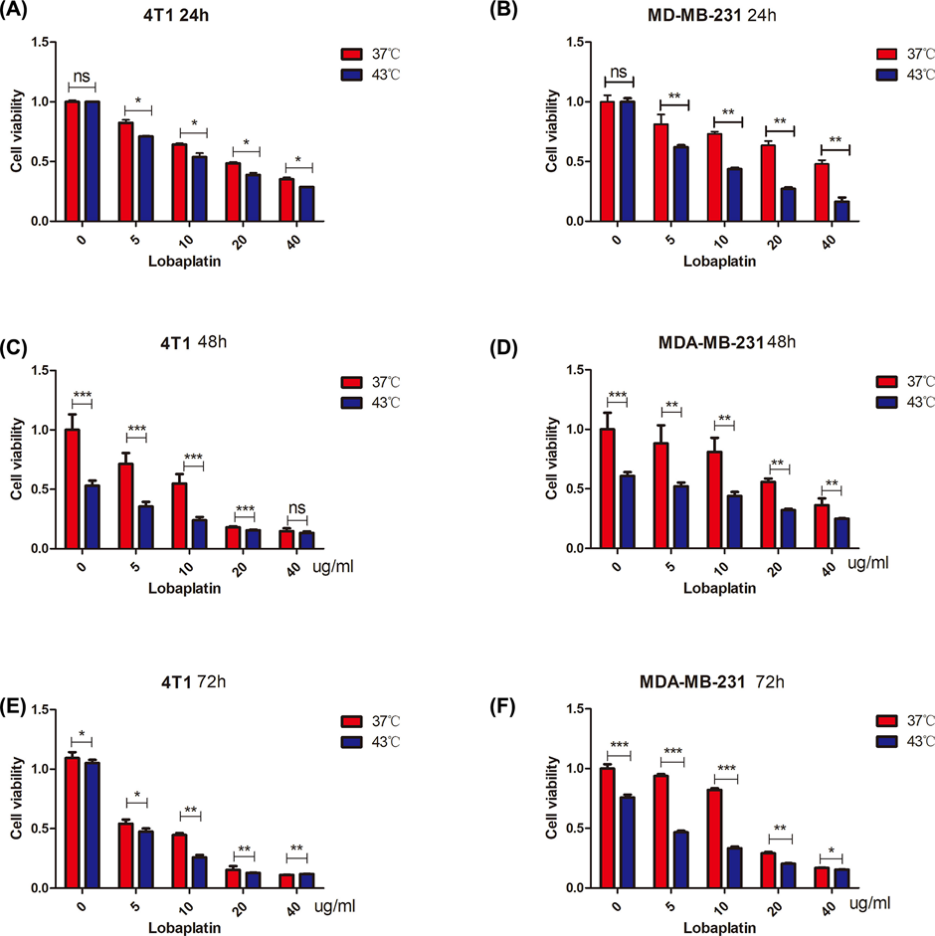
Combination treatment suppressed the proliferation and colony ability of 4T1 and MDA-MB-231 cells 4T1 and MDA-MB-231 cells were seeded at 3000 and 5000 per well on 96-well plates. Cells were treated with lobaplatin and microwave hyperthermia at different concentrations (0, 5, 10, 20, 40 µg/ml) and temperatures (37 and 43°C) for 24, 48 and 72 h. Cell viability was detected with the MTS assay (**A–F**). The combination of microwave hyperthermia and lobaplatin suppressed breast cancer cell viability *in vitro* (*n*=6, *P<0.05, **P<0.01, ***P<0.001 based on Student’s *t* test). Data are presented as the mean ± SD. Means and standard deviations in the bar charts are from three independent experiments.

4T1 and MDA-MB-231 cells were treated with different concentrations of lobaplatin for 24, 48 and 72 h (Figure 3A–F). Combination therapy of lobaplatin and microwave hyperthermia effectively inhibited cell growth compared with controls and lobaplatin or microwave hyperthermia treatment alone in 4T1 and MDA-MB-231 cells. Further colony-forming assays were utilized to test the colony-forming ability of 4T1 and MDA-MB-231 cells (Supplementary Figure S1A). The combination therapy of microwave hyperthermia and lobaplatin had the highest cytotoxicity among the four groups of breast cancer cells.

### The combination of hyperthermia and lobaplatin induced both apoptosis and autophagy in 4T1 and MDA-MB-231 cells

**Figure 4:**
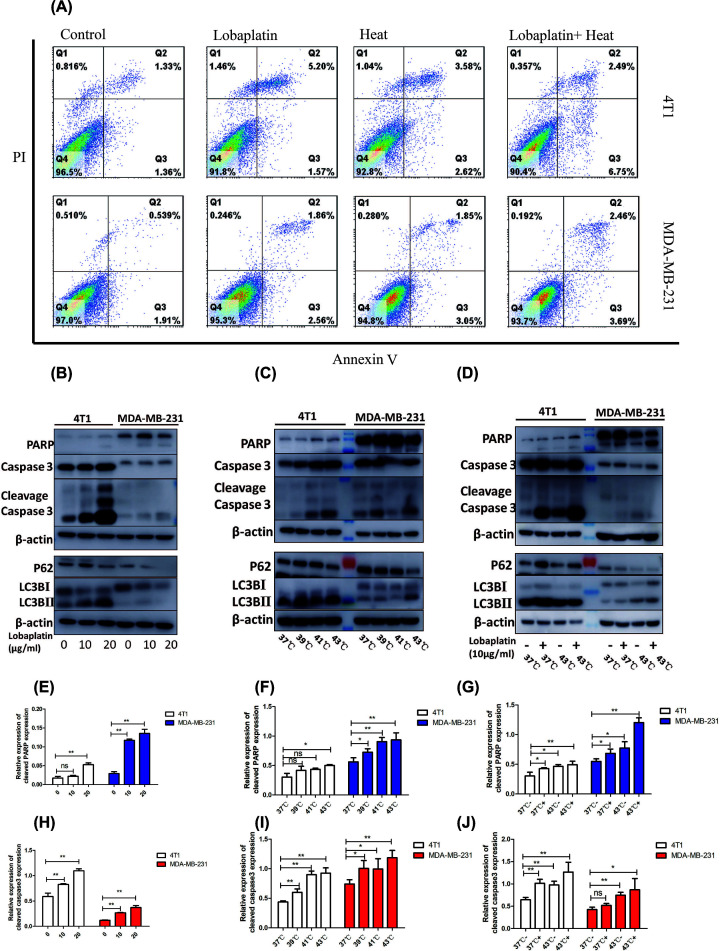
The combination of lobaplatin and microwave hyperthermia induces the apoptosis and autophagy in breast cancer cells (**A**) FCM assay for apoptosis: 4T1 and MDA-MB-231 cells were treated in either the absence or presence of lobaplatin (10 µg/ml) or an indicated temperature for 1 h. (**B**) 4T1 and MDA-MB-231 cells were treated with 0, 10, 20 µg/ml lobaplatin for 24 h. (**C**) 4T1 and MDA-MB 231 cells were treated with microwave hyperthermia at 37, 39, 41 and 43°C for 1 h, respectively. (**D**) 4T1 and MDA-MB 231 cells were treated in either the absence or presence of lobaplatin (10 µg/ml) or at an indicated temperature. (**E**,**H**) Relative expression of cleaved PARP and caspase 3 expression of 4T1 and MDA-MB-231 cells were treated with 0, 10, 20 µg/ml lobaplatin for 24 h. (**F**,**I**), Relative expression of cleaved PARP and caspase3 expression of 4T1 and MDA-MB 231 cells were treated with microwave hyperthermia at 37, 39, 41 and 43°C for 1 h. (**G**,**J**) Relative expression of cleaved PARP and caspase 3 expression of 4T1 and MDA-MB 231 cells were treated in either the absence or presence of lobaplatin (10 µg/ml) or at an indicated temperature. Twenty-four hours later, PARP Caspase 3, LC3B and P62 were detected on Western blot analysis. β-actin was used to confirm that the proteins equal in each lane. Means and standard deviations in the bar charts were from three independent experiments. ns means not significant, *P<0.05, **P<0.01, based on Student’s test. Data are presented as the mean ± SD.

Flow cytometry analysis of FITC-Annexin V/PI was used to investigate cell viability and cell death. As shown in Figure 4A, microwave hyperthermia or lobaplatin increased cell apoptosis. We hypothesized that combination therapy induced breast cancer cell apoptosis, which plays a key role in suppressing breast cancer progression. Western blots were utilized to detect the hallmarks of apoptosis [17]. The data showed that both microwave hyperthermia and lobaplatin induced PARP and Caspase 3 cleavage. Our data showed that combination treatment significantly induced more 4T1 and MDA-MB-231 cell apoptosis than that of both therapies alone (Figure 4B–J). Furthermore, the autophagy hallmarks LC3B, P62 were detected, with increased LC3BII and decreased P62 (Figure 4B–D). These results show that combination treatment induced both the apoptosis and autophagy of breast cancer cells.

Transwell assay was utilized to investigate the *in vitro* autophagy functions of breast cancer cells in migration and invasion. BFA (autophagy inhibitor) was utilized to inhibit autophagy flux. As shown in Supplementary Figure S2A,B, both migration and invasion were significantly decreased in 4T1 and MDA-MB-231 cells. Our research shows that the migration and invasion ability of 4T1 and MDA-MB-231 cells were suppressed *in vitro* when autophagy was inhibited. However, our data show that BFA (10 nmol/l) has an effect on invasion, but has no effect on migration (Supplementary Figure S2C,D). The optimal BFA concentration needs further exploration.

### Combination therapy with hyperthermia and lobaplatin inhibited the PI3K/AKT/mTOR signaling pathway and activated the JNK signaling pathway in 4T1 and MDA-MB-231 cells

**Figure 5:**
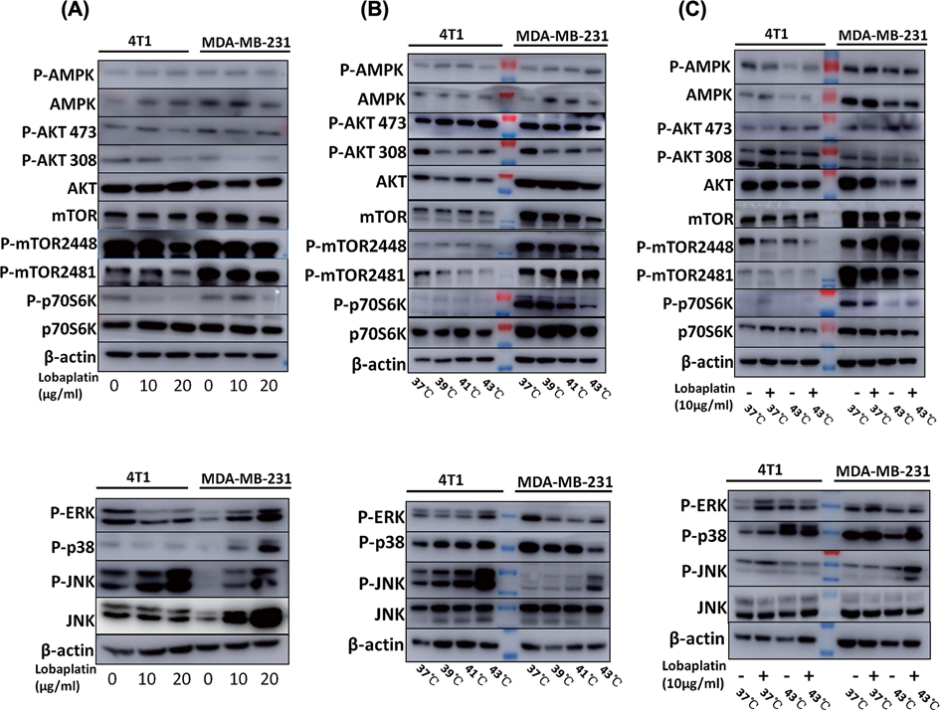
The PI3K/AKT/mTOR pathway was suppressed and the JNK signaling pathway of 4T1 and MDA-MB-231 cells was activated by lobaplatin, microwave hyperthermia and combination therapy (**A**) 4T1 and MDA-MB-231 cells were treated with 0, 10 and 20 µg/ml lobaplatin. (**B**) 4T1 and MDA-MB-231 cells were treated with microwave hyperthermia at 37, 39, 41 and 43°C for 1 h. (**C**) 4T1 and MDA-MB-231 cells were treated in either the absence or presence of lobaplatin (10 μg/ml) or at an indicated temperature. Twenty-four hours later, P-mTOR, mTOR, P-AMPK, AMPK, P-P70S6K, P-AKT, AKT, P-ERK, P-P38, P-JNK and JNK were detected by Western blot analysis. β-actin was used to confirm that the proteins equal in each lane. Means and standard deviations in the bar charts were from three independent experiments.

As shown in Figure 5A–C, lobaplatin, microwave hyperthermia and combination treatment affected the PI3K/AKT/mTOR pathway. The combination of lobaplatin and microwave hyperthermia reduced the phosphorylation of mTOR, AKT, and P70S6K. Consistent with Figures 1-3, combination treatment reduced the metastasis of breast cancer *in vitro* and *in vivo*. The data indicated that lobaplatin, microwave hyperthermia and combination treatment induced breast cancer cell apoptosis via suppression of the PI3K/AKT/mTOR signaling pathway and activation of the JNK signaling pathway.

### The combination therapy of hyperthermia and lobaplatin suppressed the anti-apoptotic Bcl-2 and IAP proteins in 4T1 and MDA-MB-231 cells

**Figure 6:**
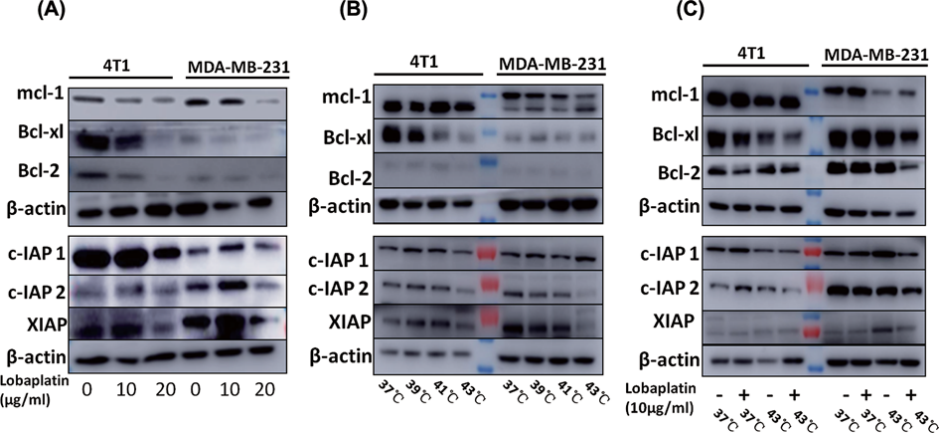
The combination therapy of microwave hyperthermia and lobaplatin synergistically regulates Bcl-2 family and IAP family proteins in breast cancer cells 4T1 and MDA-MB-231 were treated with microwave hyperthermia, lobaplatin and combination treatment as in Figure 4. (**A**) 4T1 and MDA-MB-231 cells were treated with lobaplatin. (**B**) 4T1 and MDA-MB-231 cells were treated with microwave hyperthermia at 37, 39, 41 and 43°C for 1 h. (**C**) 4T1 and MDA-MB-231 cells were treated with the absence or presence of lobaplatin (10 µg/ml) or the indicated temperature. Twenty-four hours later, Bcl-2, Bcl-xl, Mcl-1, c-IAP1, c-IAP2 and XIAP were detected by Western blot analysis. β-actin was used to confirm that the proteins were equal in each lane.

Western blots were used to investigate Bcl-2, Bcl-xl, Mcl-1, c-IAP1, c-IAP2 and XIAP protein expression (Figure 6). Our data showed that the combination of microwave hyperthermia and lobaplatin synergistically suppressed the anti-apoptotic Bcl-2 and IAP family of proteins in breast cancer cells. Western blots were used to investigate the pro-apoptotic effect of proteins (Supplementary Figure S4). These data indicated that microwave hyperthermia enhances the effects of lobaplatin on inducing cell death through the mitochondrial apoptosis pathway in breast cancer cells. We further investigate intracellular signaling pathways as control data, such as CyPA and HMGB1 in Supplementary Figure S6. Our data indicated that microwave hyperthermia and lobaplatin have no obvious effect on expression of HMGB1 and CyPA.

## Discussion

As far as we know, the molecular mechanism by which lobaplatin kills breast cancer cells has not been clearly illustrated. In the present study, we investigated a combination therapy consisting of lobaplatin and microwave hyperthermia *in vivo* and *in vitro*. MDA-MB-231 and 4T1 were selected, for further investigation since they are highly invasive TNBC breast cancer cell lines [18,19]. The 4T1 tumor is highly tumorigenic; unlike most tumor models, it can spontaneously metastasize from the primary tumor in the mammary gland to multiple distant sites [20]. We proved the combination therapy of microwave hyperthermia and lobaplatin led to synergistic anti-tumor effects *in vivo*. Actually, lobablatin has been confirmed as an effective anti-tumor drug on Retinoblastoma and against Ishikawa endometrial cancer cells [21,22].

We provided evidence that the combination of hyperthermia and lobaplatin strongly suppressed breast cancer cell metastases *in vivo* (Figure 1B). Cancer metastasis requires the invasion of tumor cells into the stroma and then migration of tumor cells through the vasculature and lymphatics to secondary organs [23].

Then, we focused on migration and invasion. Lobaplatin has been proven to inhibit the migration, invasion and proliferation of prostate cancer cell lines [24]. In this study, we confirmed the synergistic effects of combining microwave hyperthermia and lobaplatin against breast cancer in both colony-forming and transwell experiments *in vitro*. Combination therapy inhibited the breast cancer cell colony-forming ability and decreased cancer invasion and migration which is better than lobaplatin monotherapy did. In 4T1 and MDA-MB-231 more cells invade than migrate (Supplementary Figure S3E,F) under combination treatment. Our data suggest that the invasion is more sensitive to treatment than migration. MTS data indicate that lobaplatin suppressed 4T1 and MDA-MB-231 proliferation depending on the timing and dosage treatment that are in-line with previous research [25]. 4T1 is more sensitive than MDA-MB-231 to lobaplatin in cell proliferation. This mainly indicated that different cells had their most optimal doses. We confirmed that the combination of microwave hyperthermia and lobaplatin better inhibited the proliferation of breast cancer cells compared with a single therapy at 24^th^-, 48^th^- and 72^nd^-hour post treatments (Figure 3). These data are in line with the previous experiments* in vivo*.

In the present study, the combination therapy consisting of microwave hyperthermia and lobaplatin was found to induce both the apoptosis and autophagy of 4T1 and MDA-MB-231 cells. We demonstrated that lobaplatin and microwave hyperthermia could induce breast cancer cell apoptosis, especially when used in synergistic treatment. Many pathways regulate both apoptosis and autophagy. It has been well accepted that the PI3K/AKT/mTOR signaling pathway plays a major role in apoptosis and autophagy [26–28].

Our data showed that lobaplatin, microwave hyperthermia and combination treatment inhibited the PI3K/AKT/mTOR signaling pathway. Combination treatment profoundly reduced phosphorylation of mTOR, AKT and P70S6K. MAPK family contains ERK, P38 and JNK groups, which play a key role in controlling the balance between apoptosis and autophagy [29,30]. We found that lobaplatin and microwave hyperthermia synergistically promoted the phosphorylation of JNK and P38 (Figure 5).

It has been well documented that anti-apoptotic Bcl-2 and IAP family of proteins were indicators of apoptosis [31,32]. The Bcl-2 family contains the pro-apoptotic effector proteins Bak, Bax, Bid, Bad etc [33]. Bcl-xl and Mcl-1 at the mitochondrial outer membrane block apoptosis factors and control programmed cell death [34]. XIAP, c-IAP1 or c-IAP2 could directly inhibit caspase and impede apoptosis [35]. We further explored the anti-apoptotic Bcl-2 and IAP family of proteins in the setting of lobaplatin, microwave and combination therapy in 4T1 and MDA-MB-231 cells. In our study, lobaplatin, hyperthermia and combination treatment decreased the expression of anti-apoptotic proteins and significantly increased the levels of the pro-apoptotic proteins BAK and BAX, which play a key role in the intrinsic pathway of apoptosis [36].

**Figure 7:**
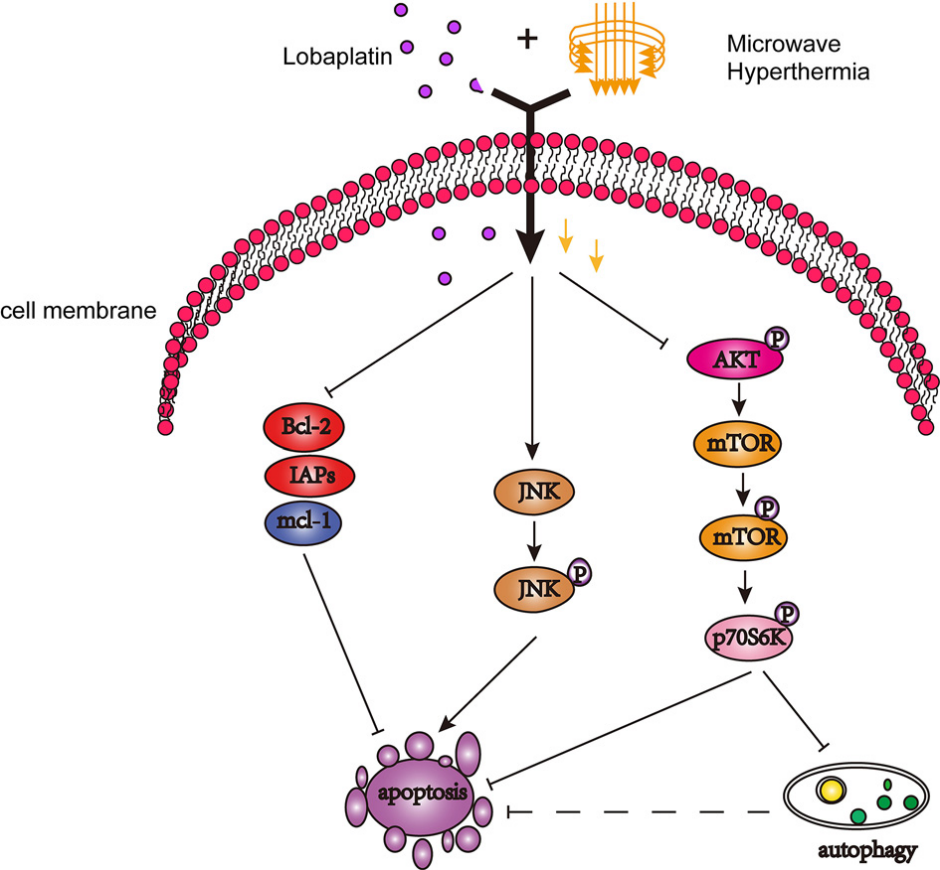
Model summarizing the autophagy and apoptosis of breast cancer cells induced by combination treatment with lobaplatin and microwave hyperthermia Combination treatment of lobaplatin and microwave hyperthermia induced the apoptosis and autophagy of 4T1 and MDA-MB-231 cells. Combination treatment activated the JNK signaling pathway, inhibited the AKT/mTOR signaling pathway and down-regulated the Bcl-2 and IAP family.

Currently, the exact mechanism of combination therapy of anti-tumor efficacy needs further exploration. However, the possible mechanisms may be the following according to our finding. In the first place, combination therapy induced 4T1 and MDA-MB-231 breast cancer cell apoptosis via the activation of the JNK signaling pathway, inhibiting the AKT/mTOR signaling pathway and down-regulating the Bcl-2 and IAP family (Figure 7). It has been reported that lobaplatin inhibits PI3K/AKT/mTOR pathway, Bcl-2 and IAP family and activates members of MAPK family so does hyperthermia [24,25,29,37–40]. Secondly, we suspected that treatments inhibiting proliferation, migration and invasion of 4T1 and MDA-MB-231 cell lines by suppressing the PI3K/AKT/mTOR pathway. This hypothesis coincides with other reports [38,41].

We further investigated other intracellular signaling pathways as control data, such as CyPA and HMGB1 in Supplementary Figure S6. Microwave hyperthermia and lobaplatin do not affect the expression of HMGB1 and CyPA. This may indicate microwave hyperthermia and lobaplatin did not have excellent synergistic effect in regulating the HMGB1-related necroptosis pathway and CyPA-related cell death pathway.

Finally, we tested the effect of combination therapy on cell pyrolysis in Supplementary Figure S7. Our data suggest that combination therapy can significantly increase the GSDMD-N cleavage (especially in 4T1 cells), indicating that combination therapy can cause cell pyroptosis significantly. Our study now, for the first time, found this phenomenon. It is still unclear whether pyroptosis was caused by microwaves or just 43°C temperature. Compared with the results of other cell pyroptosis studies, we have too many miscellaneous bands. The death way of cancer cells caused by combination therapy needs further exploration. In summary, our data revealed that lobaplatin and microwave hyperthermia synergistically inhibited tumor growth and metastasis in murine models. Combination treatments inhibit the proliferation, invasion and migration of 4T1 and MDA-MB-231 breast cancer cells by down-regulating AKT/mTOR signaling pathway. These treatments induced apoptosis and autophagy in 4T1 and MDA-MB-231 cells via activating the JNK signaling pathway, inhibiting the AKT/mTOR signaling pathway and down-regulating the Bcl-2 and IAP family. In this article, we also focus on autophagy (Supplementary Figures S2 and S3). However, the ideal dose of BFA is not clear. Further study is needed to explore the role of autophagy in breast cancer cell death and metastasis during combination treatment consisting of microwave hyperthermia and lobaplatin.

## Compliance with Ethical Standards

All experiments in this manuscript comply with the current laws of China.

## Supplementary Material

Supplementary Figures S1-S7

Supplementary Files

## Data Availability

All supporting data are included within the main article and its supplementary files.

## Abbreviations

AKT: protein kinase B
c-IAP1: cellular inhibitor of apoptosis protein 1
FBS: fetal bovine serum
H&E: Hematoxylin and Eosin
JNK: c-Jun N-terminal kinase
Mcl-1: myeloid cell leukemia-1
mTOR: mammalian target of rapamycin
MTS: 3-(4,5-dimethylthiazol-2-yl)-5-(3-carboxymethoxyphenyl)-2-(4-sulphonyl)-2H-tetrazolium
PARP: poly-ADP ribose polymerase
PBS: phosphate buffer saline
PI: propidium iodide
P70S6K: p70 ribosomal protein S6 kinase
XIAP: X-linked inhibitor of apoptosis protein
