# Supplementary material for: The anti-tumor effects of the combination of microwave hyperthermia and lobaplatin against breast cancer cells in vitro and in vivo
Source: Biosci Rep. 2022 Feb 9;42(2):BSR20190878. doi: 10.1042/BSR20190878 (PMC8829017; doi:10.1042/BSR20190878)
Supplement: Supplementary Figures S1-S7 [file BSR-2019-0878_supp.pdf]

Supplementary

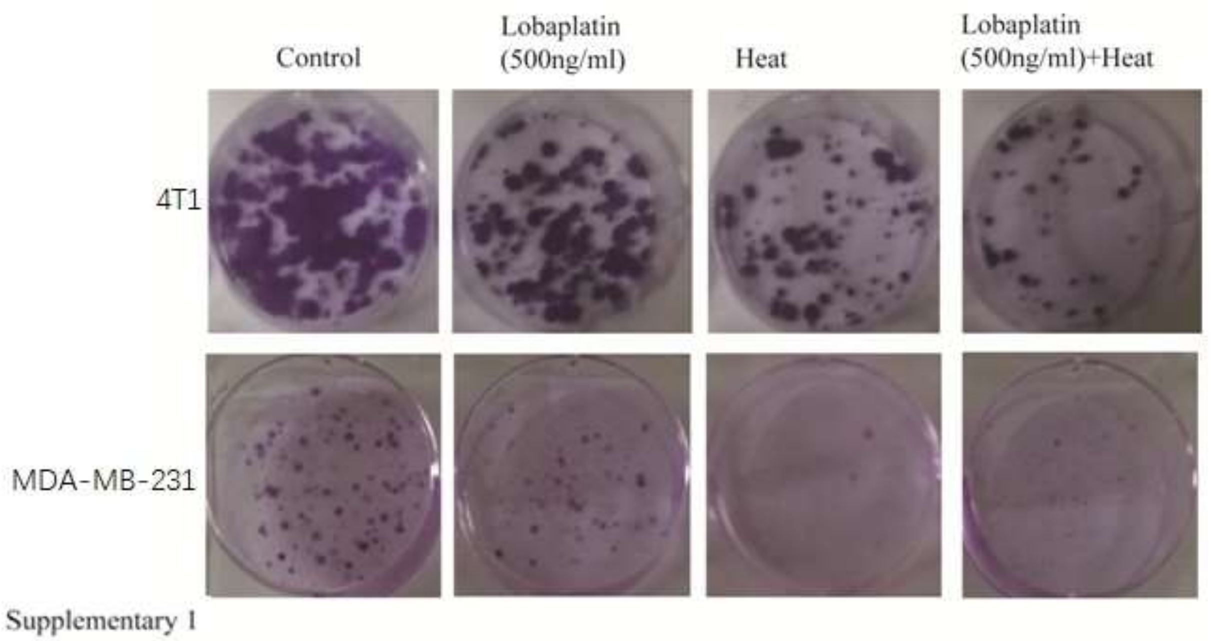

Supplementary Fig. 1 The combination treatment of lobaplatin and microwave hyperthermia decreases the colony-forming ability of breast cancer cells.

The combination therapy of microwave hyperthermia and lobaplatin decreased the colony-forming ability of breast cancer cells. MDA-MB-231 and 4T1 cells were seeded in six-well plates with lobaplatin, microwave hyperthermia and combination treatment.

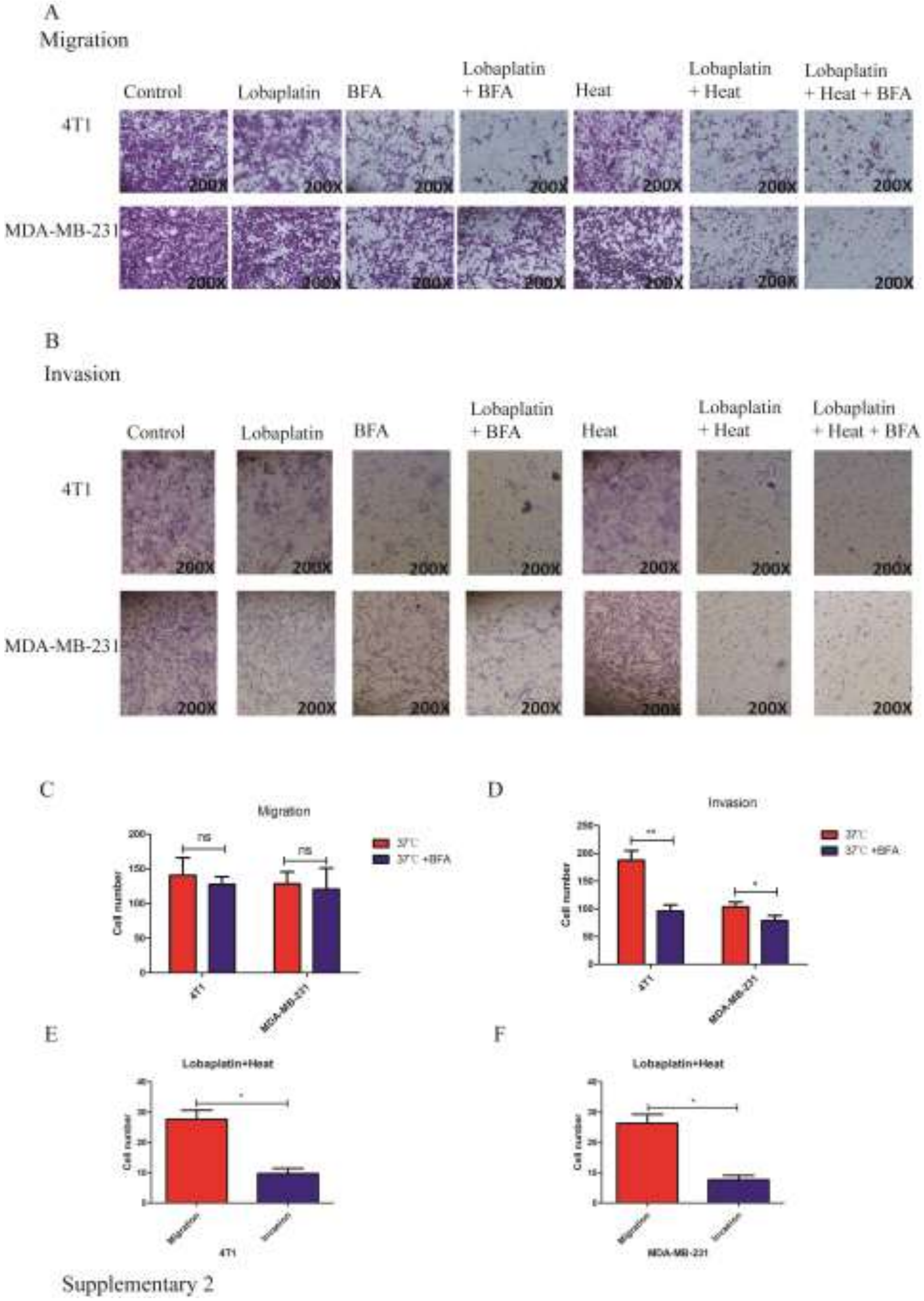

Supplementary Fig. 2 The function of the autophagy induced by combination treatment on the migration and invasion of breast cancer cells.

To determine the function of autophagy on breast cancer cell migration and invasion we used Bafilomycin A1 (Sigma-Aldrich), an autophagy inhibitor that impairs lysosomal pH and blocks the fusion of autophagosomes with lysosomes (10 nmol/L). 4T1 and MDA-MB-231 cells were seeded and treated as in Fig. 4. The migration and invasion of breast cancer cells were stained and photographed under a light microscope (A, B). Cell migration and invasion of 4T1 and MDA-MB-231 cells (37℃, 37℃ with Bafilomycin A1) were counted (C, D). Cell migration and invasion of 4T1 and MDA-MB-231 cells were counted (E, F). (*$p<0.05$, **$p<0.01$, ***$p<0.001$ based on Student's t-test). Data are presented as the mean ± SD. Means and standard deviations in the bar charts are from three independent experiments.

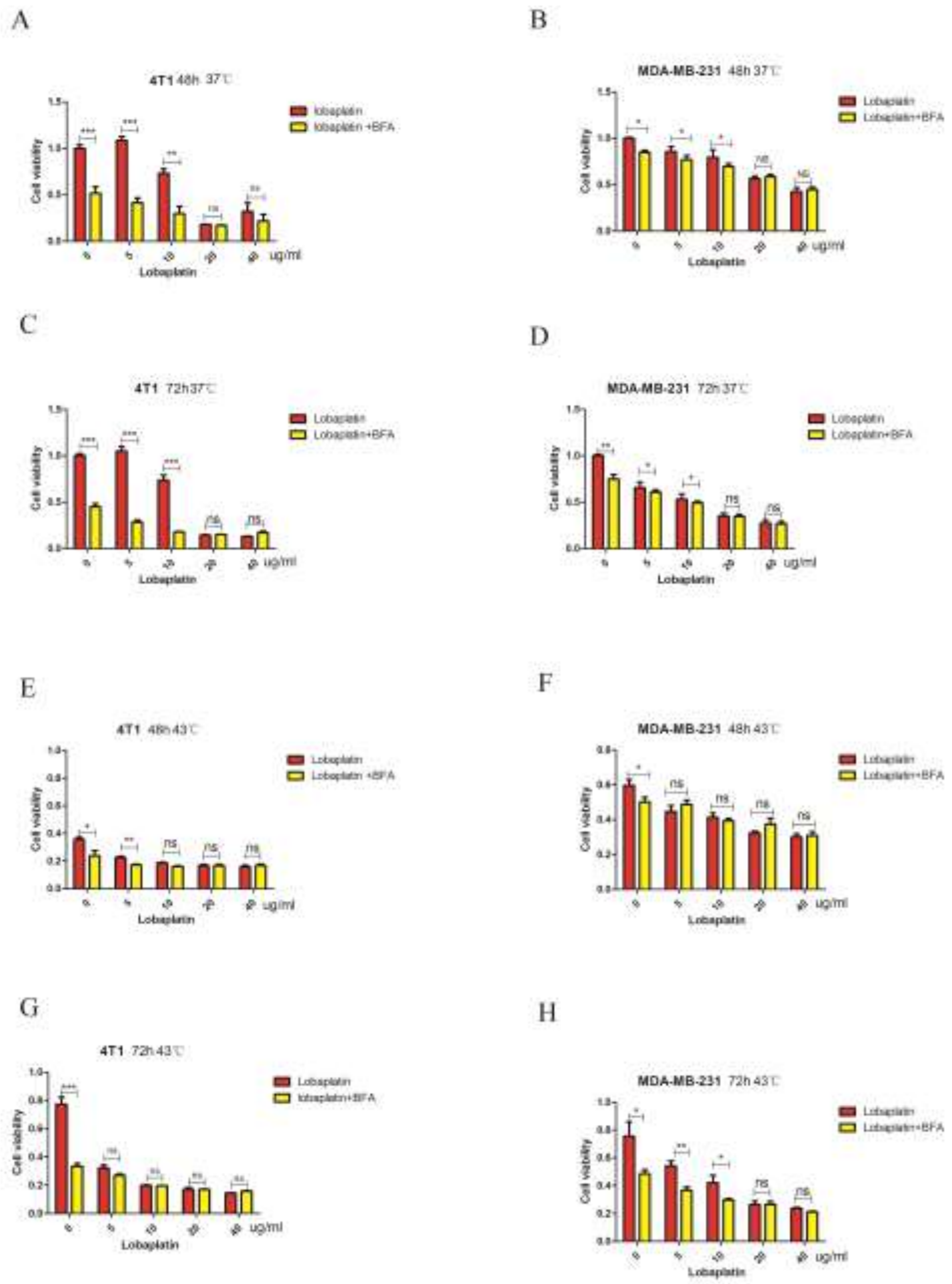

Supplementary 3

Supplementary Fig. 3 The function of the autophagy that is induced by combination treatment on the viability of breast cancer cells.

4T1 and MDA-MB-231 cells were seeded at 3000 and 5000 per well on 96 well plates. Cells were treated with lobaplatin and microwave hyperthermia at different concentrations (0, 5, 10, 20, 40 μg/ml), temperatures (37℃, 43℃) and Bafilomycin A1(10 nmol/L) for 24h,48 h and 72 h. Cell viability was detected with the MTS assay (A, B, C, D, E, F, G, H). The combination of microwave hyperthermia and lobaplatin suppressed breast cancer cell viability in vitro. (n=6, *$p<0.05$, **$p<0.01$, ***$p<0.001$ based on Student's t-test.). Data are presented as the mean ±SD. Means and standard deviations in the bar charts are from three independent experiments.

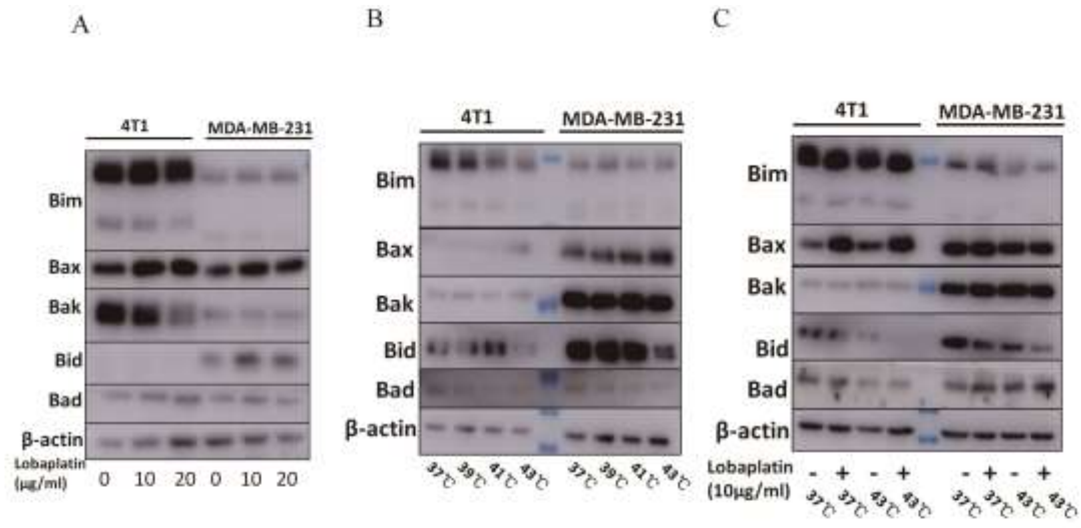

Supplementary 4

Supplementary Fig. 4 The combination therapy of hyperthermia and lobaplatin activates the pro-apoptotic Bcl-2 family of proteins in 4T1 and MDA-MB-231 cells.

4T1 and MDA-MB-231 cells were treated as in Fig. 4, Western blots were used to detect pro-apoptotic family proteins (Bik, Bax, Bad, Bim, Bid).

A, 4T1 and MDA-MB 231 cells were treated with lobaplatin. Bik, Bax, Bad, Bim, and Bid were then detected by Western blot analysis. Cells were treated with 0, 10, 20 μg/ml lobaplatin for 24 h

B, 4T1 and MDA-MB 231 cells were treated with microwave hyperthermia at 37℃,39℃, 41℃, and 43℃ for 1 h. Bik, Bax, Bad, Bim, and Bid were detected by Western blot analysis.

C, 4T1 and MDA-MB 231 cells were treated with combination therapy at 37℃, 37℃ and 10 μg/ml lobaplatin, as well as 43℃,43℃ and 10 μg/ml of lobaplatin. 24 h later, Bik, Bax, Bad, Bim, and Bid were detected by Western blot analysis.

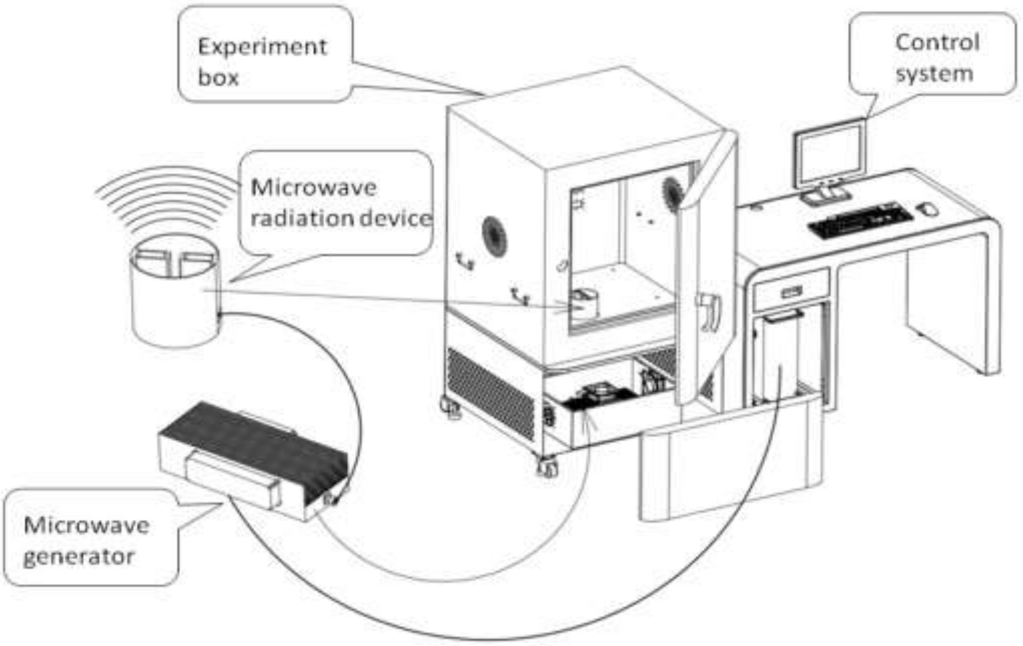

Supplementary 5

Supplementary Fig. 5 The microwave therapy device schematic.

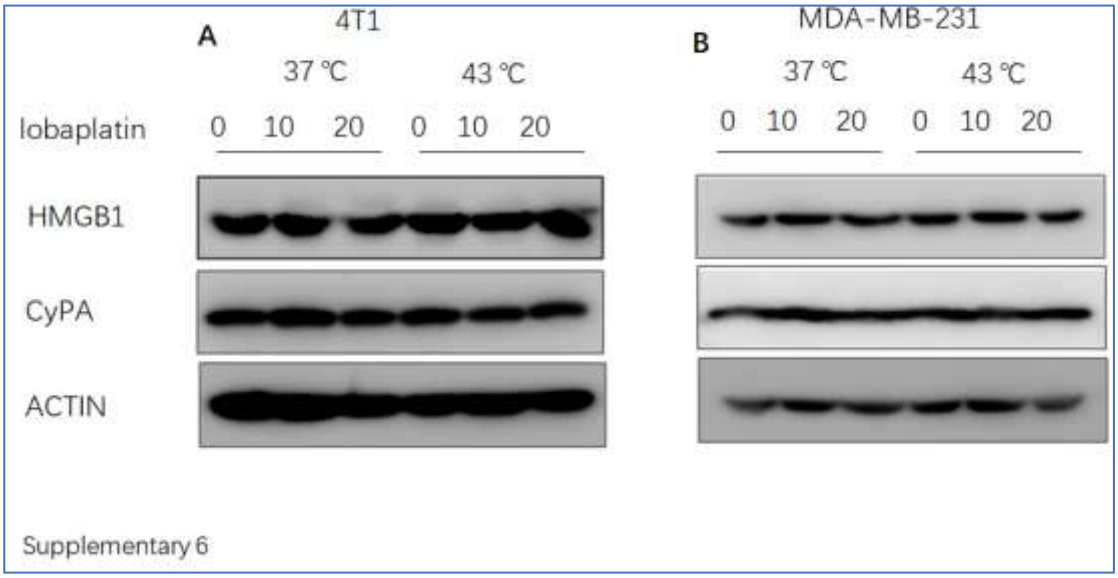

Supplementary Fig. 6 The combination therapy of hyperthermia and lobaplatin do not affect the expression of HMGB1 and CyPA in 4T1 and MDA-MB-231 cells.

4T1 and MDA-MB-231 cells were treated as in Fig. 6, Western blots were used to detect HMGB1 and CyPA.

A, 4T1 cells were treated in either the absence or presence of lobaplatin (10 μg/ml) or at an indicated temperature.

B, MDA-MB 231 cells were treated in either the absence or presence of lobaplatin (10 μg/ml) or at an indicated temperature.

24 h later, HMGB1and CyPA were detected by Western blot analysis.

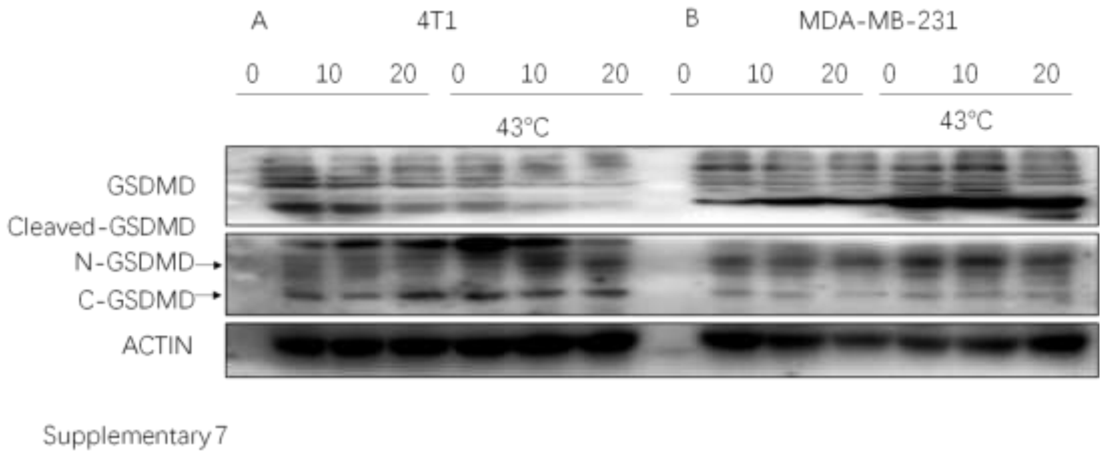

Supplementary Fig. 7 The combination therapy of hyperthermia and lobaplatin obviously affect the expression of GSDMD in 4T1 and MDA-MB-231 cells.

A, 4T1 cells were treated in either the absence or presence of lobaplatin (10 μg/ml) or at an indicated temperature.

B, MDA-MB 231 cells were treated in either the absence or presence of lobaplatin (10 μg/ml) or at an indicated temperature.

24 h later, GSDMD was detected by Western blot analysis.
