# Supplementary material for: The anti-tumor effects of the combination of microwave hyperthermia and lobaplatin against breast cancer cells in vitro and in vivo
Source: Biosci Rep. 2022 Feb 9;42(2):BSR20190878. doi: 10.1042/BSR20190878 (PMC8829017; doi:10.1042/BSR20190878)

Lobaplatin

Fig4B PARP 116KD,89KD

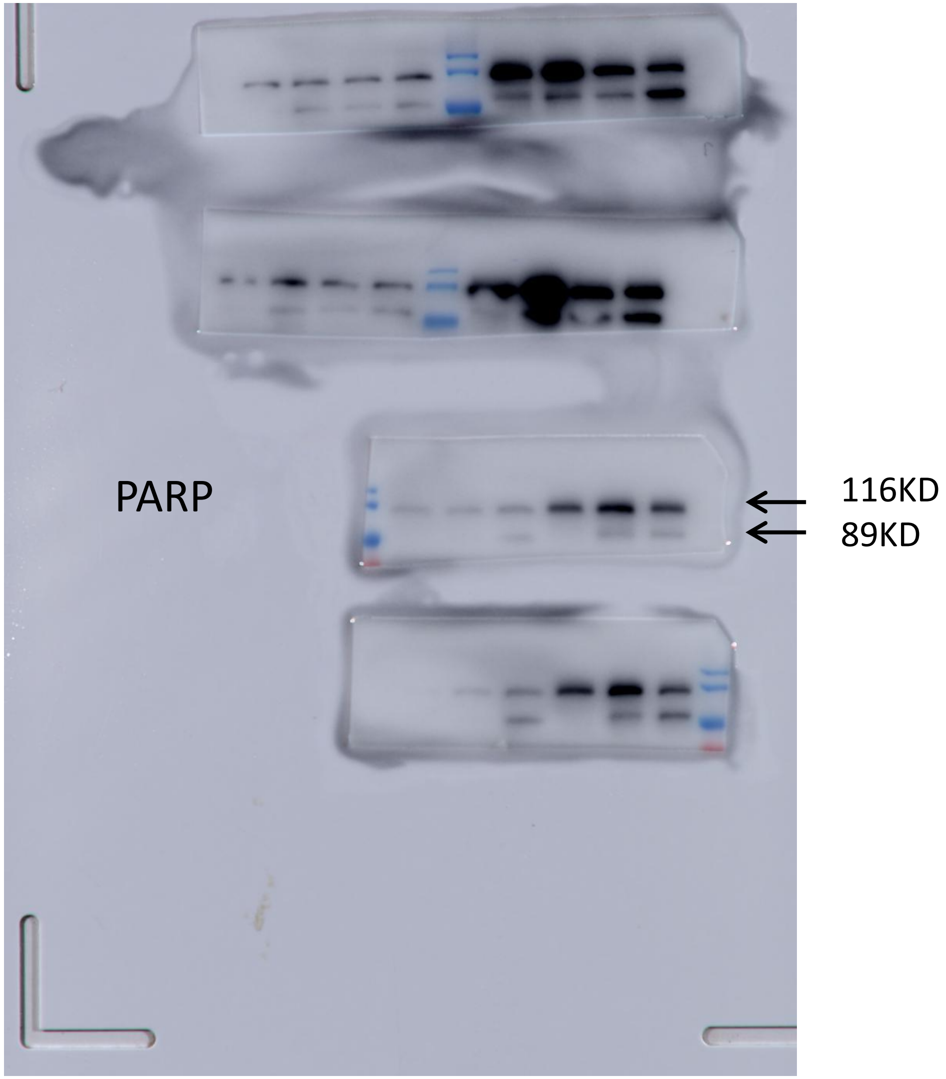

Fig4B Caspse3

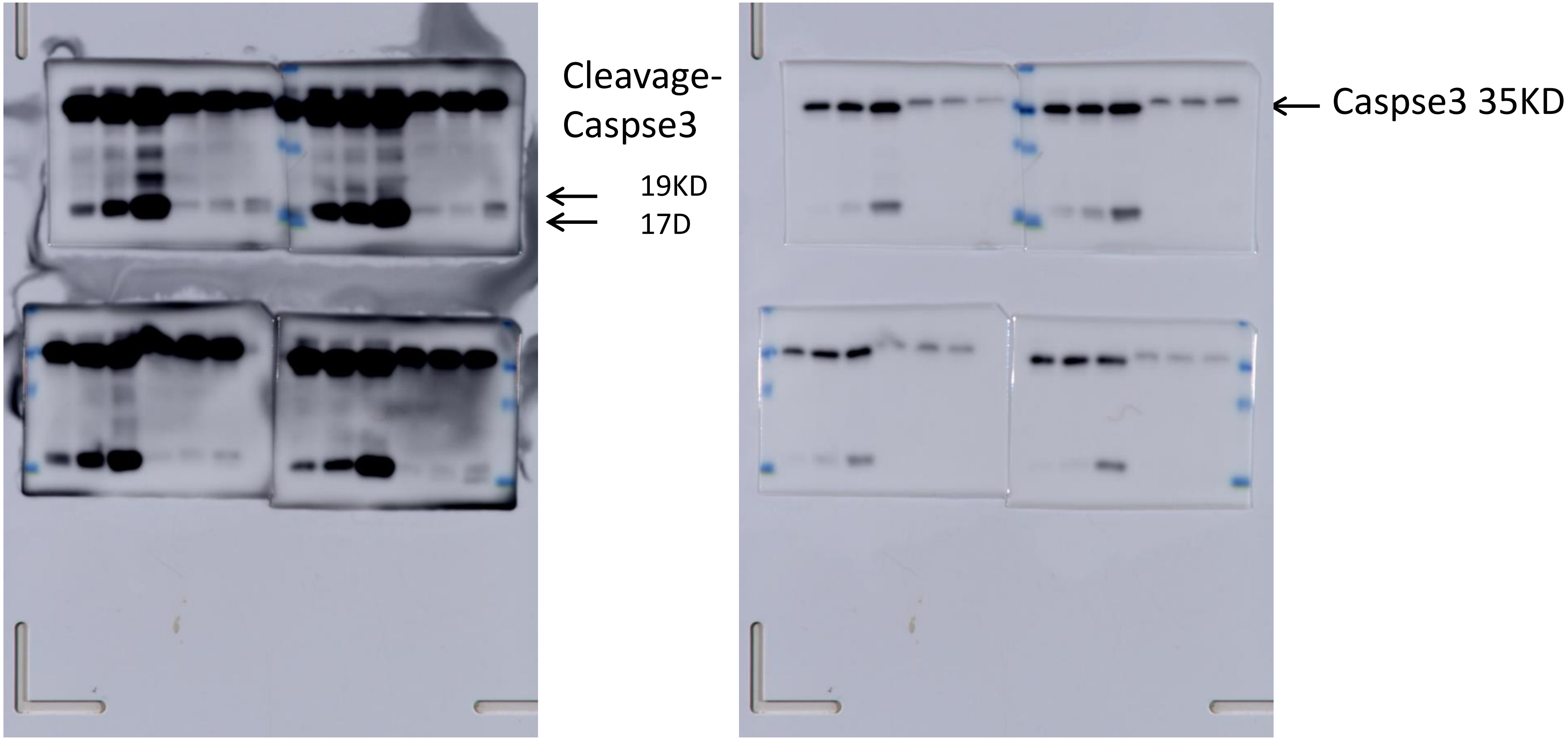

Fig4B actin 45KD

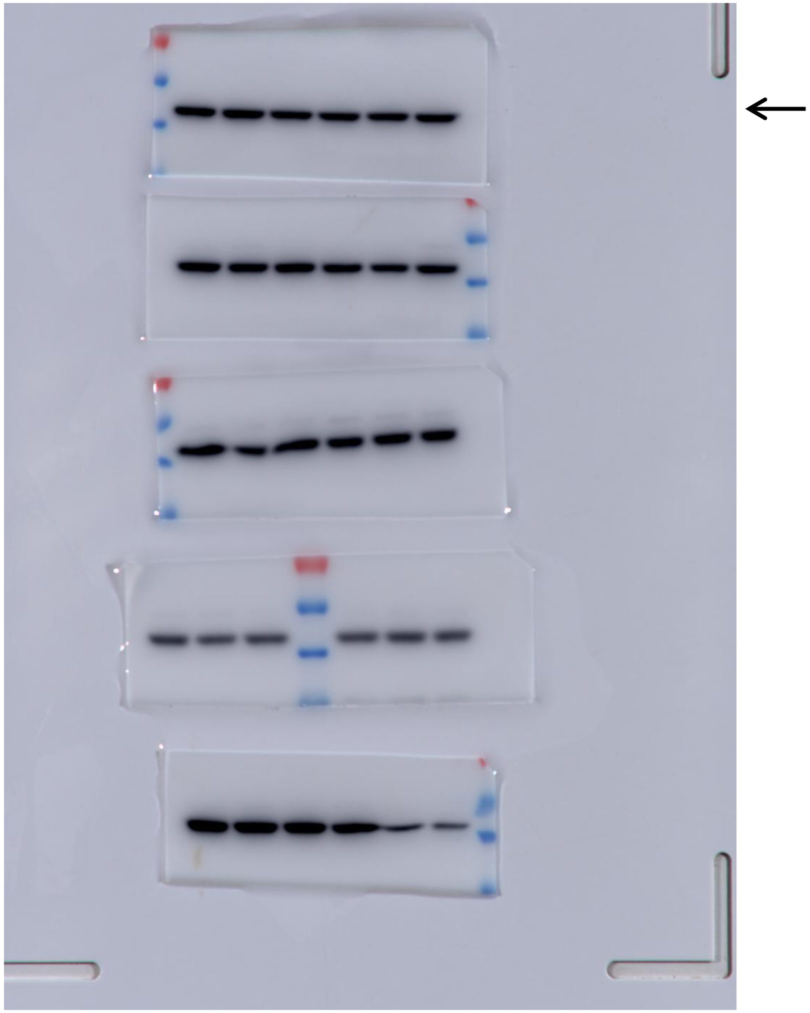

Fig4B P62

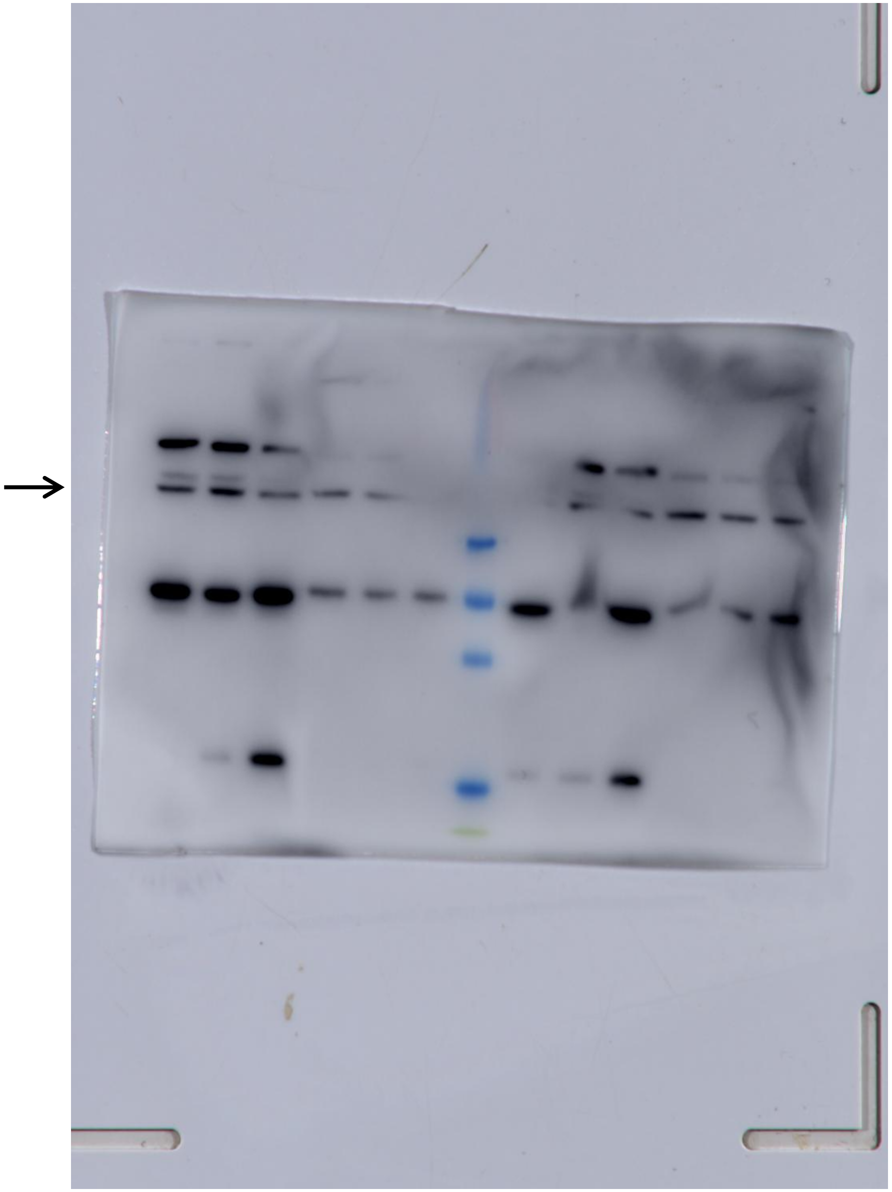

P62，62KD

Fig4B LC3

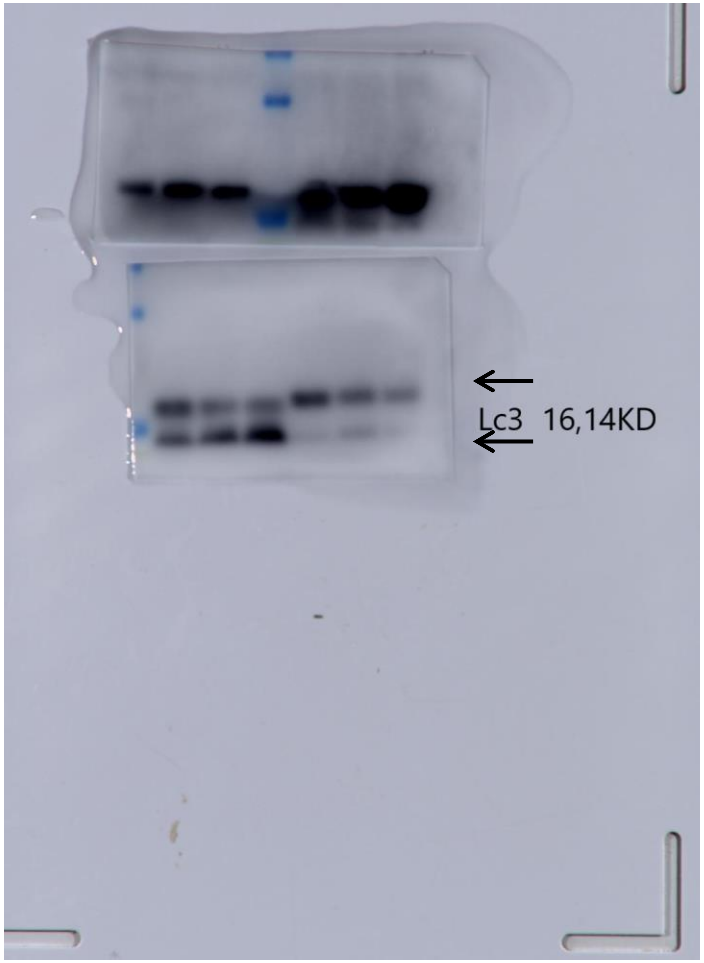

LC3

Fig4B actin 45KD

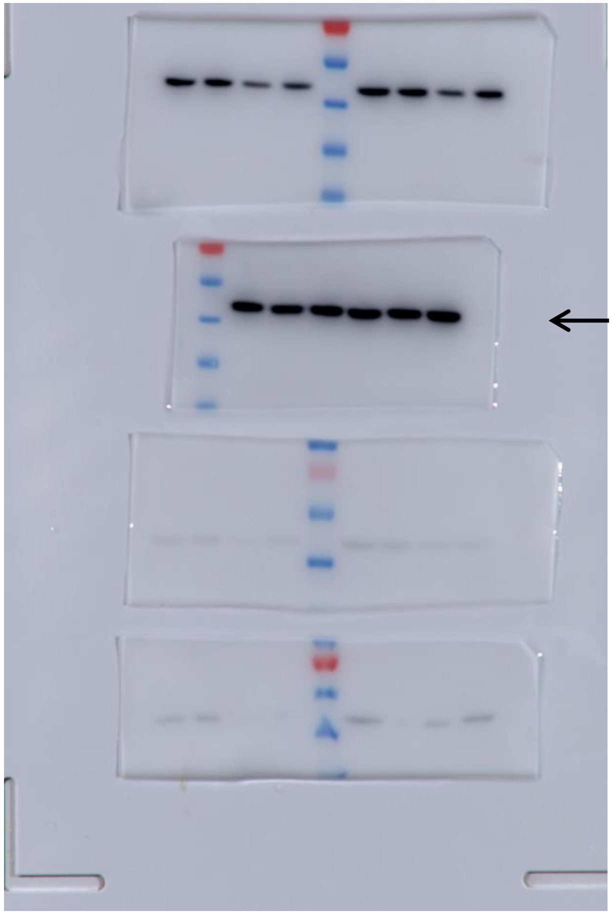

← actin 45KD

Fig5A P-AMPK

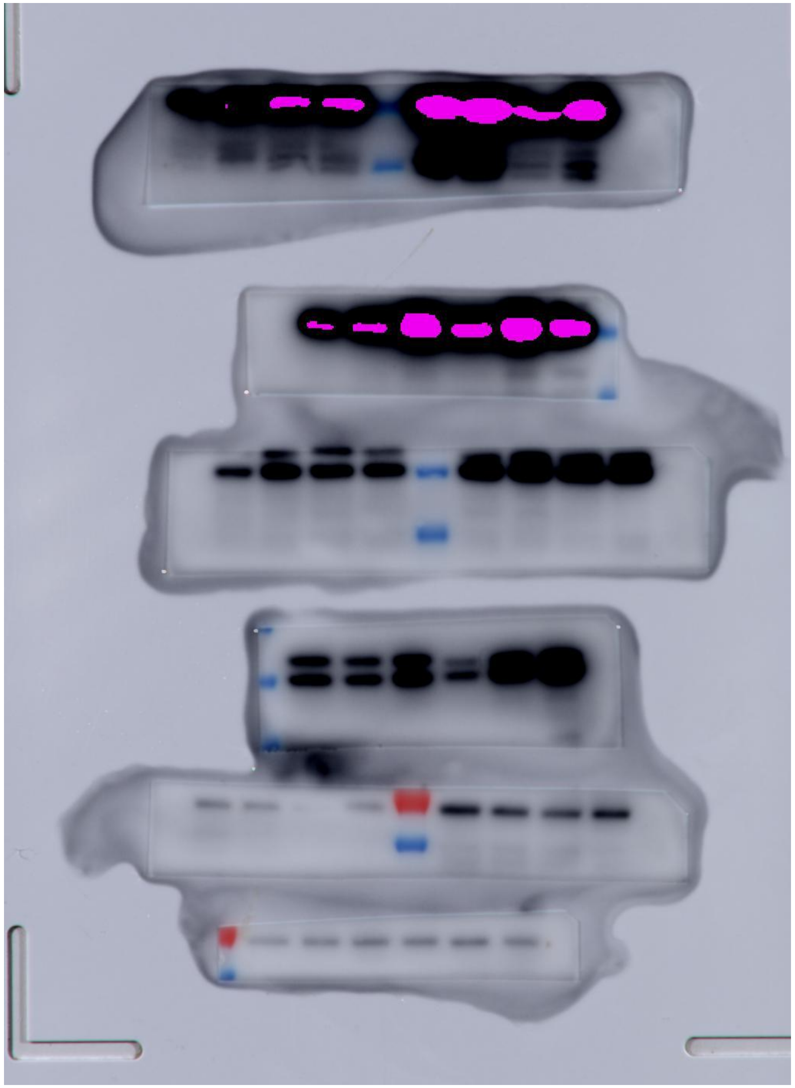

← Fig5A P-AMPK 62KD

Fig5A AMPK

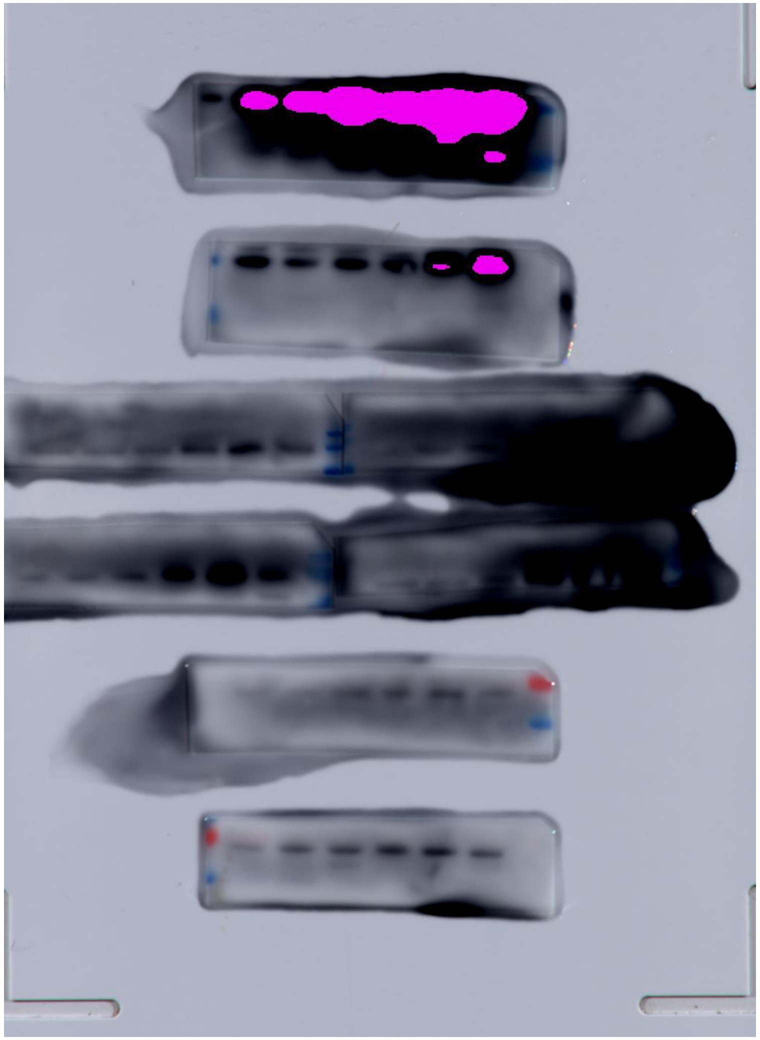

← AMPK 62KD

Fig5A P-AKT 308 60KD

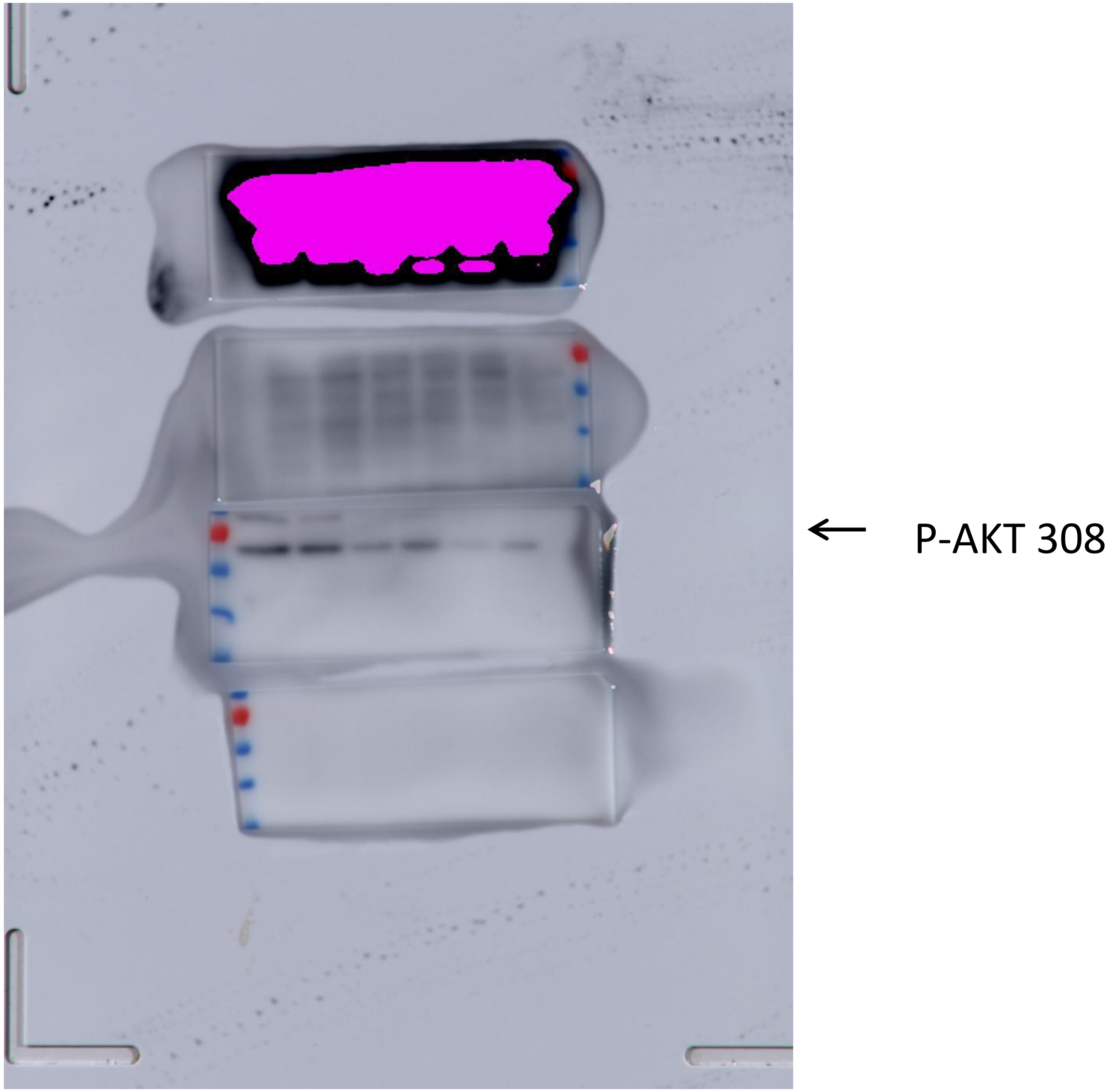

Fig5A P-AKT 473

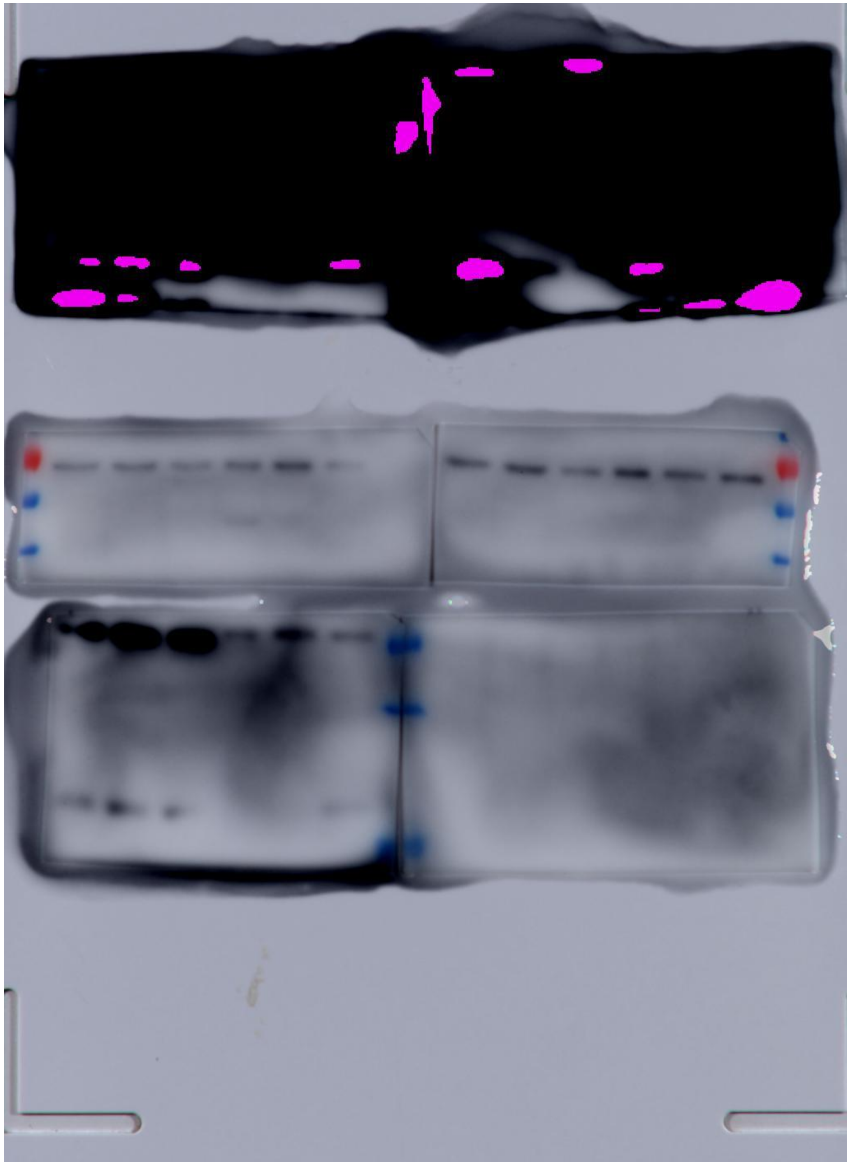

← P-AKT 473 60KD

Fig5A AKT

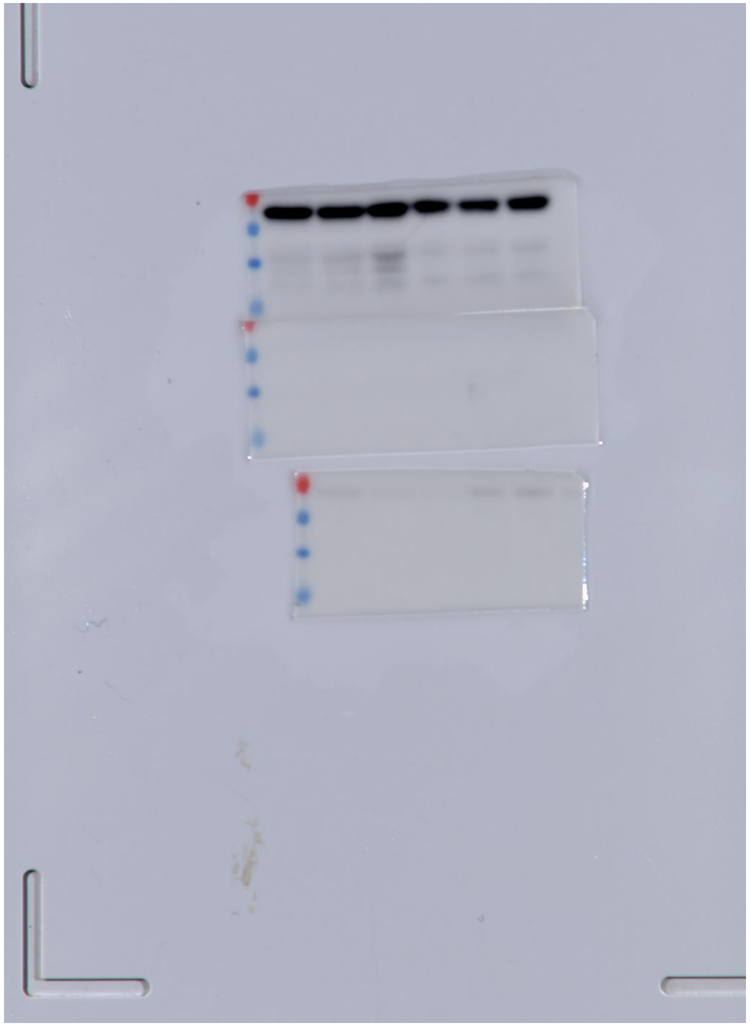

← AKT 60KD

Fig5A mTOR

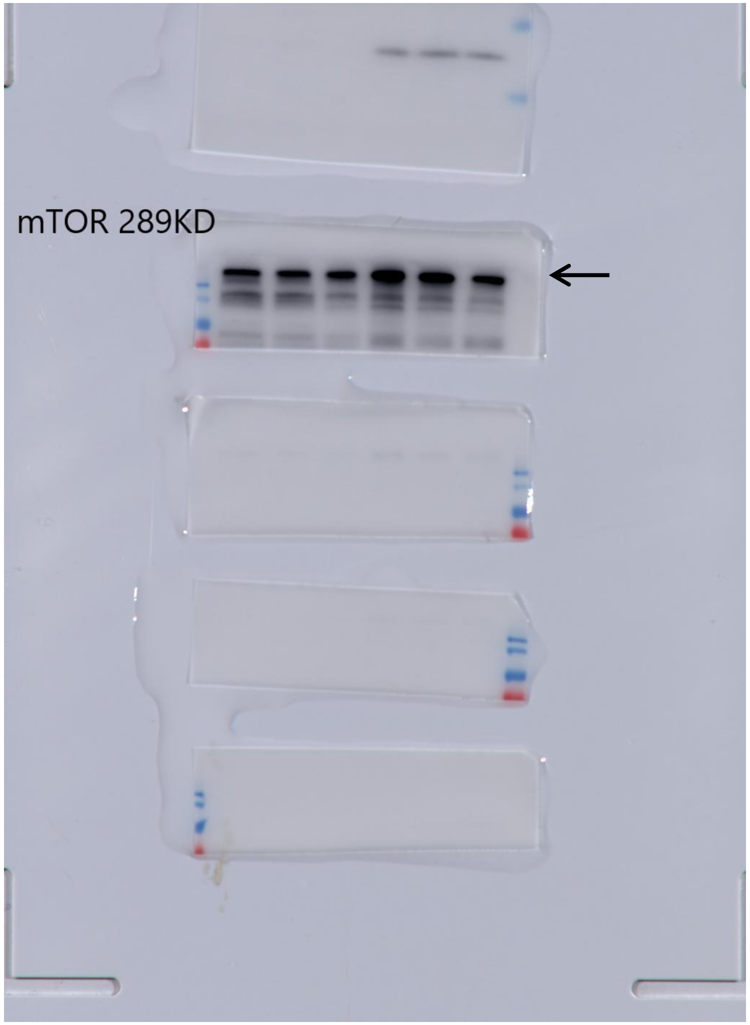

Fig5A p-mTOR2448 2481 289KD

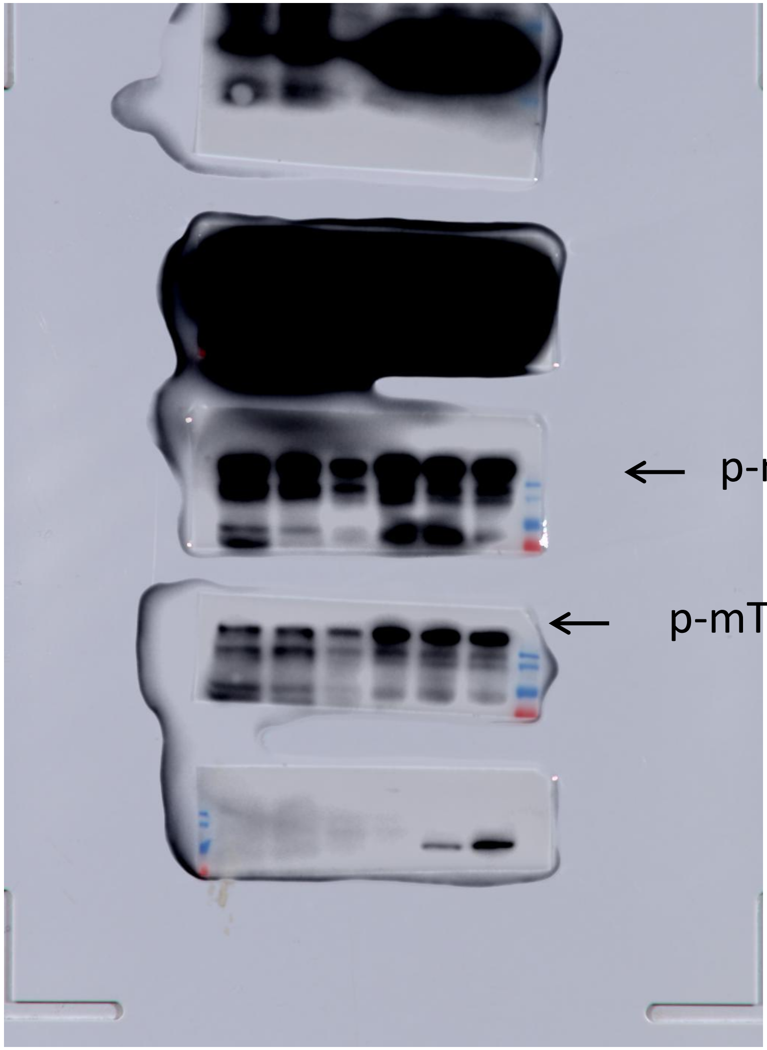

← p-mTOR2448 289KD

← p-mTOR2481 289KD

Fig5A P-P70S6K

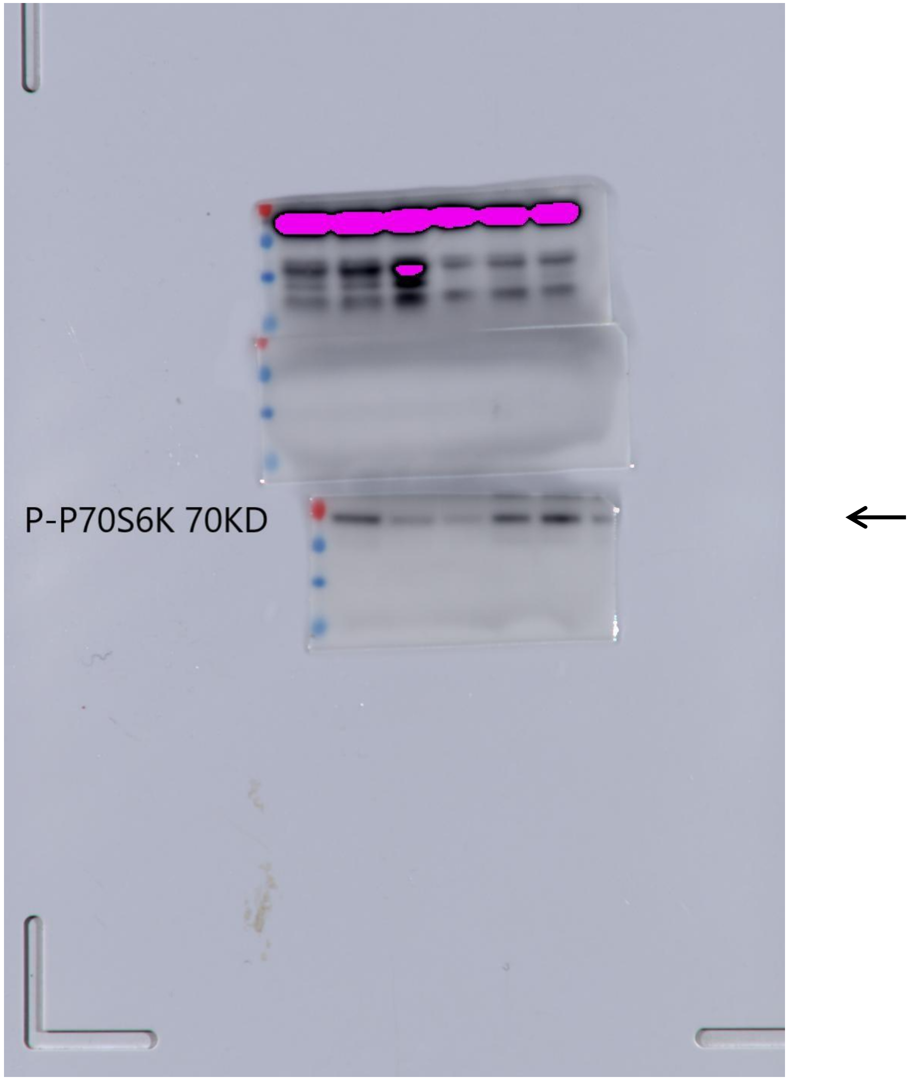

Fig5A P70S6K

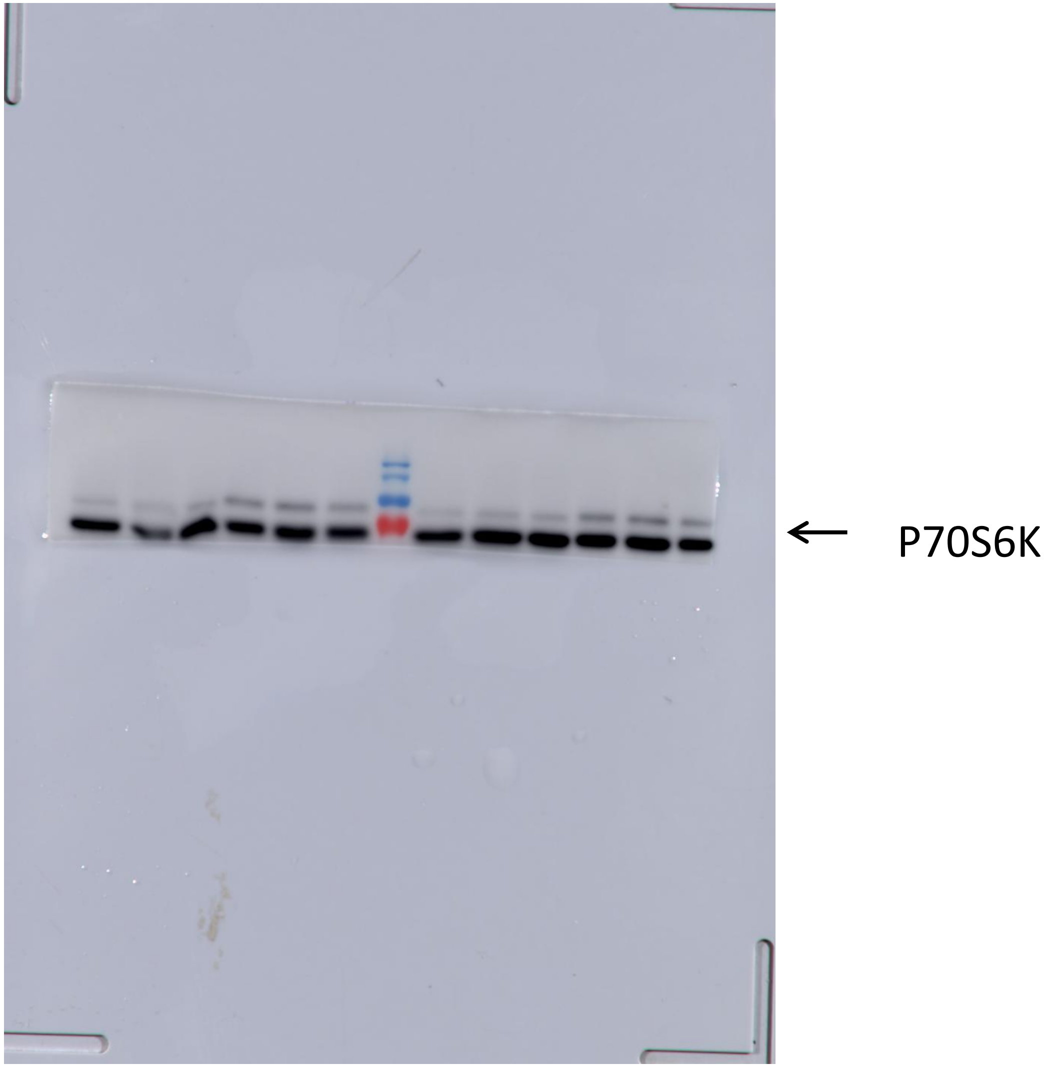

Fig5A actin 45KD

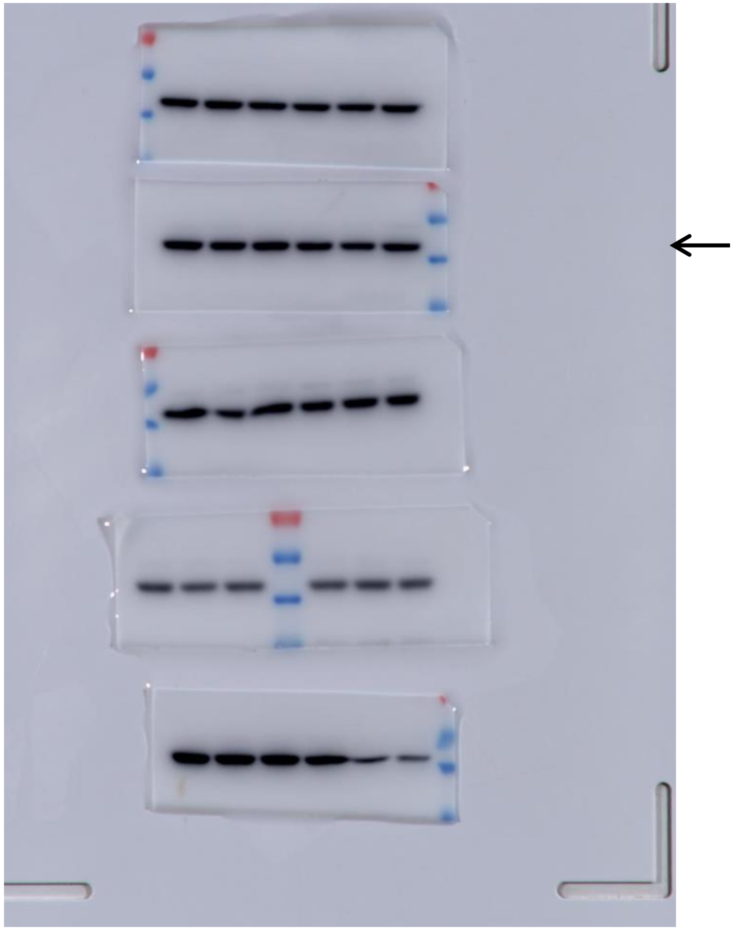

Fig5A P-ERK

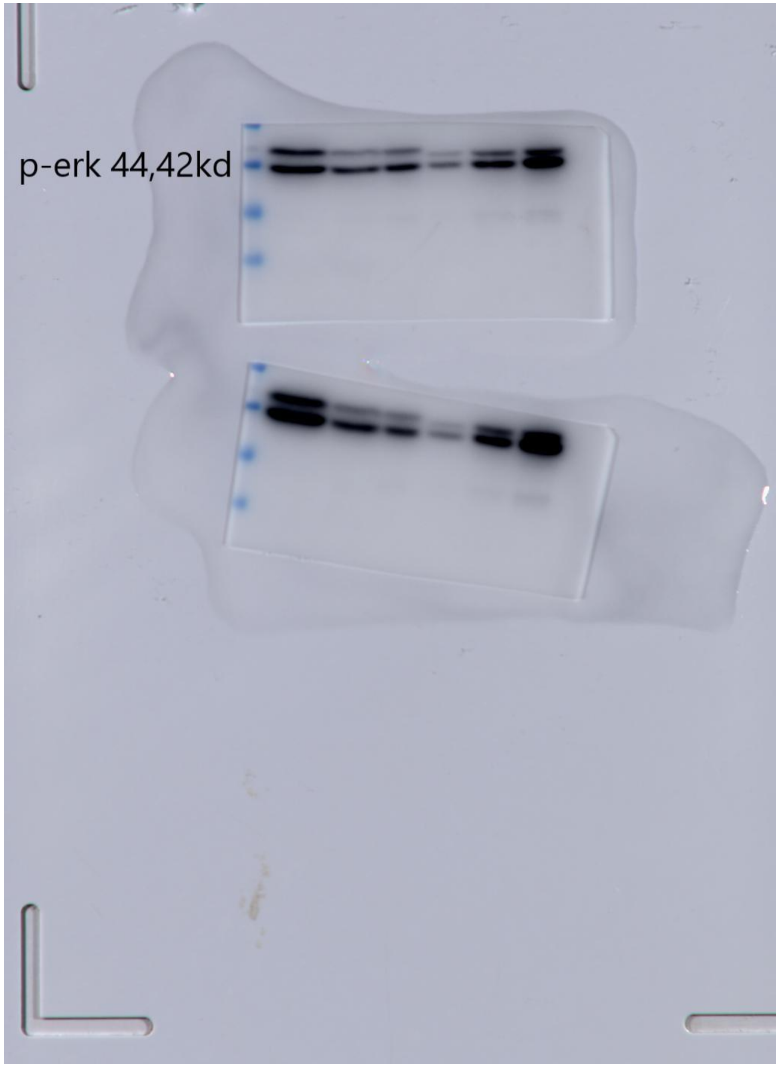

Fig5A P-P38

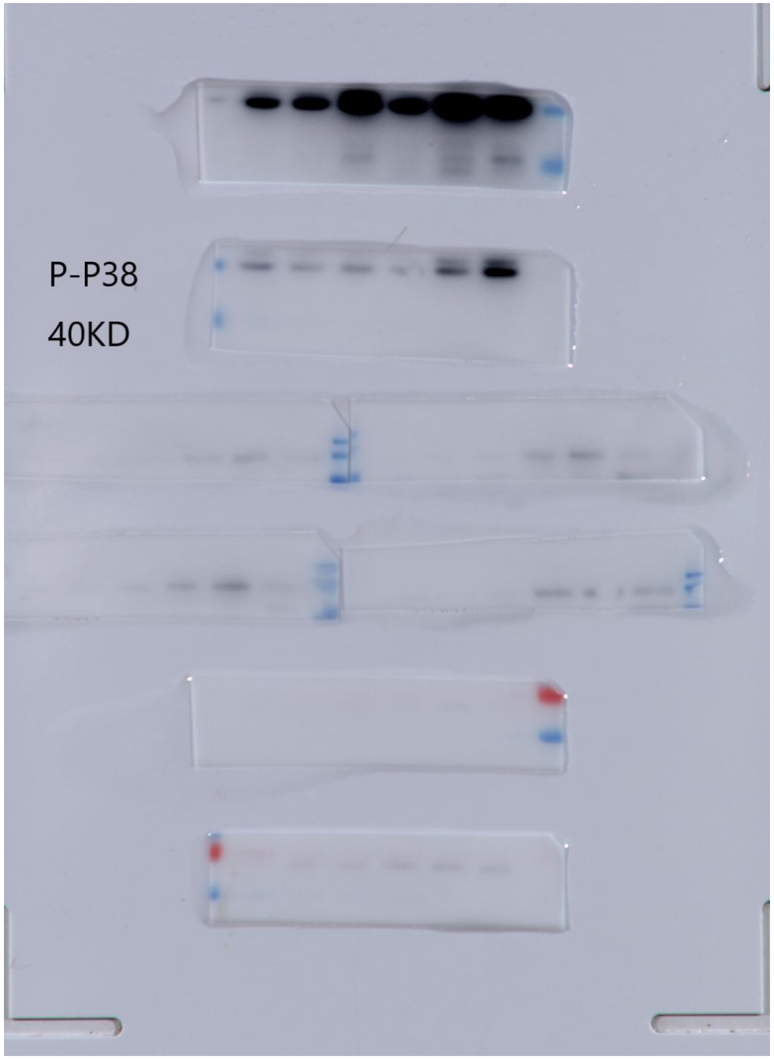

Fig5A p-JNK， JNK

JNK

P-JNK

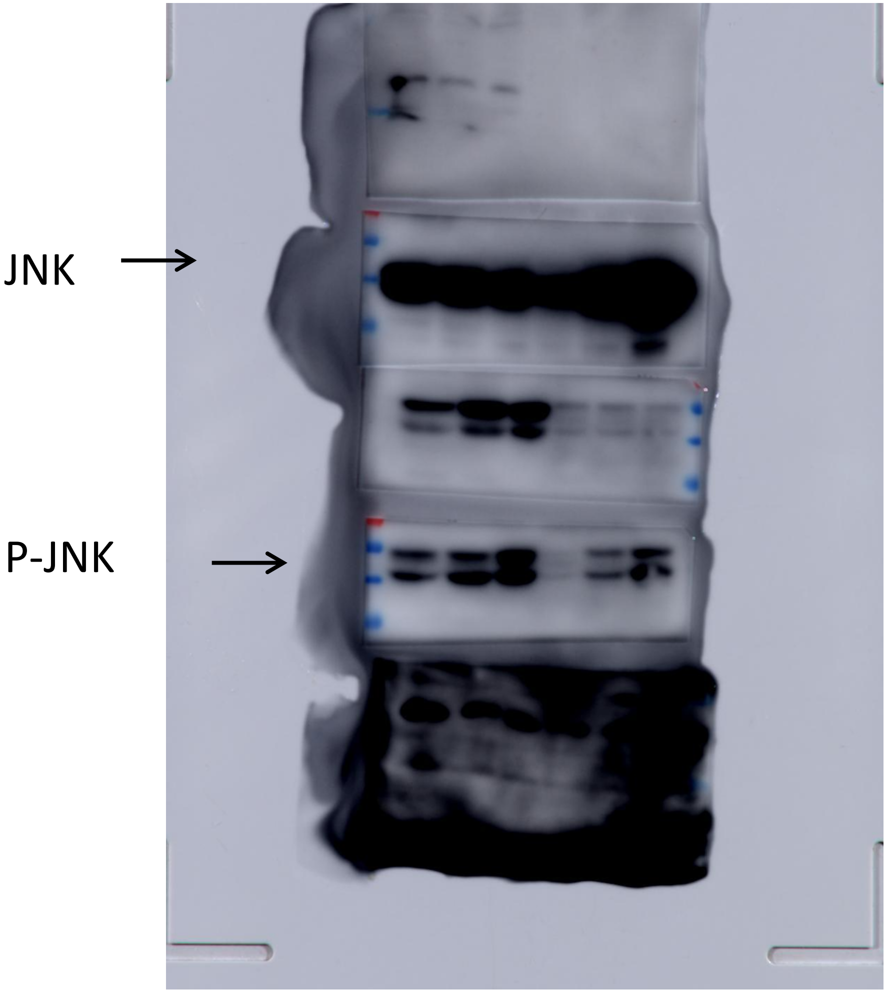

JNK

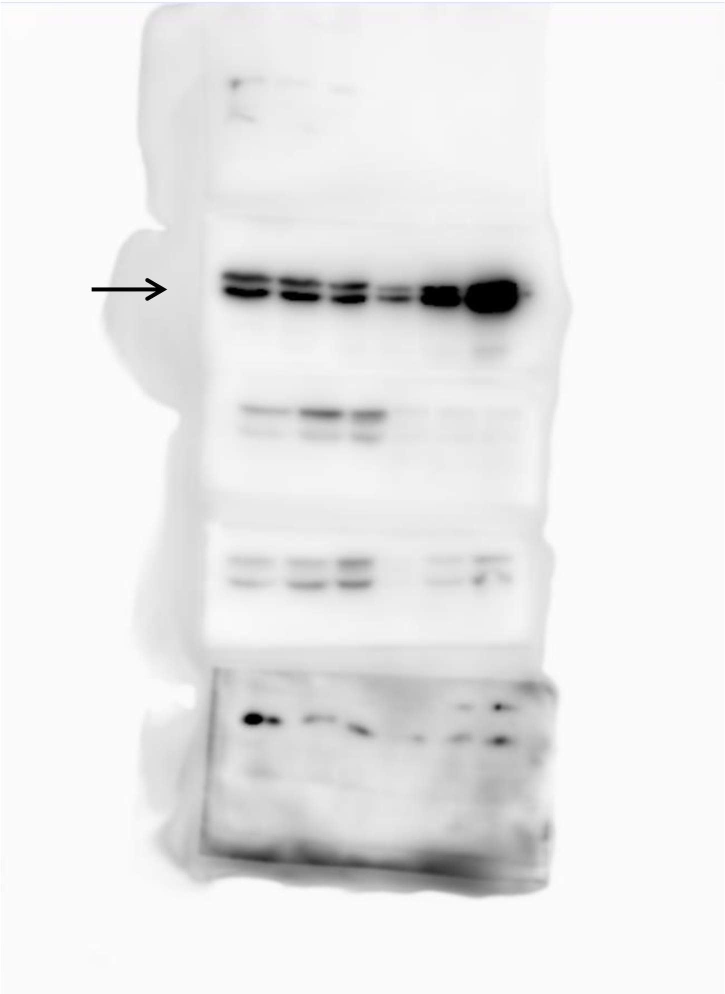

Fig5A actin

→ 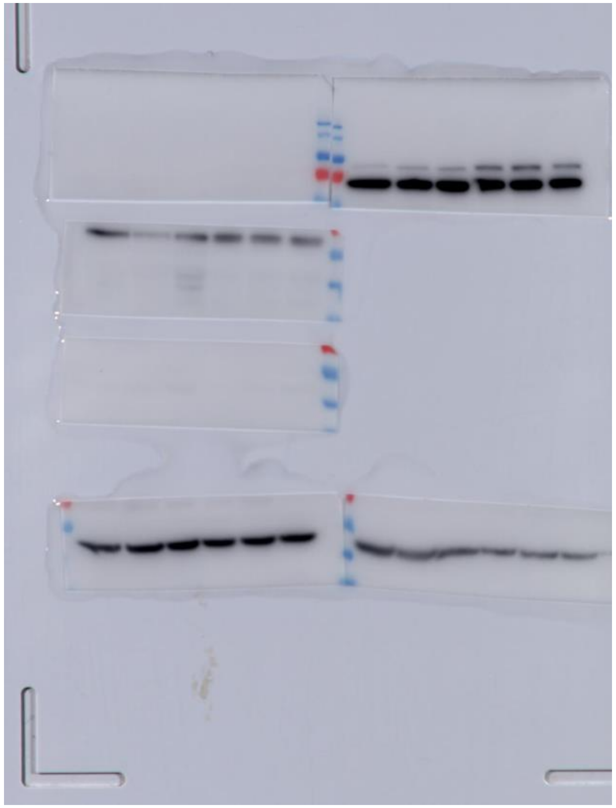 actin

Fig6A mcl-1

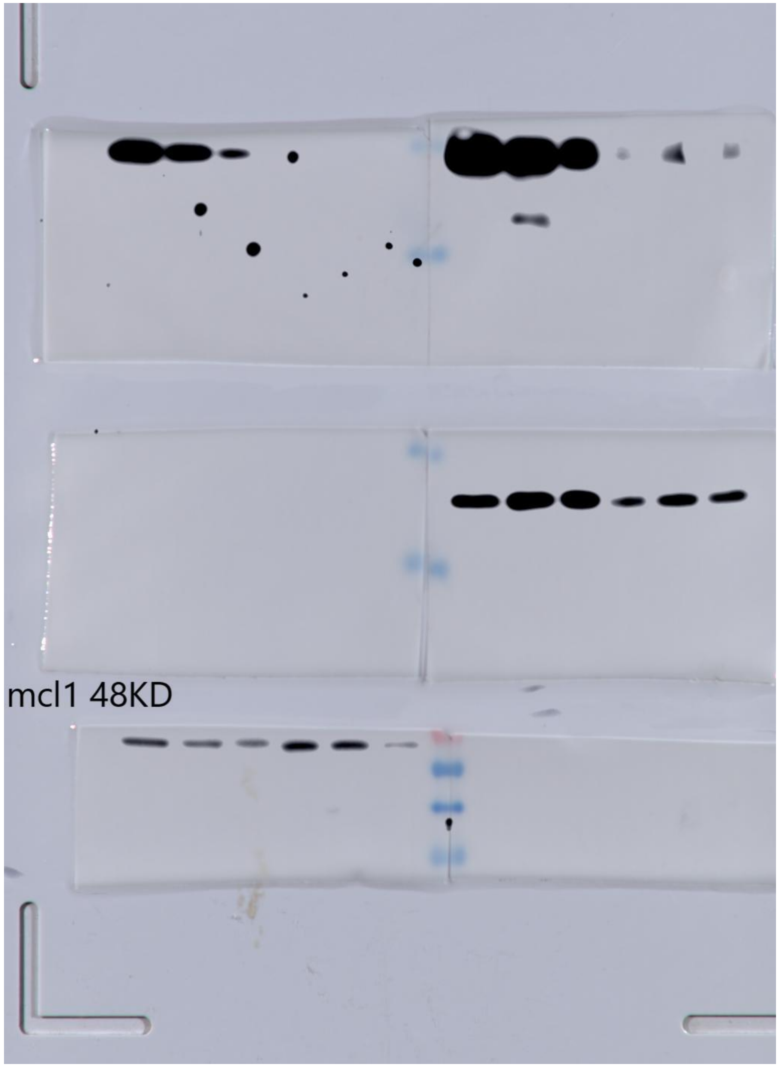

Fig6A BCL-XL

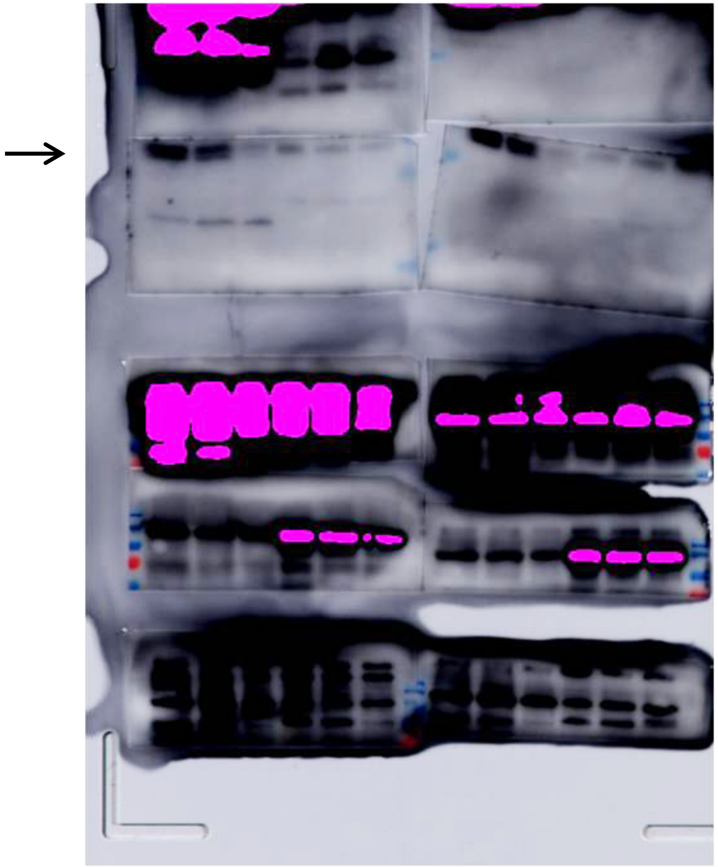

BCL-XL 30KD

Fig6A BCL2

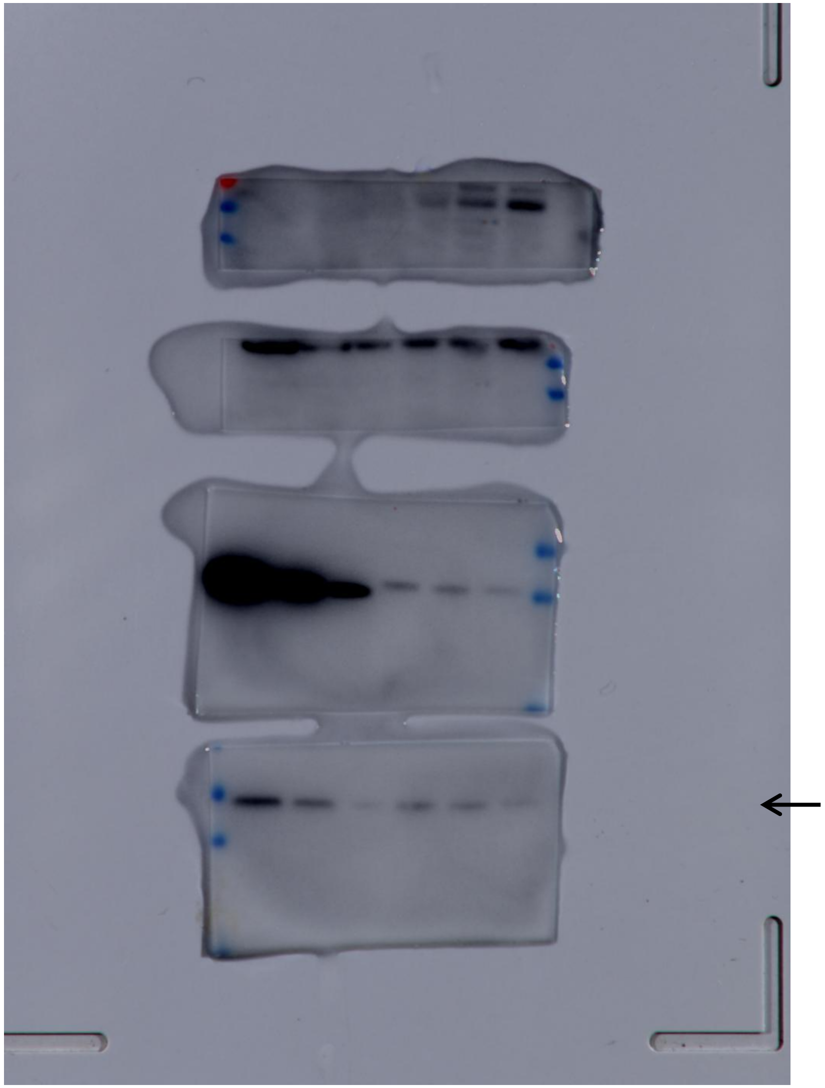

← BCL2 26KD

Fig6A actin 45KD

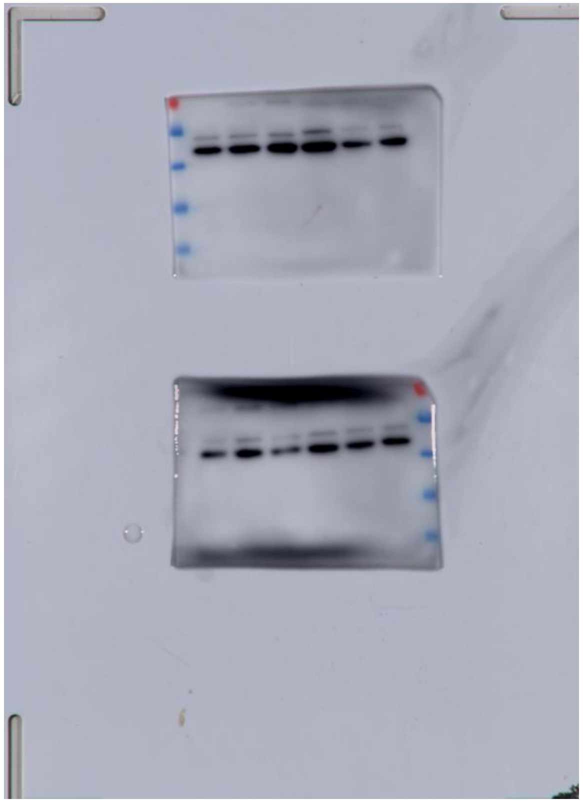

← actin

Fig6A C-IAP1， C-IAP2， XIAP

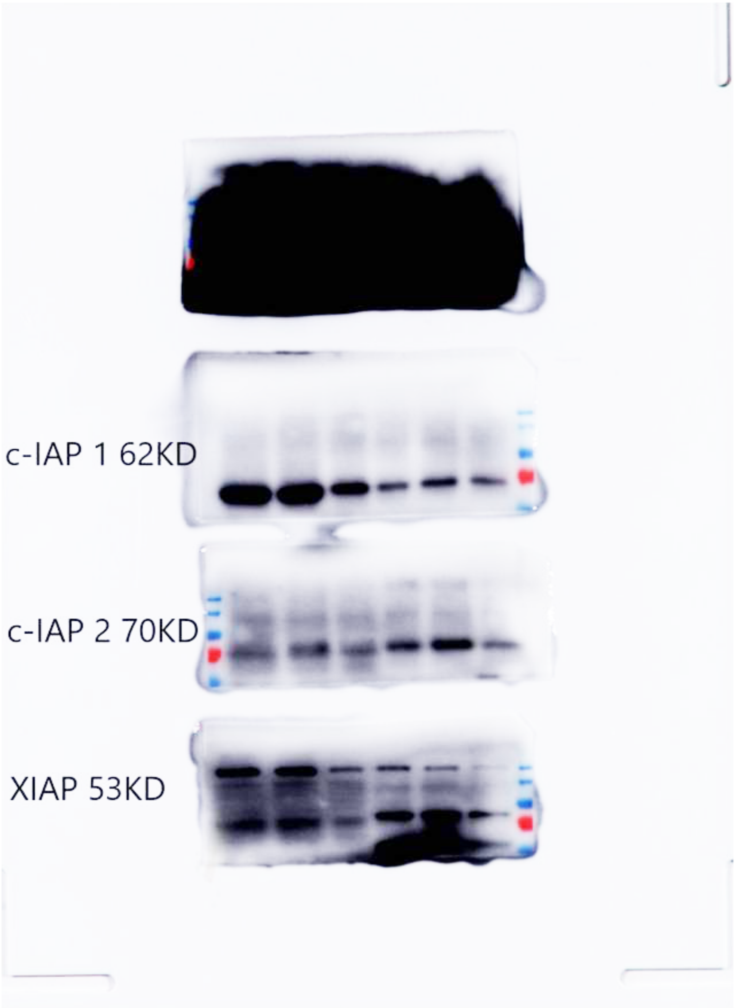

Fig6A actin 45KD

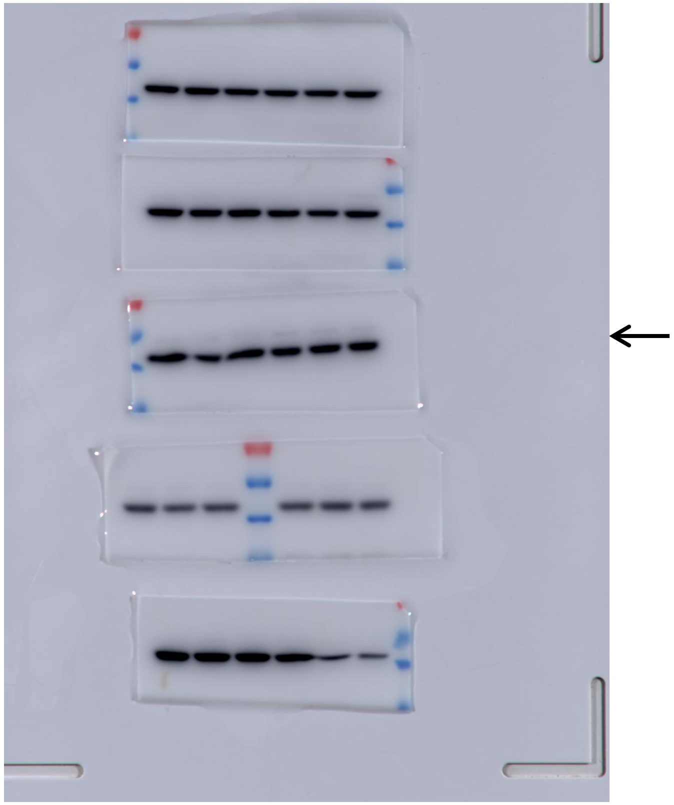

Supplementary 4A Bim

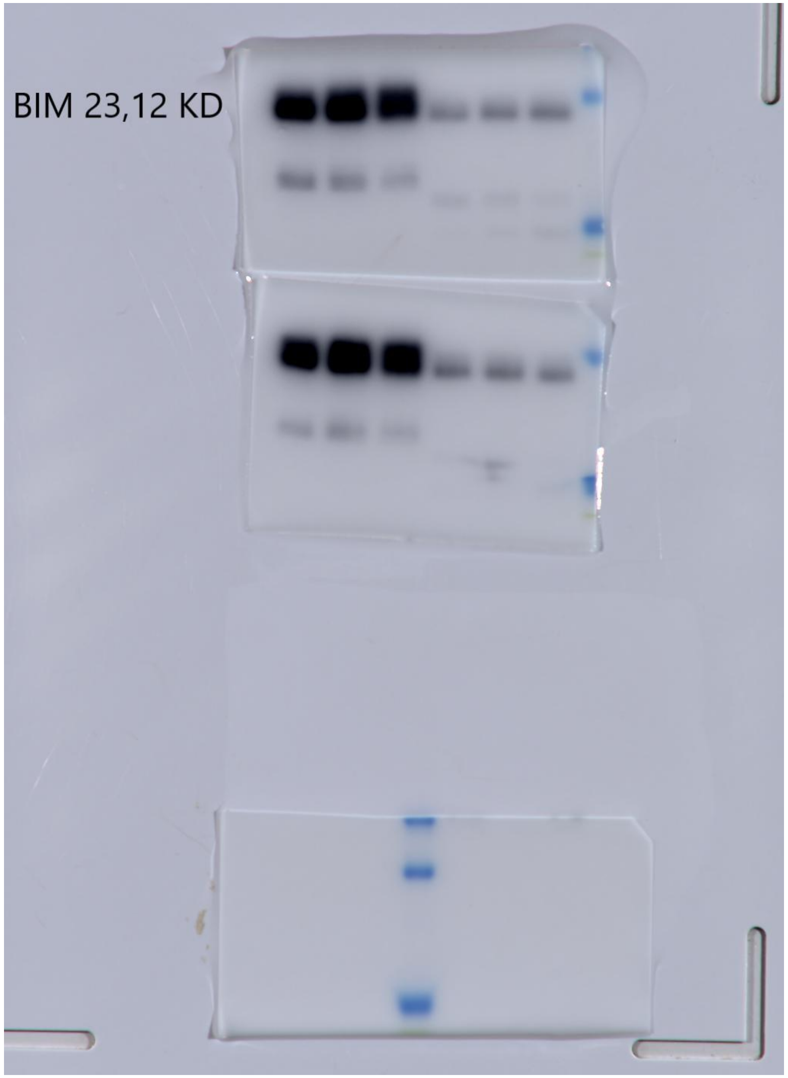

Supplementary 4A Bax

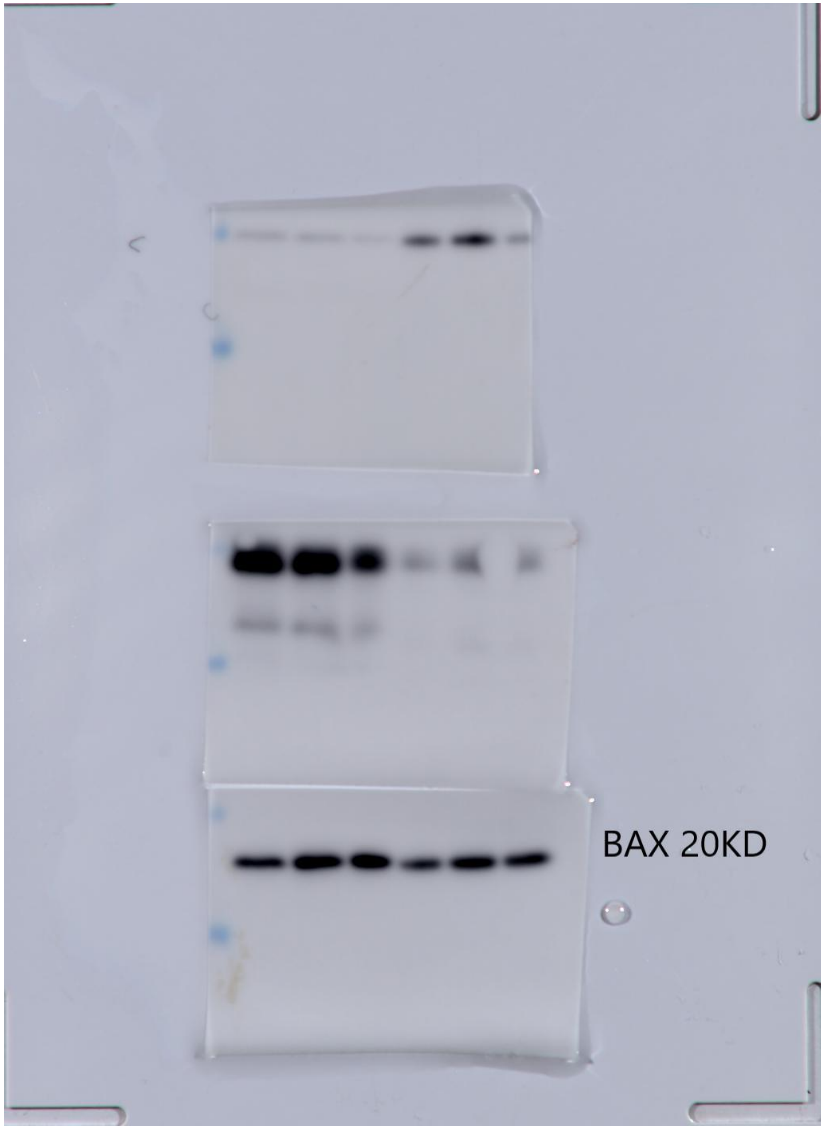

Supplementary 4A Bak

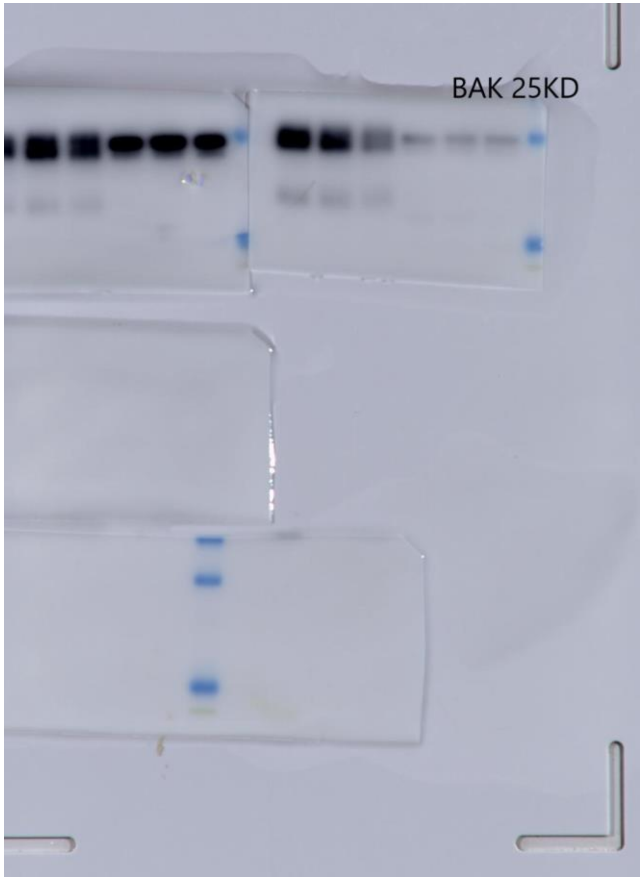

Supplementary 4A Bid

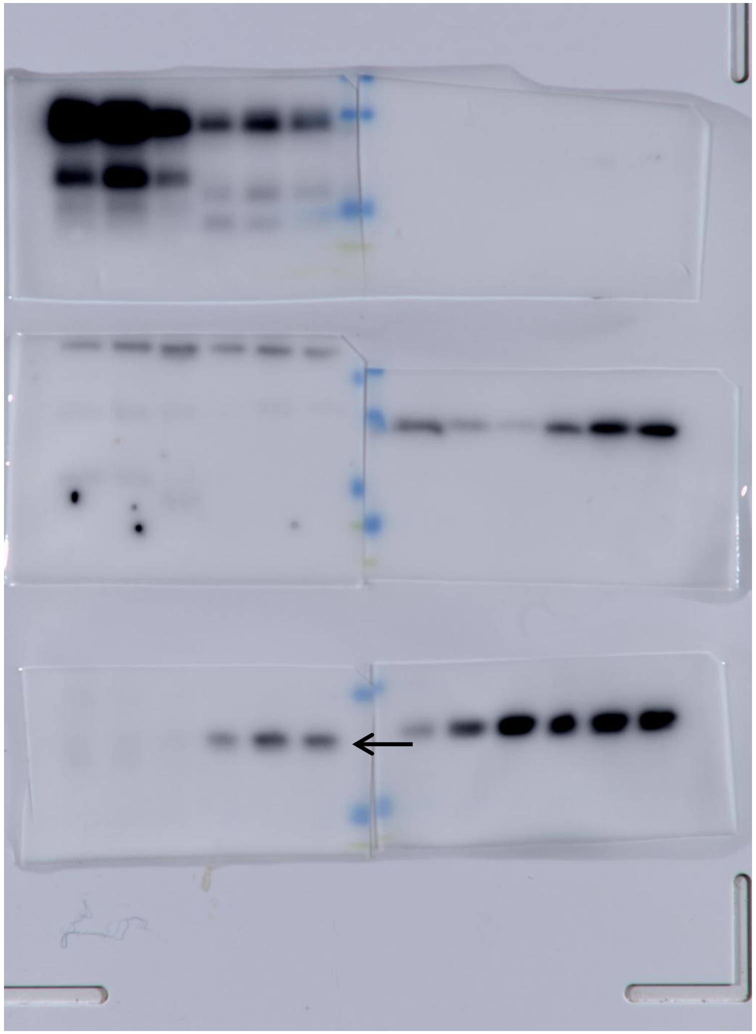

Bid 22KD

Supplementary 4A Bad 23KD

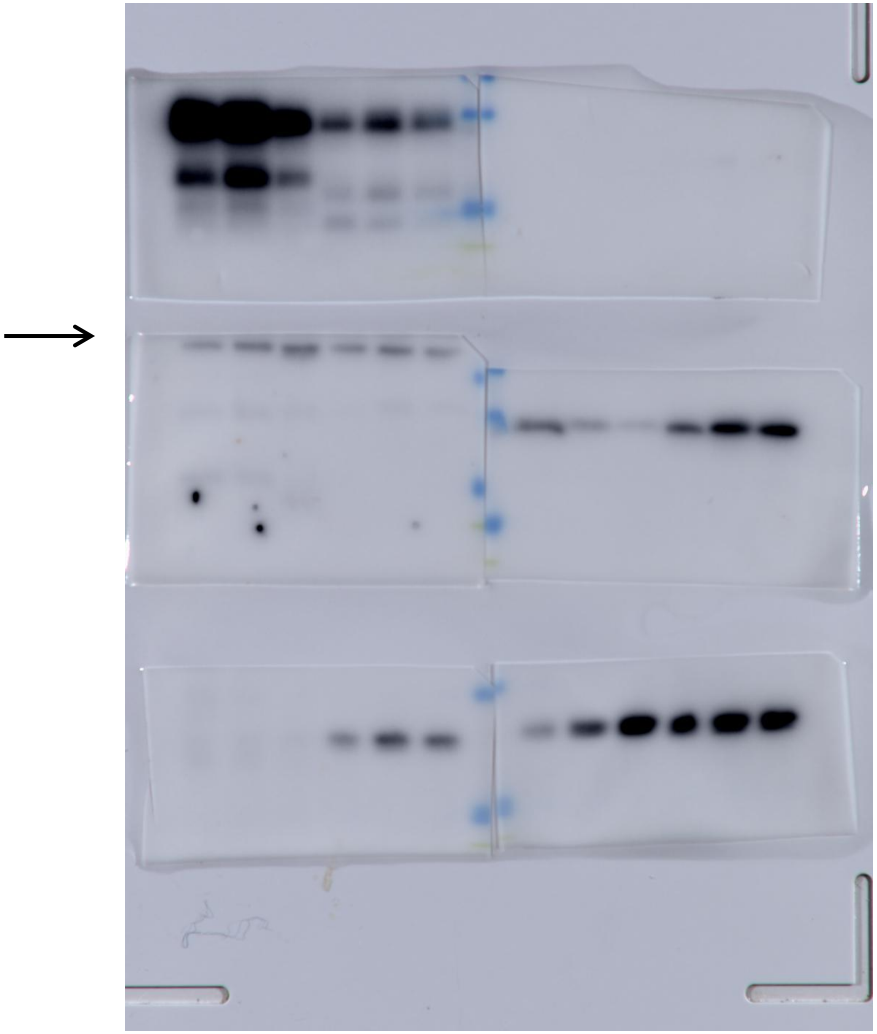

Bad 23KD

Supplementary 4A actin

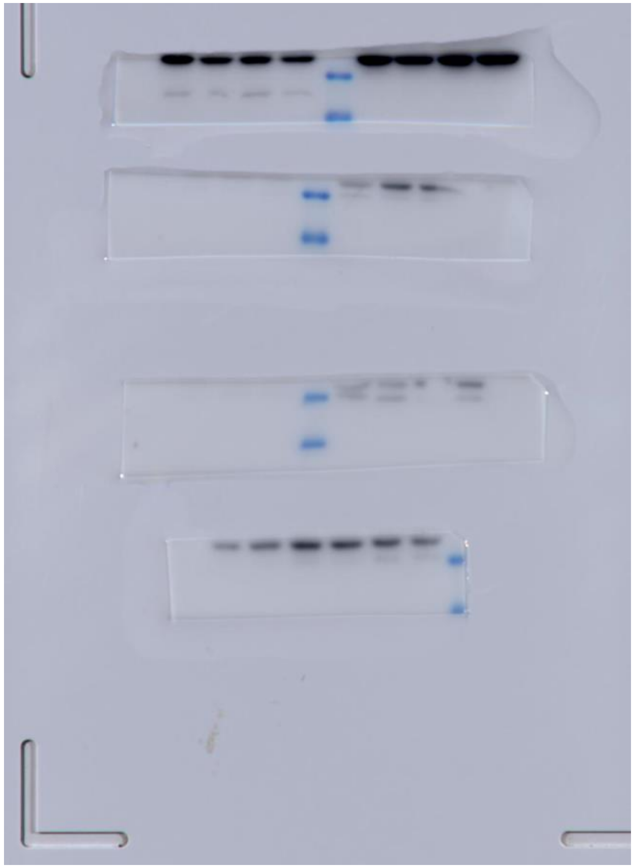

← actin

Hyperthermia(H)

Fig4C PARP 116, 89KD

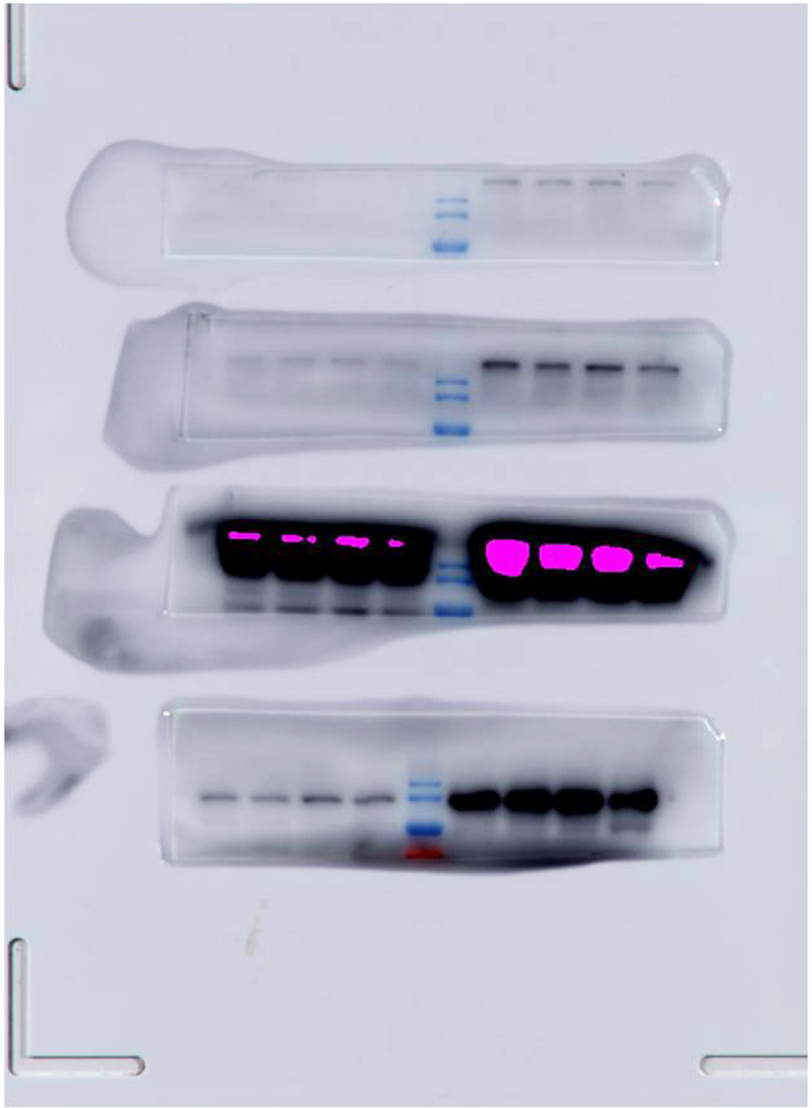

Fig4C Caspase3 35,19,17KD

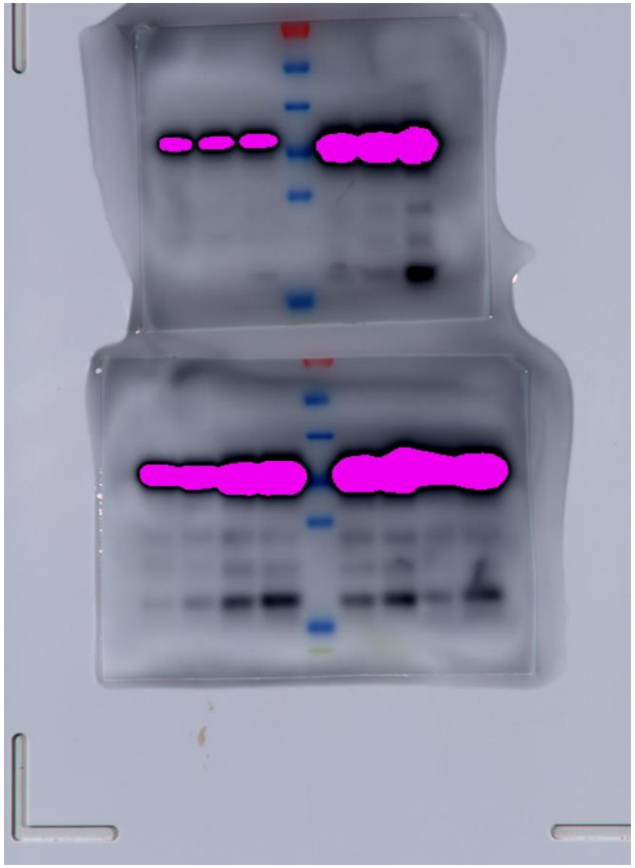

Cleavage
Caspase3
19,17KD

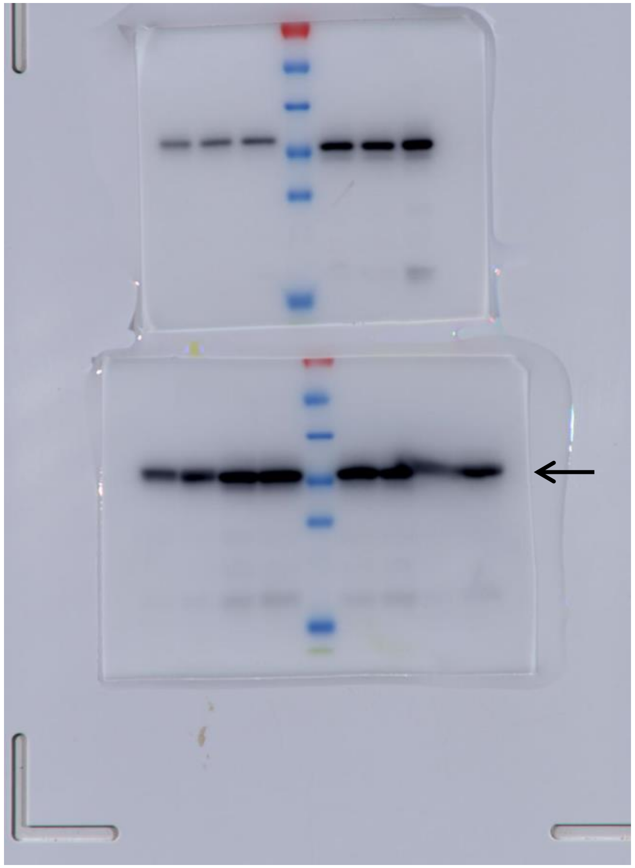

Caspase3
35KD

Fig4C actin 45KD

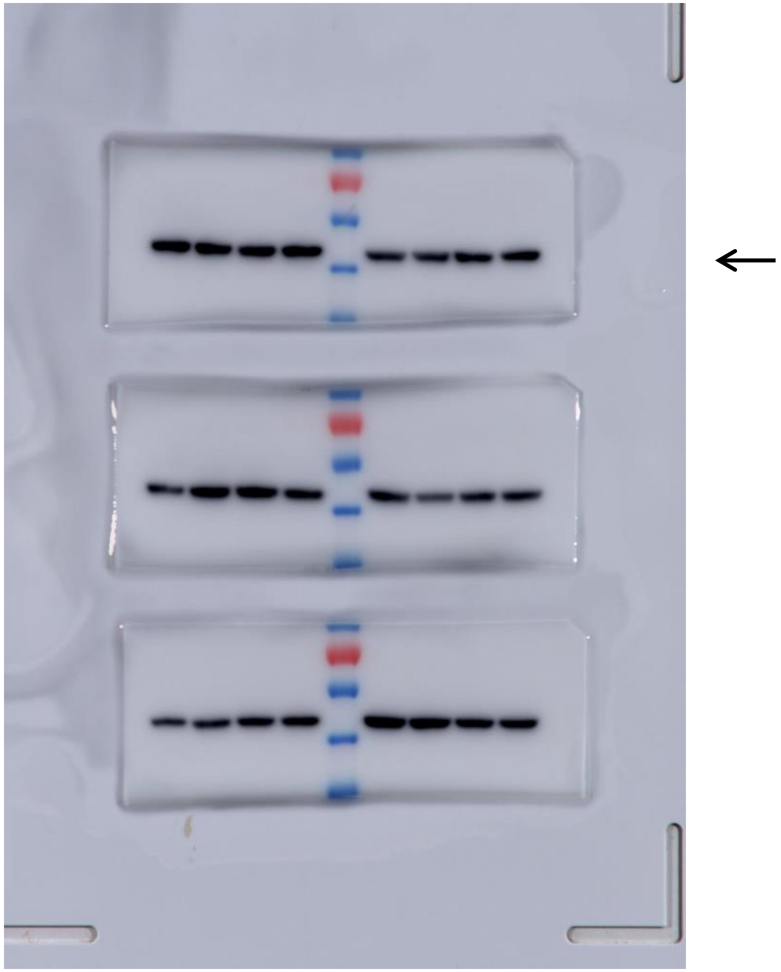

Fig4C P62 62KD

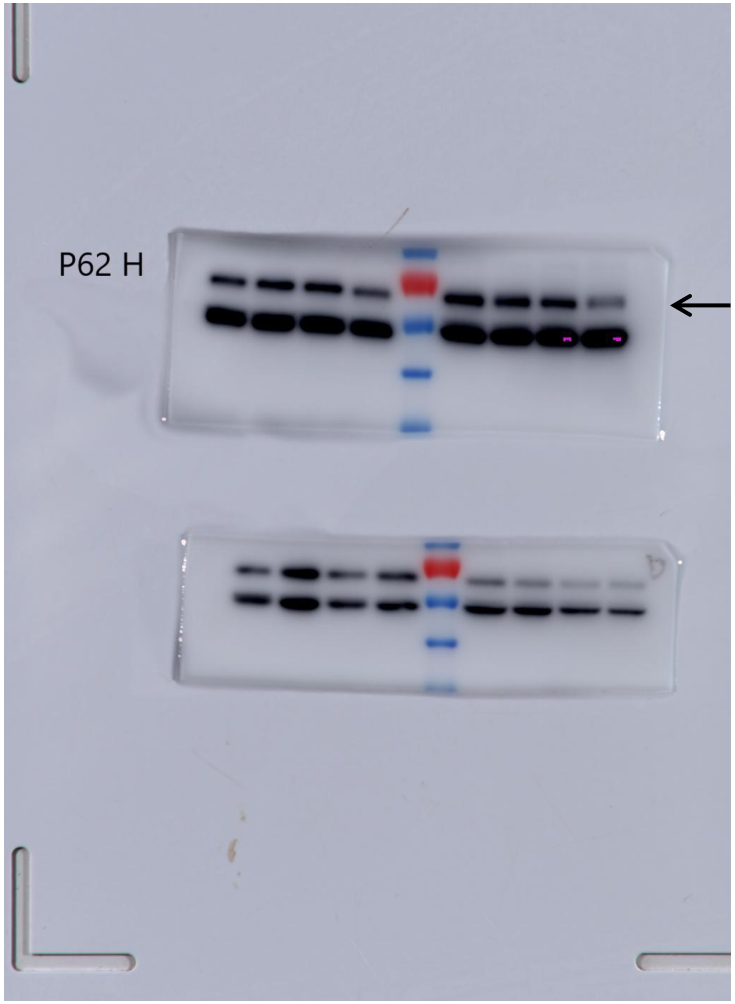

Fig4C LC3 16,14KD

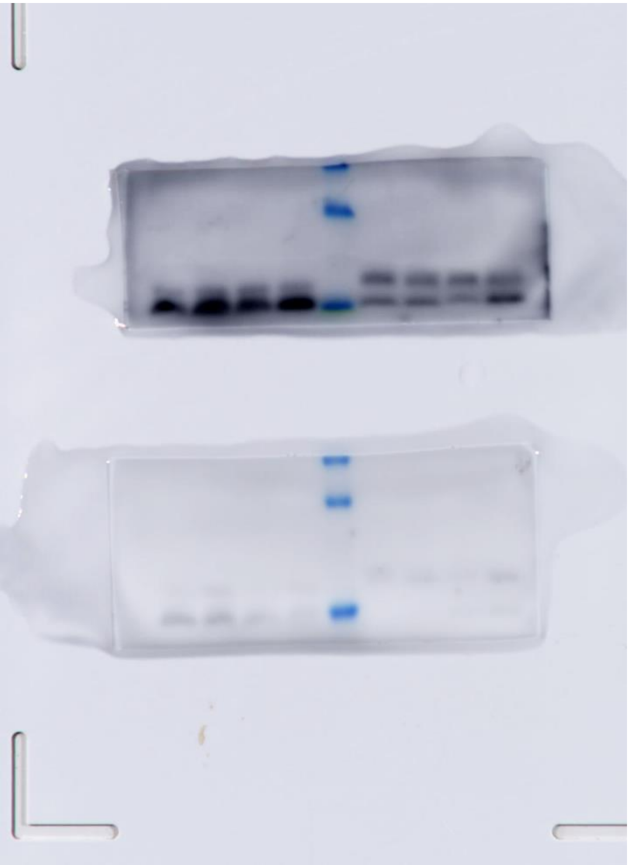

← LC3 16,14KD

Fig4C actin 45KD

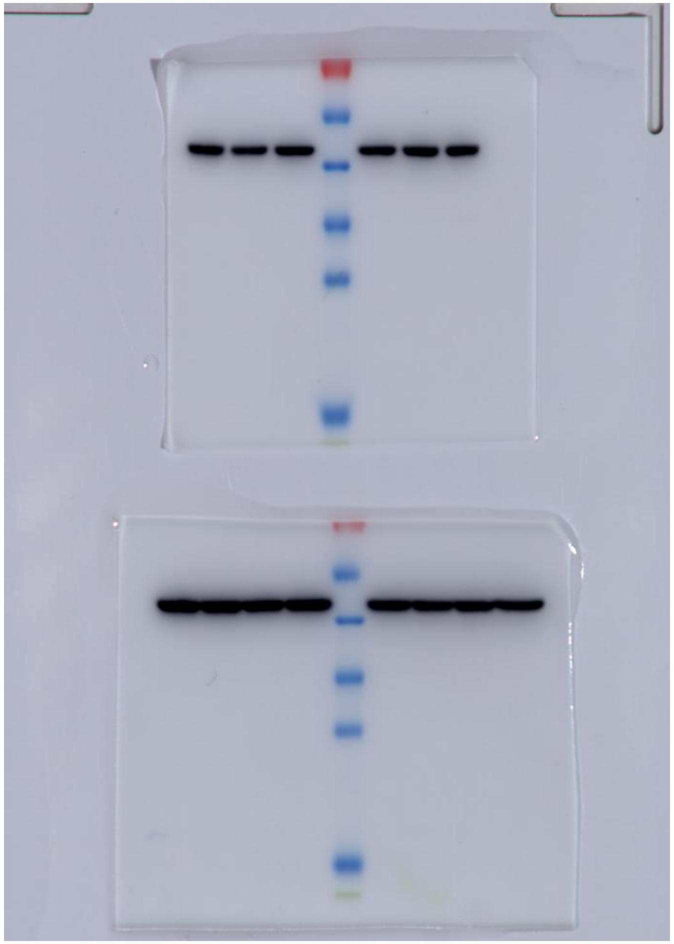

← actin 45KD

Fig5B p-AMPK 62KD

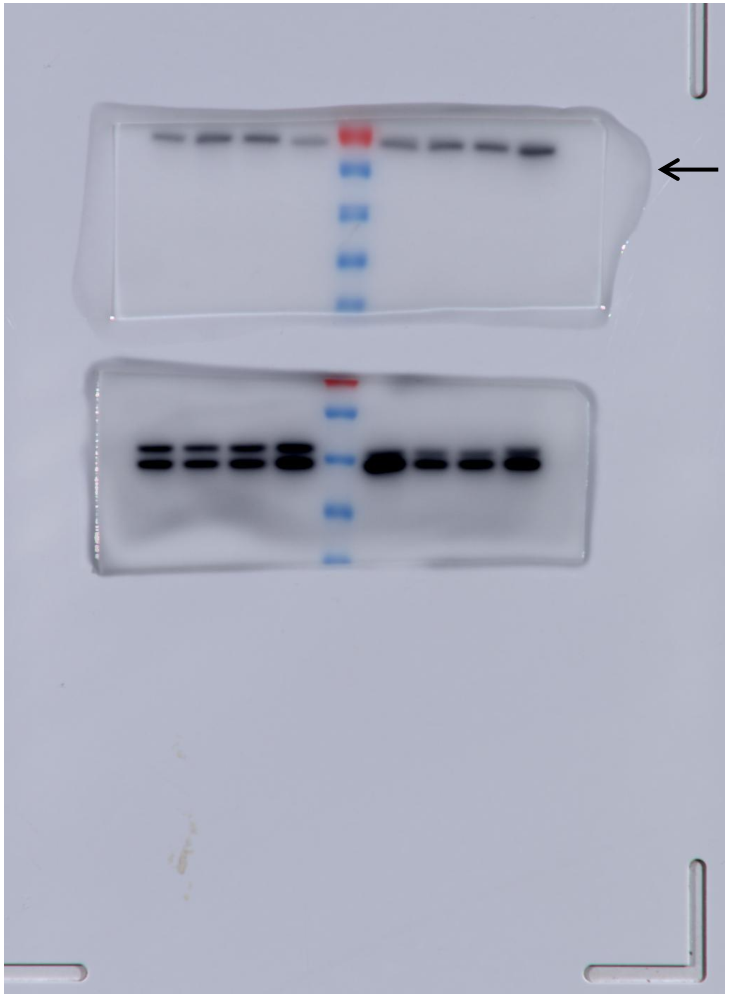

p-AMPK 62KD

Fig5B AMPK 62KD

AMPK 62KD

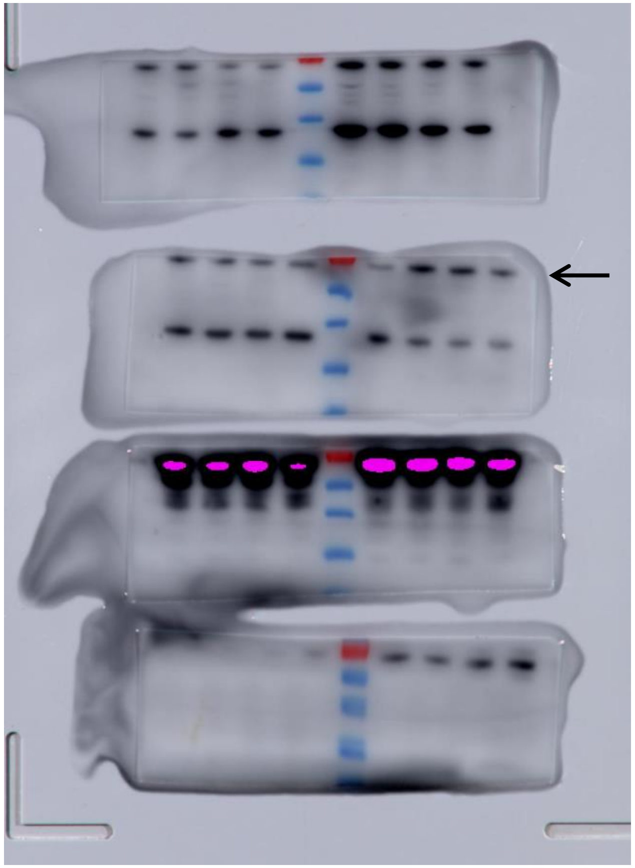

Fig5B P-AKT 473 60KD

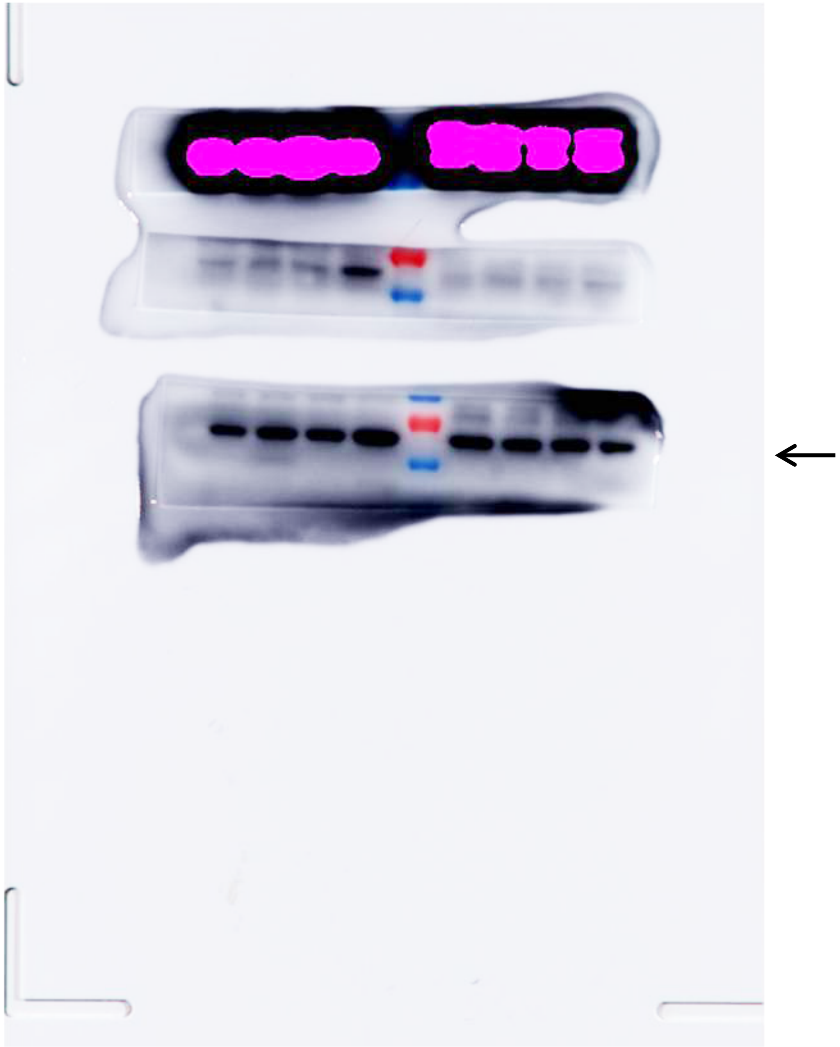

Fig5B P-AKT308 473 60KD

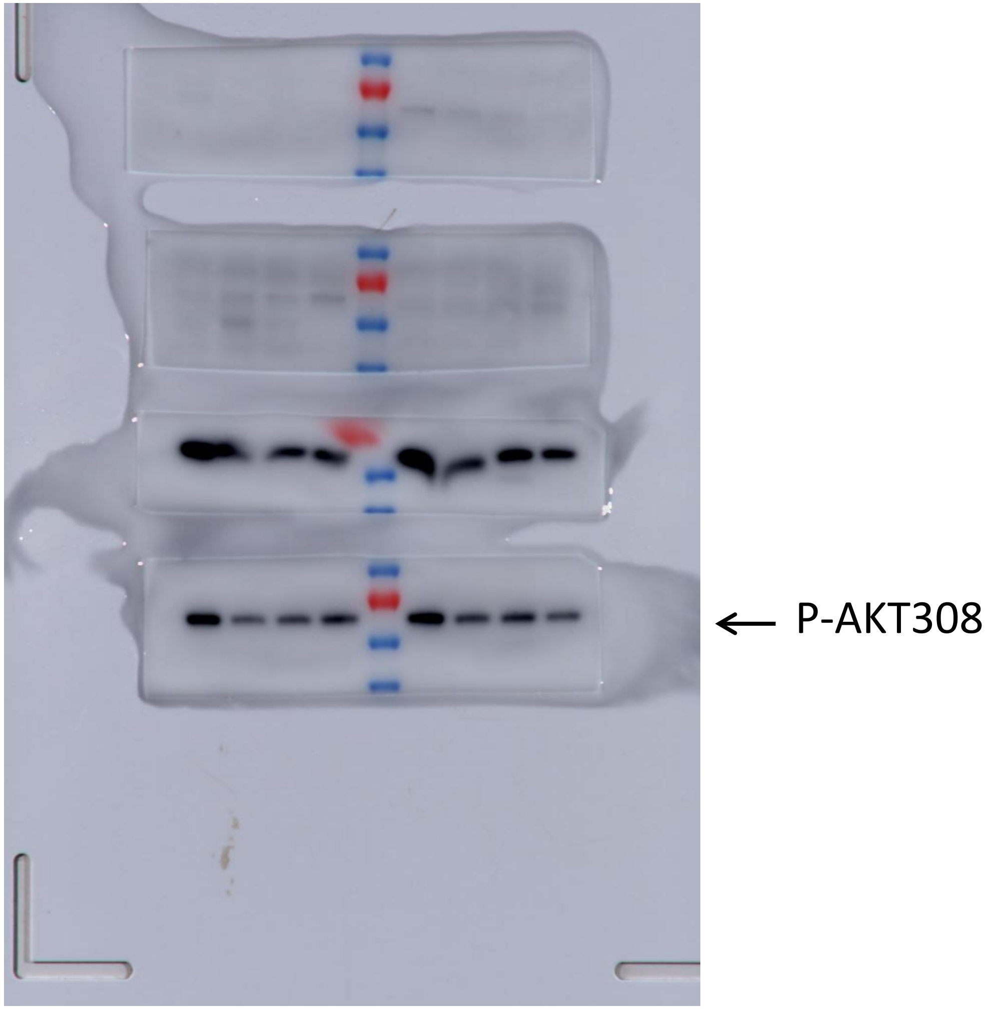

Fig5B AKT 60KD

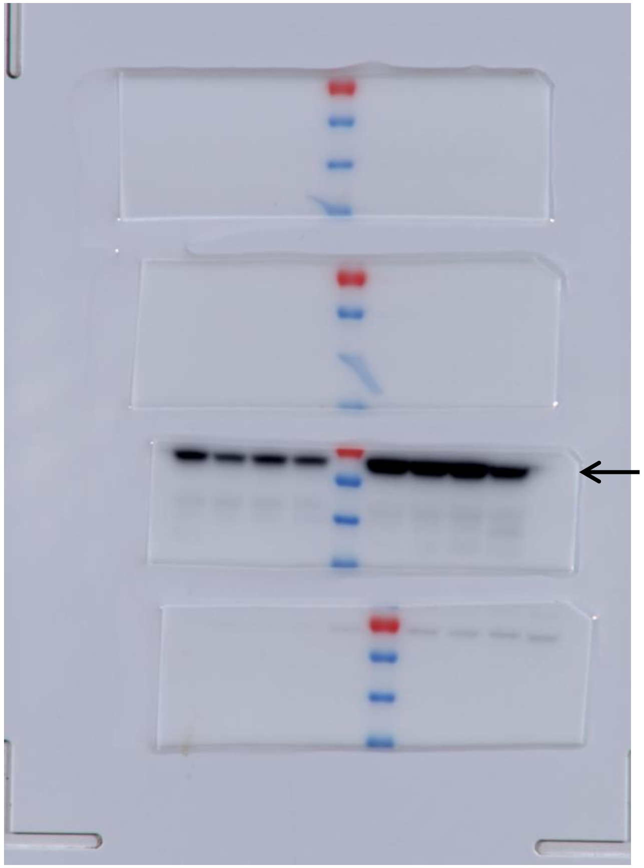

AKT 60KD

Fig5B mTOR 289KD

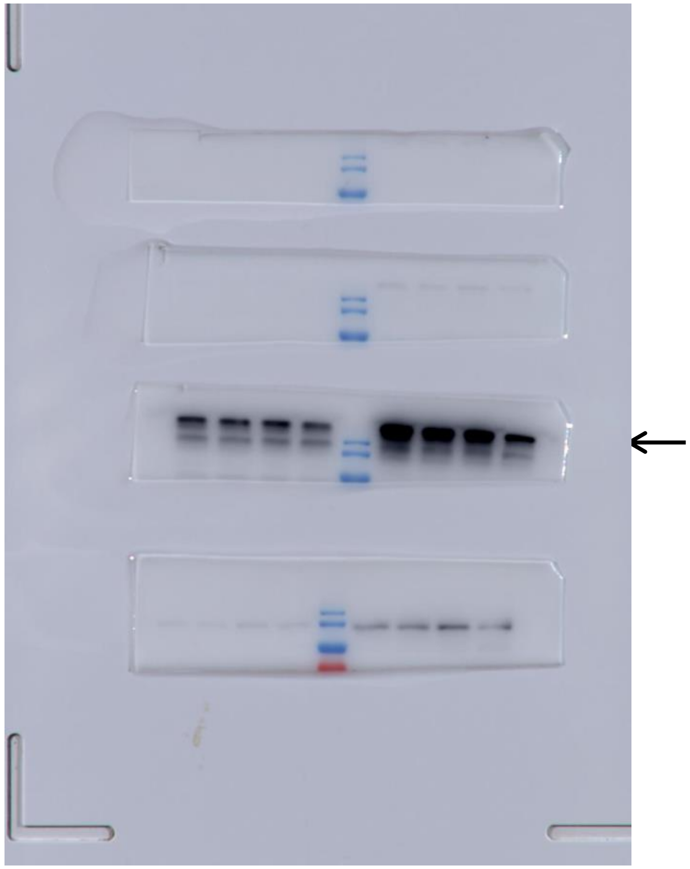

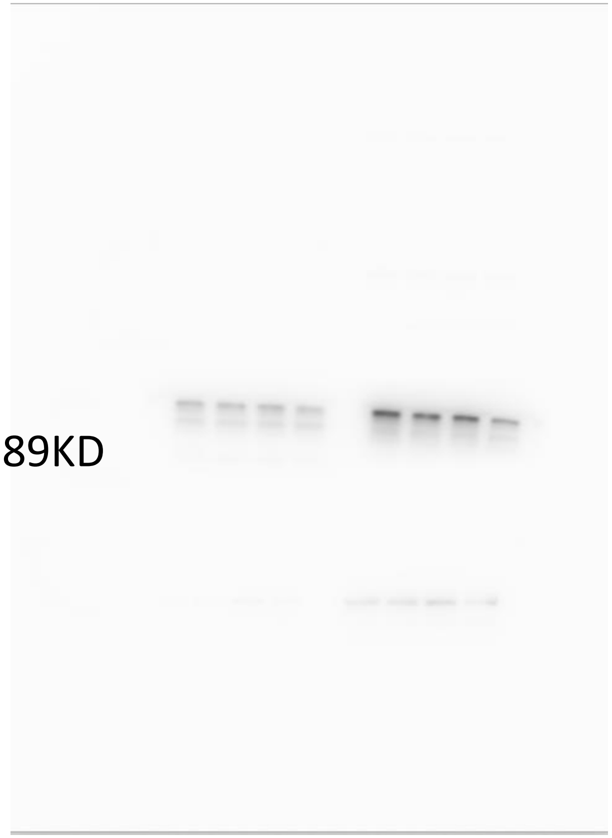

Fig5B P-MTOR2448 289KD

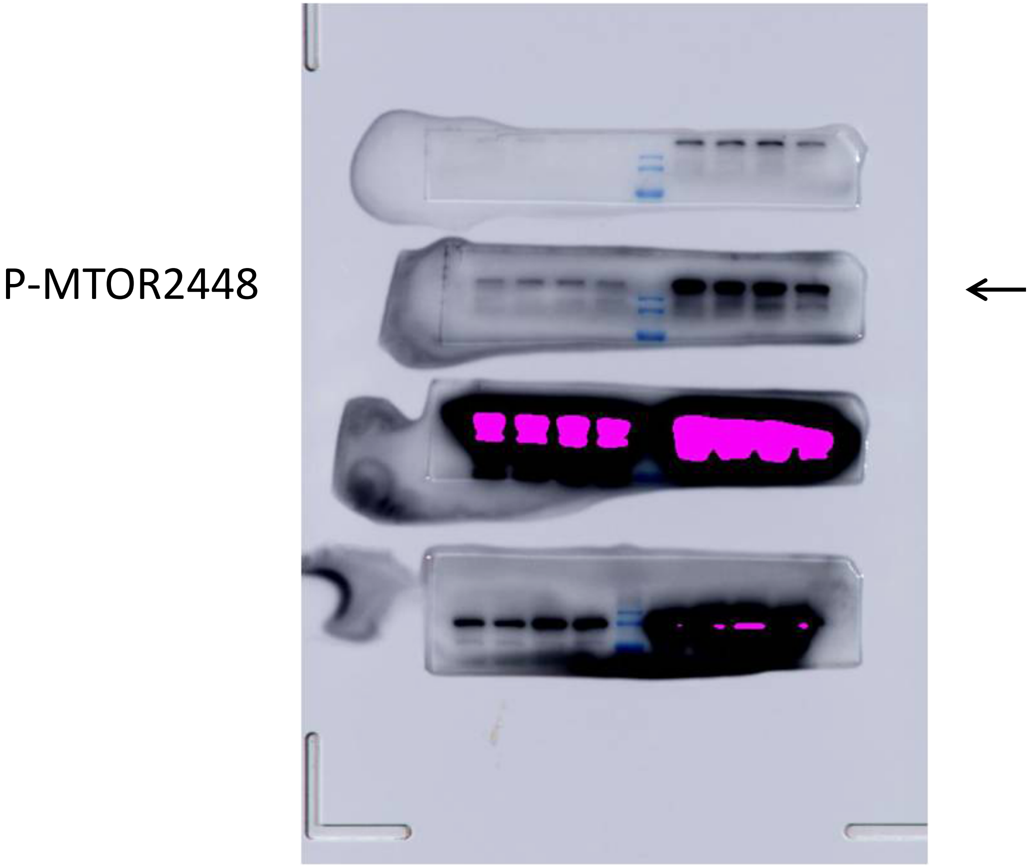

Fig5B P-MTOR2481 289KD

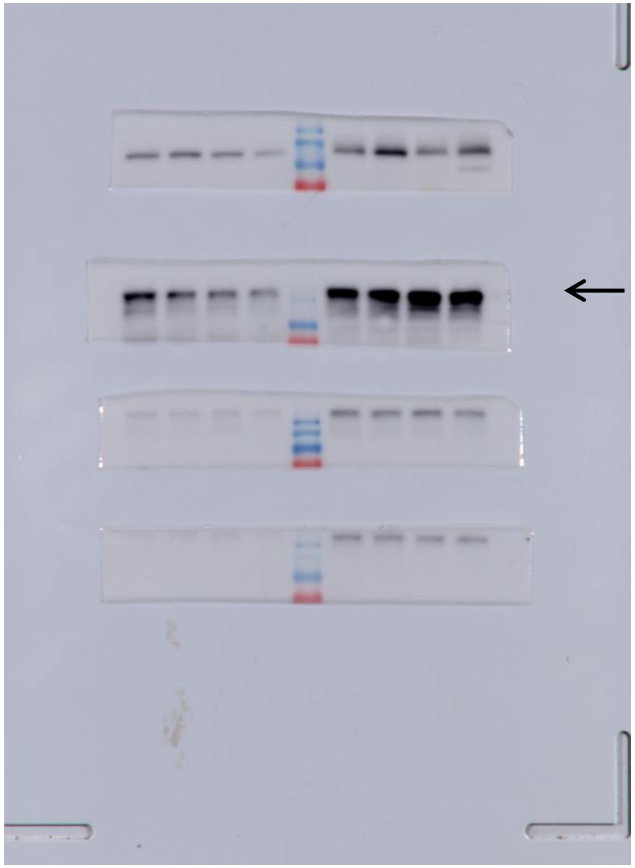

P-MTOR2481

Fig5B p-P70S6K 70KD

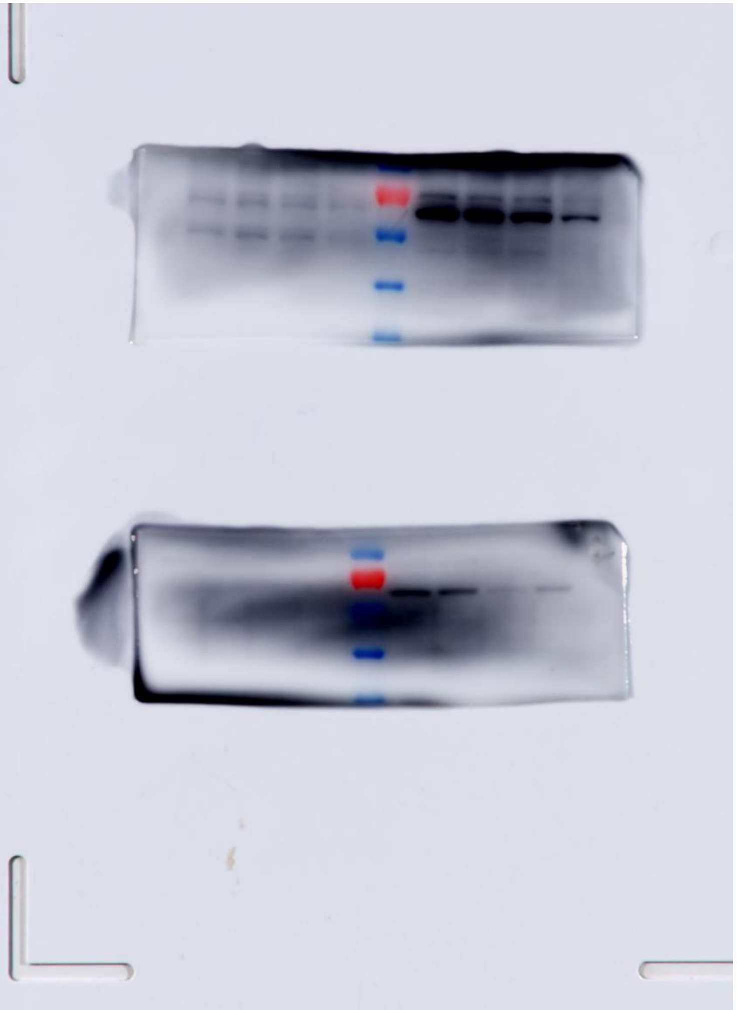

Fig5B P70S6K 70KD

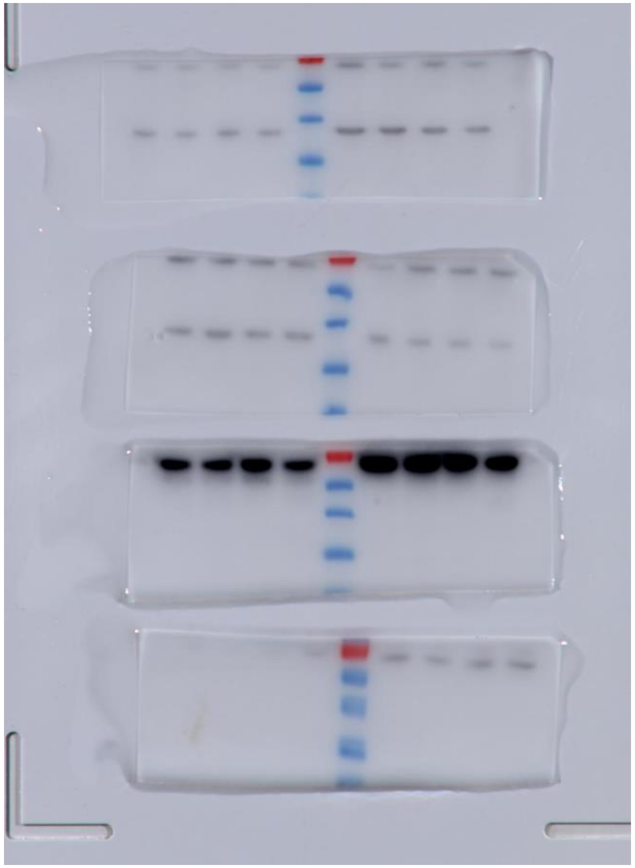

Fig5B actin 45KD

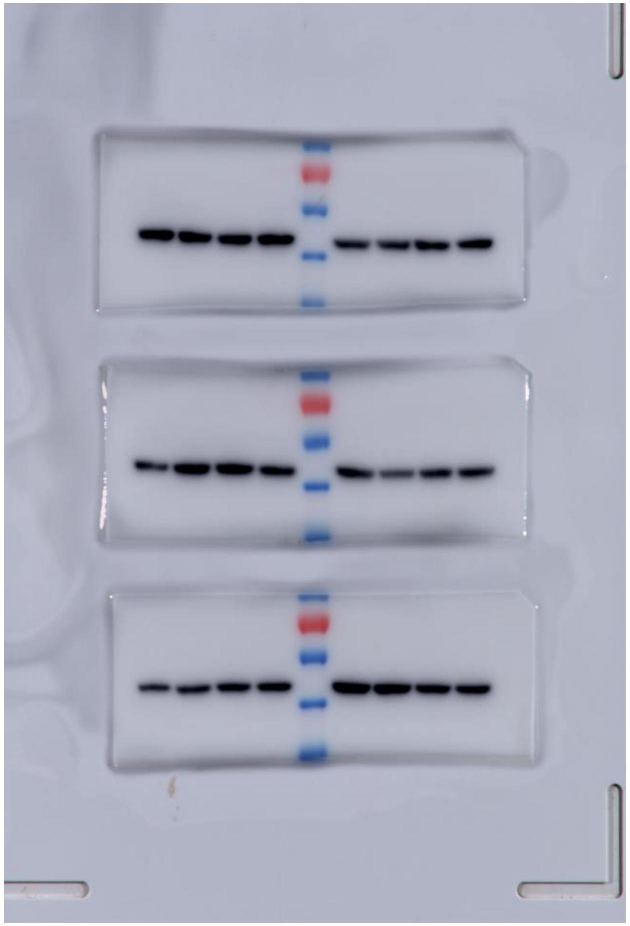

←

Fig5B P-ERK 44,42KD

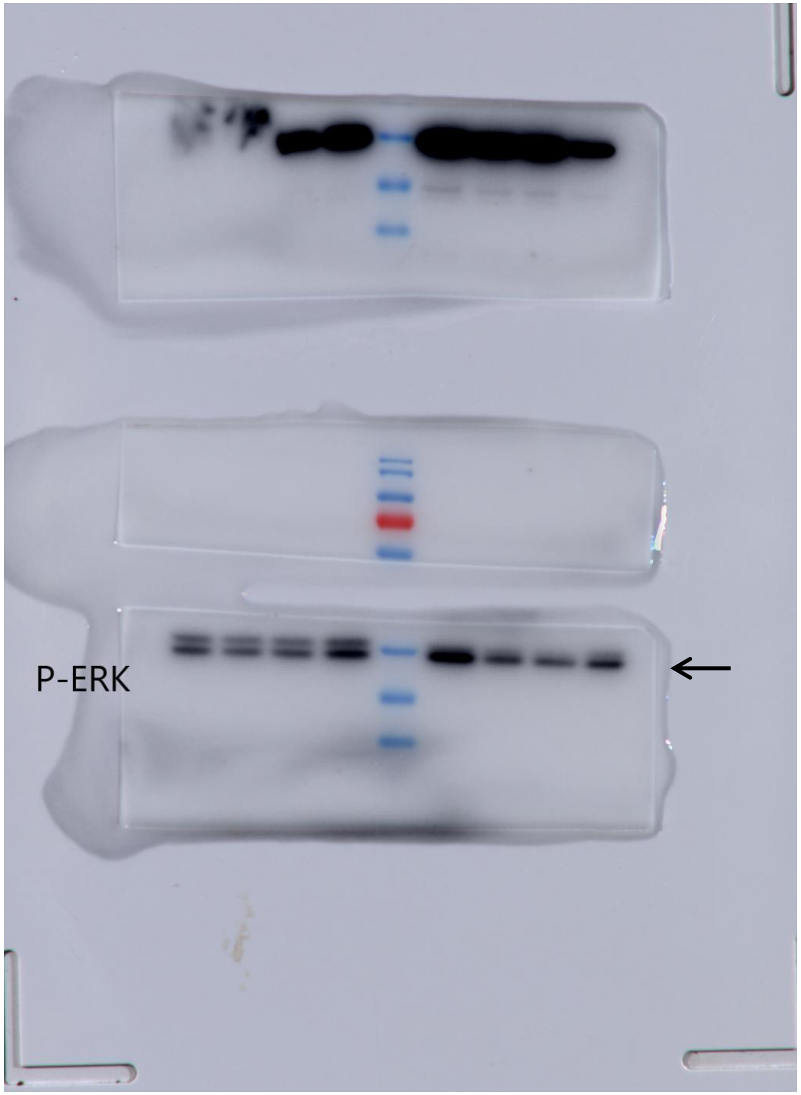

Fig5B P-P38 40KD

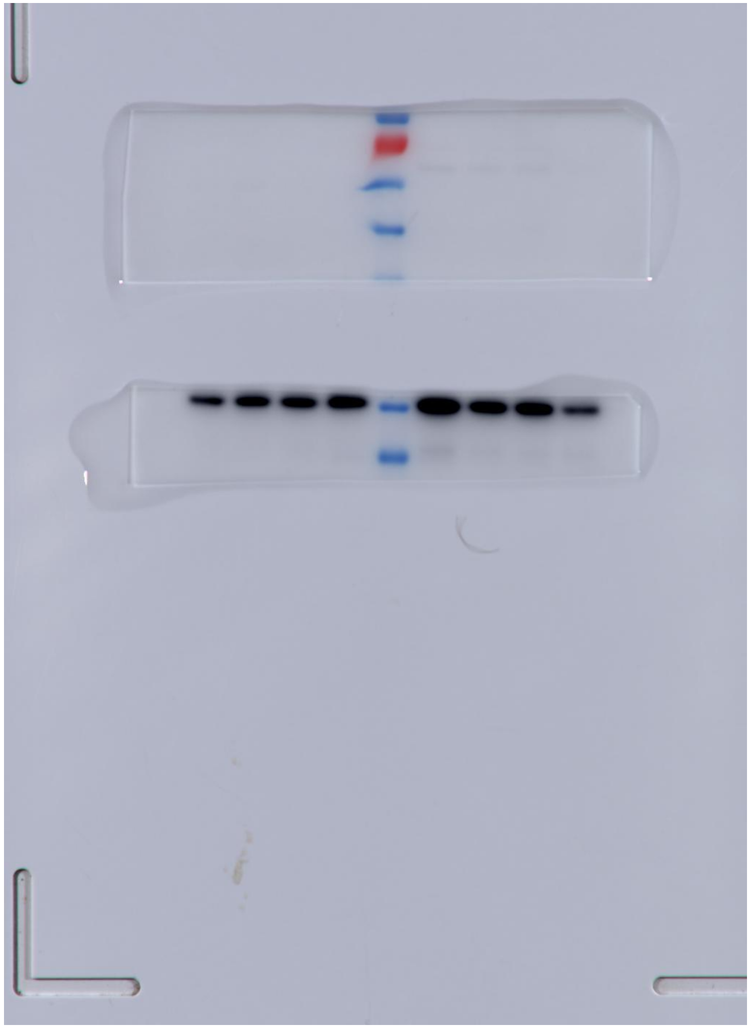

← P-P38 40KD

Fig5B P-JNK54,46KD JNK54,46KD

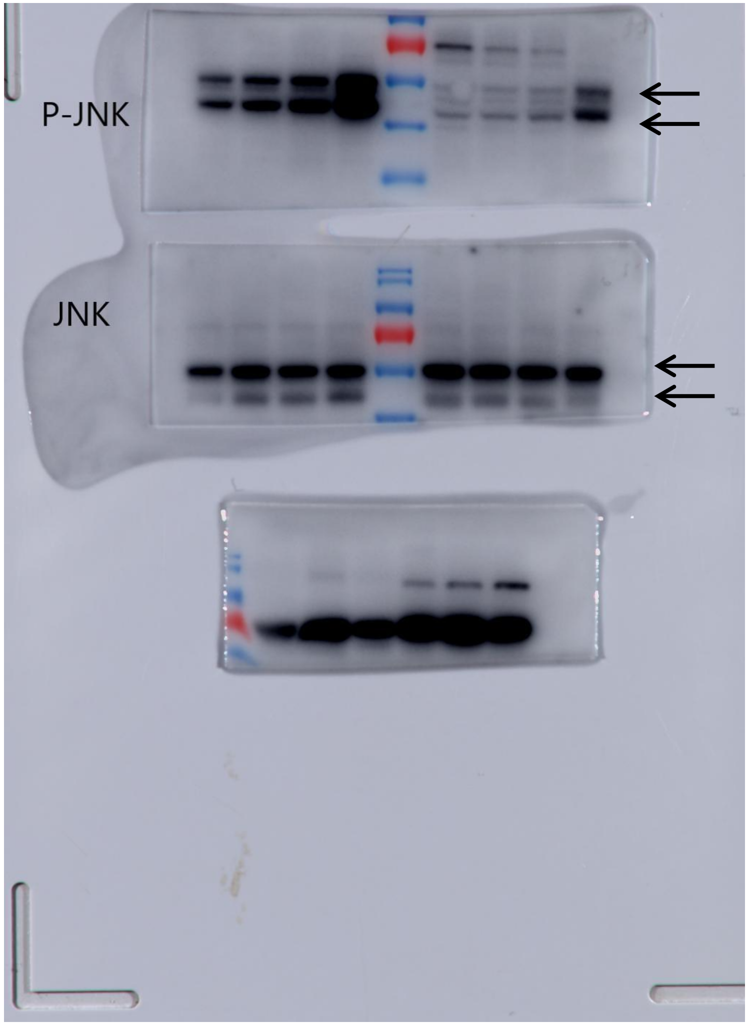

Fig5B actin 45KD

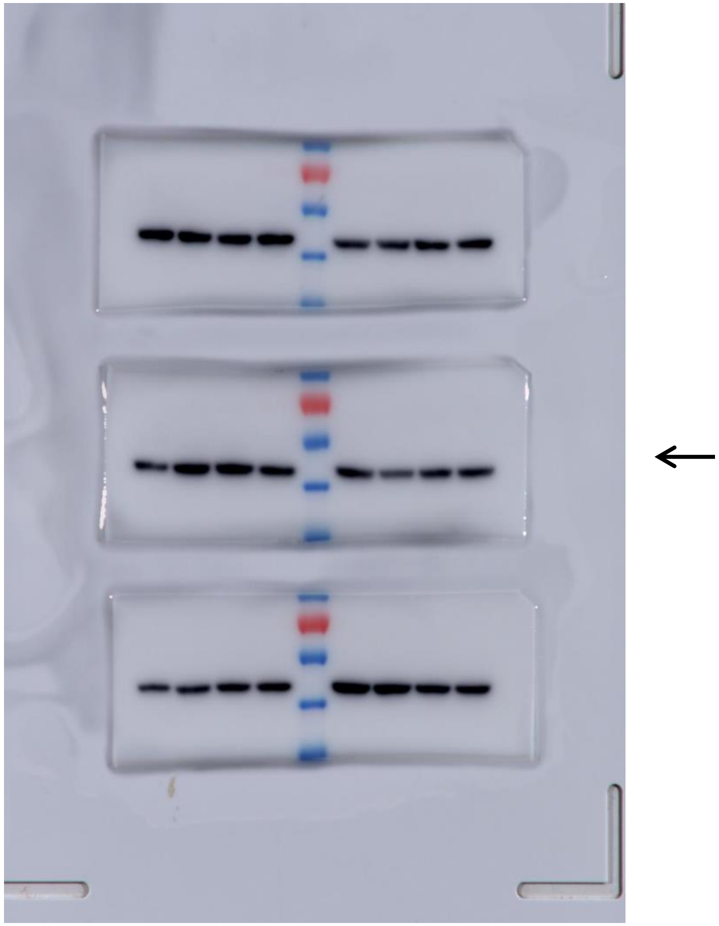

Fig6B MCL-1 48KD

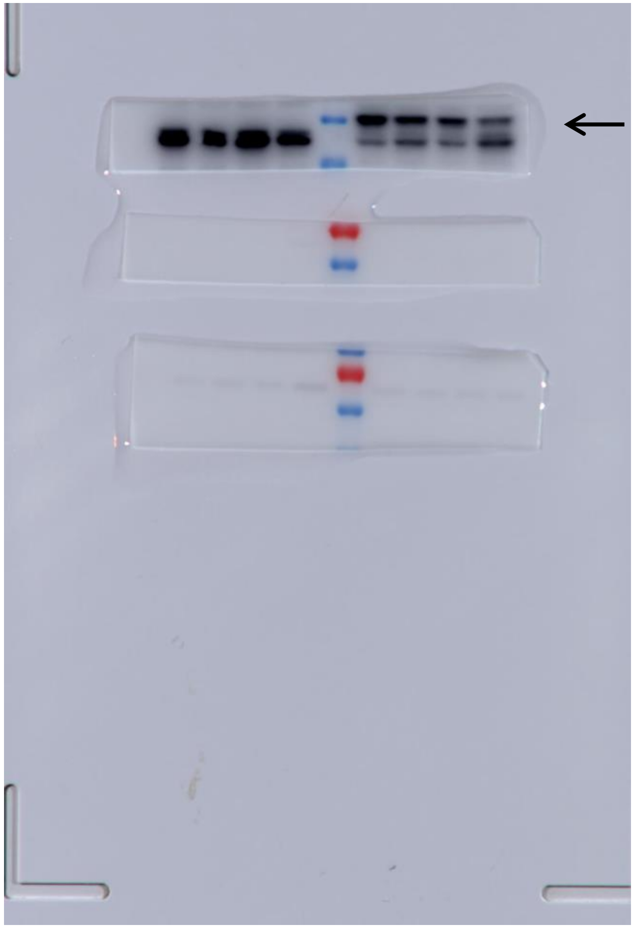

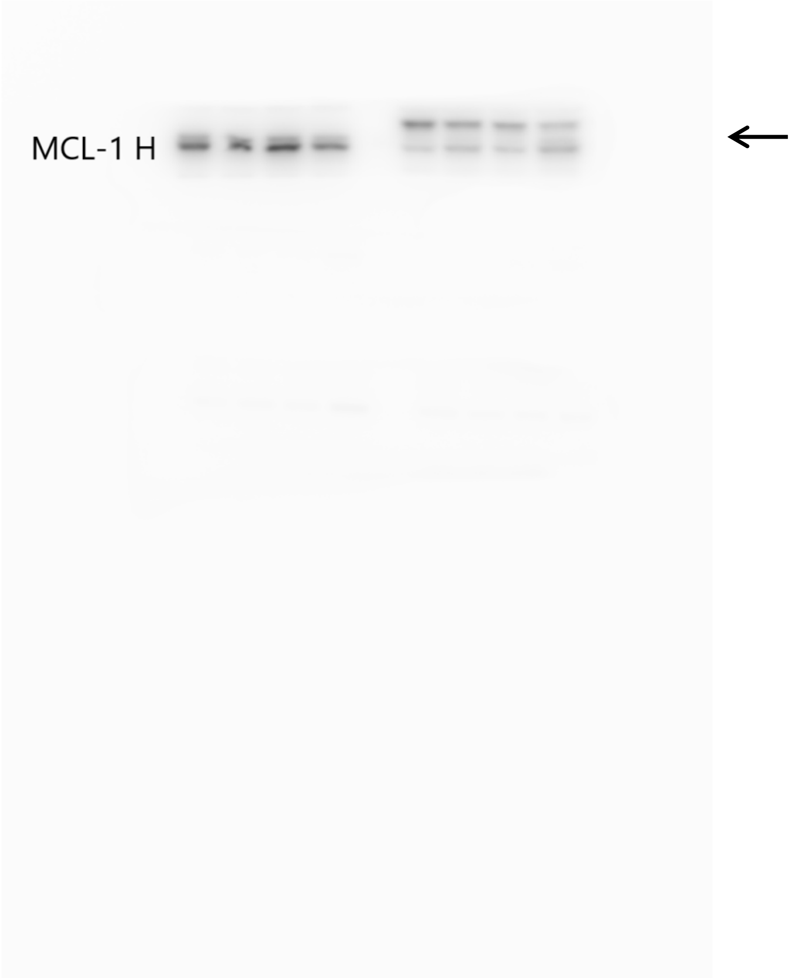

Fig6B BCL2 26KD

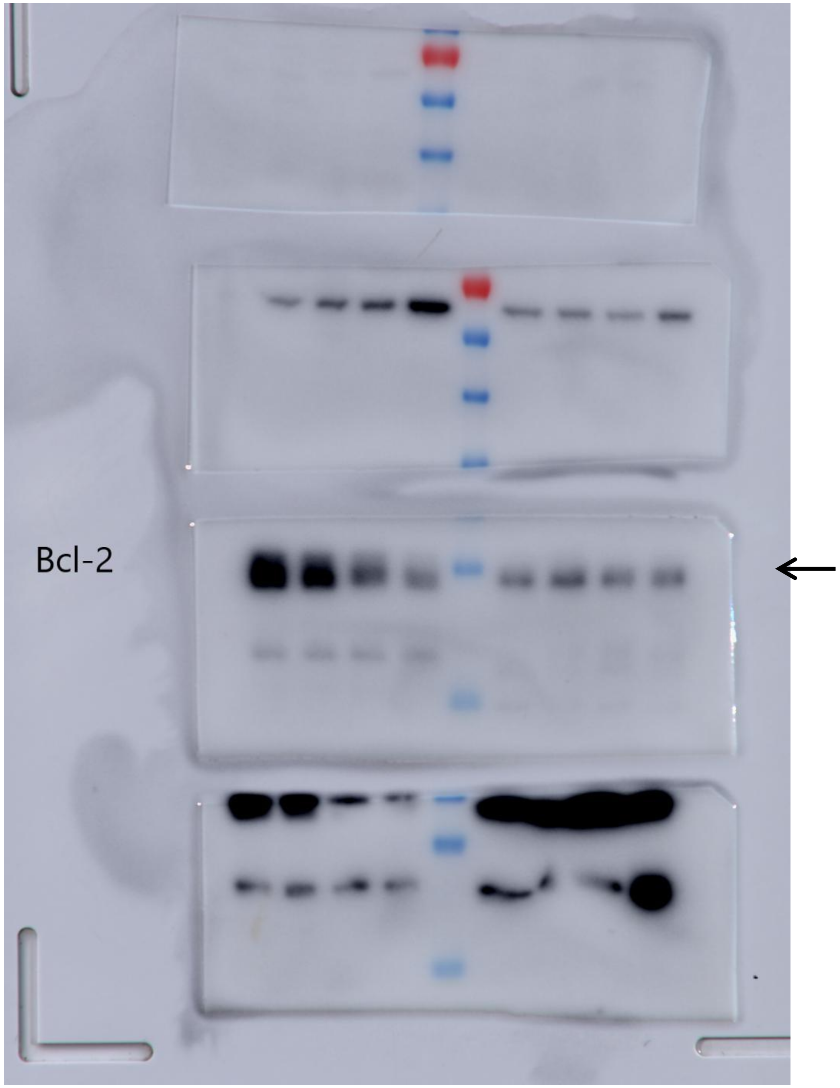

Fig6B BCL-XL 30KD

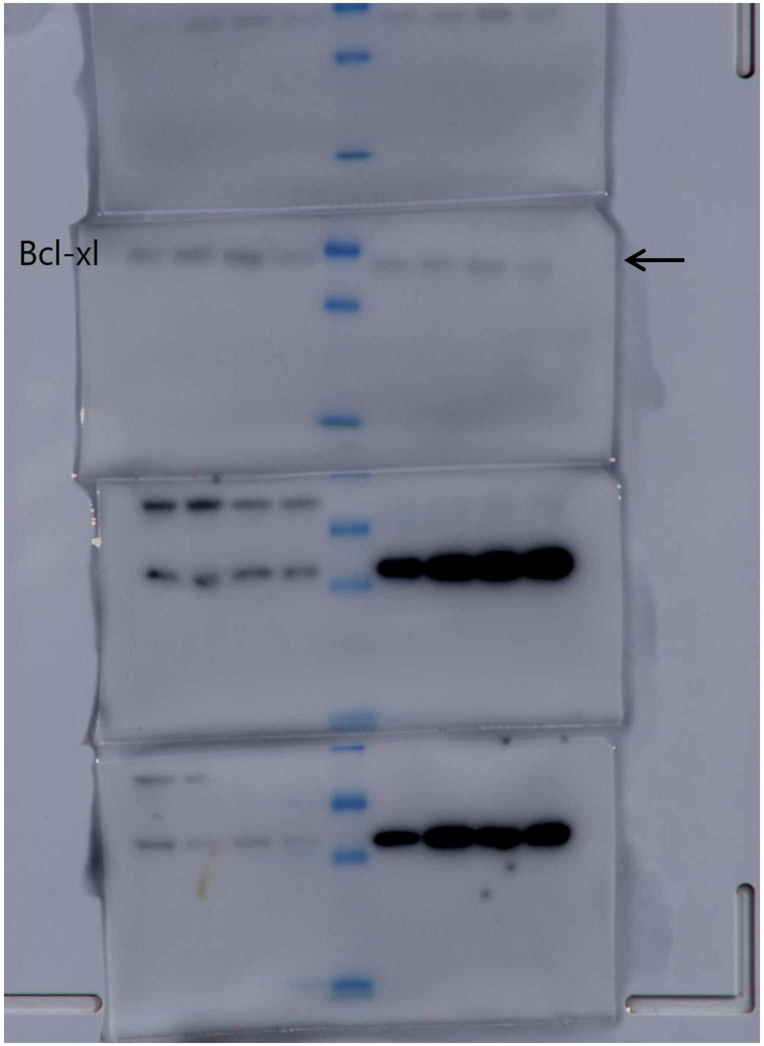

Fig6C actin 45KD

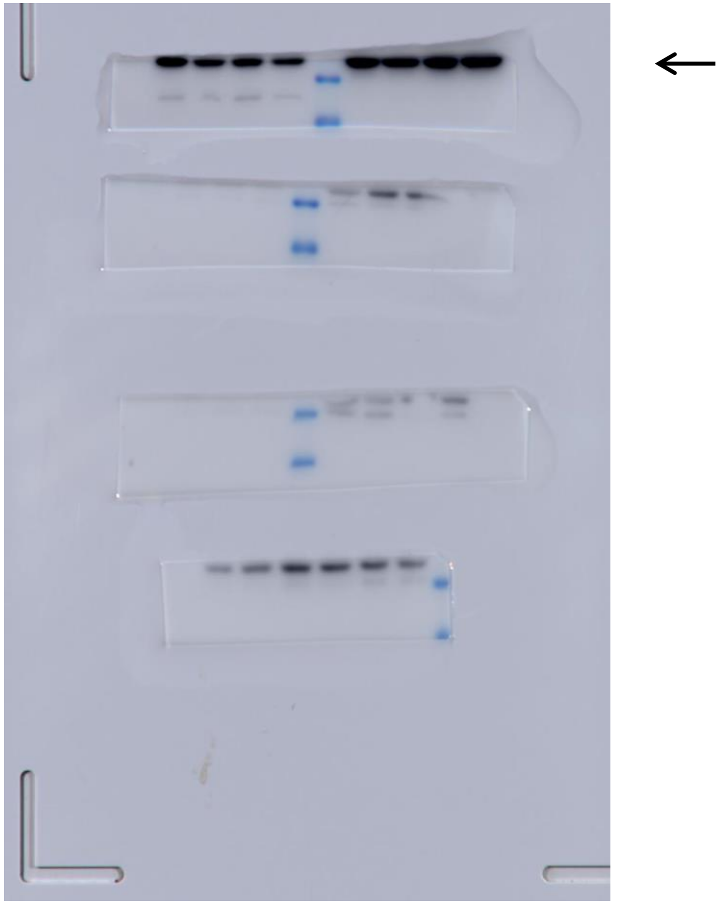

Fig6B C-IAP-1 62 KD H H+L

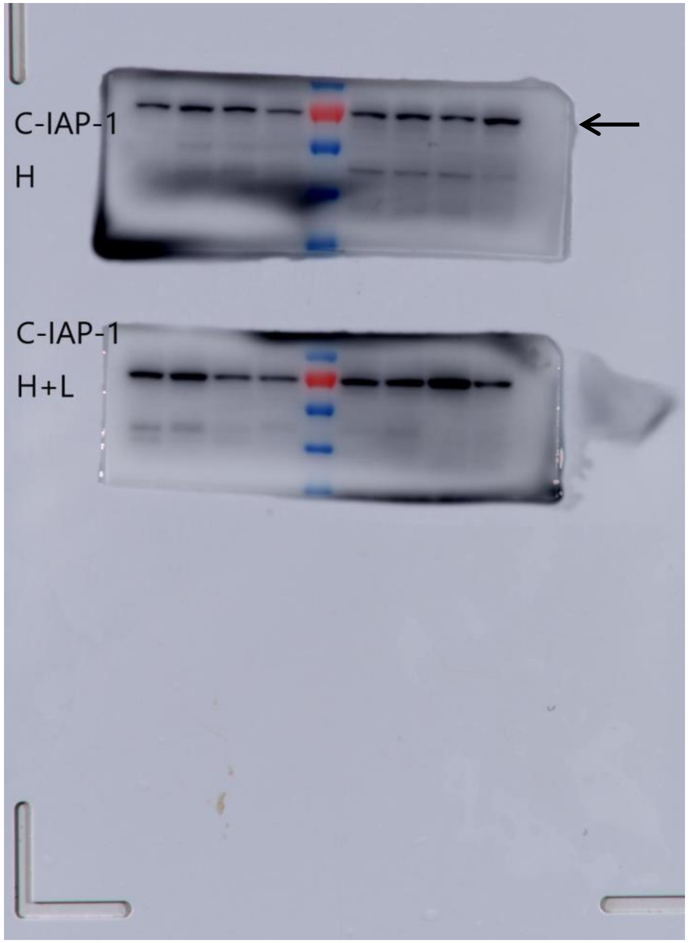

Fig6B CIAP-2 70KD

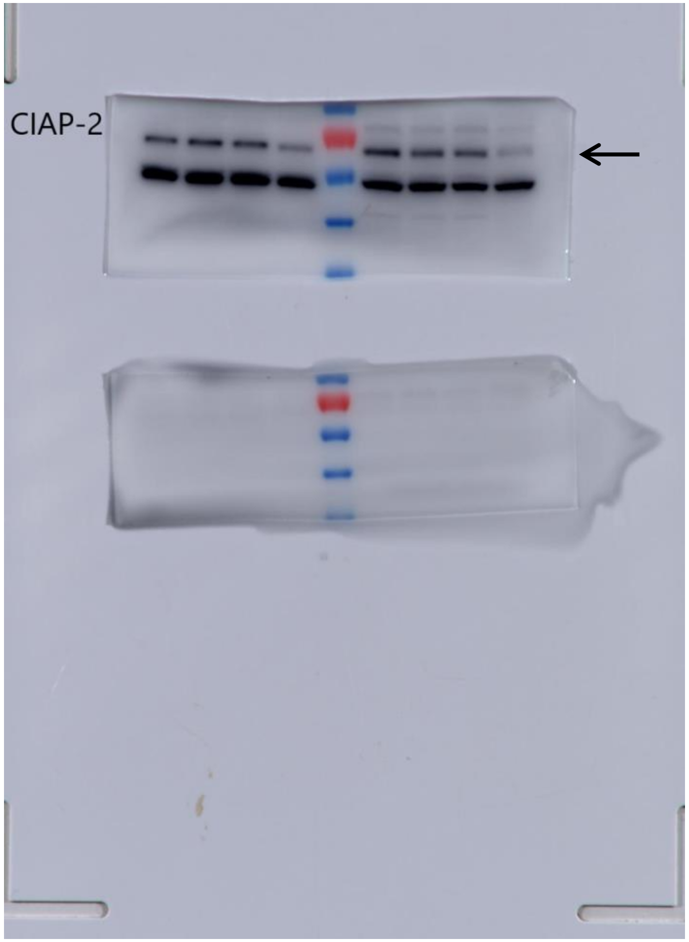

Fig6B XIAP 53KD

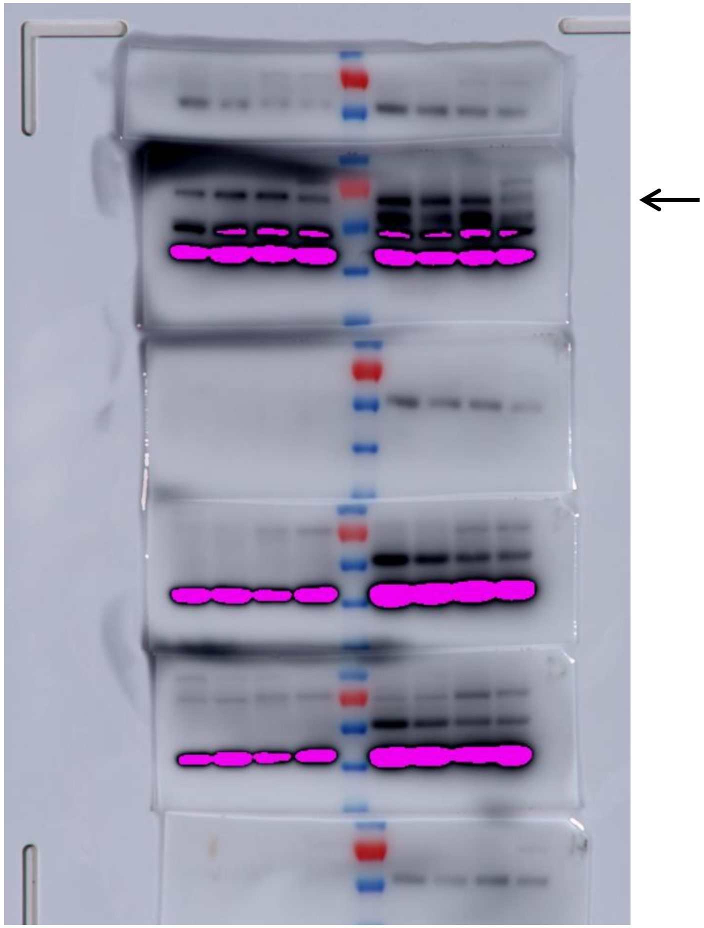

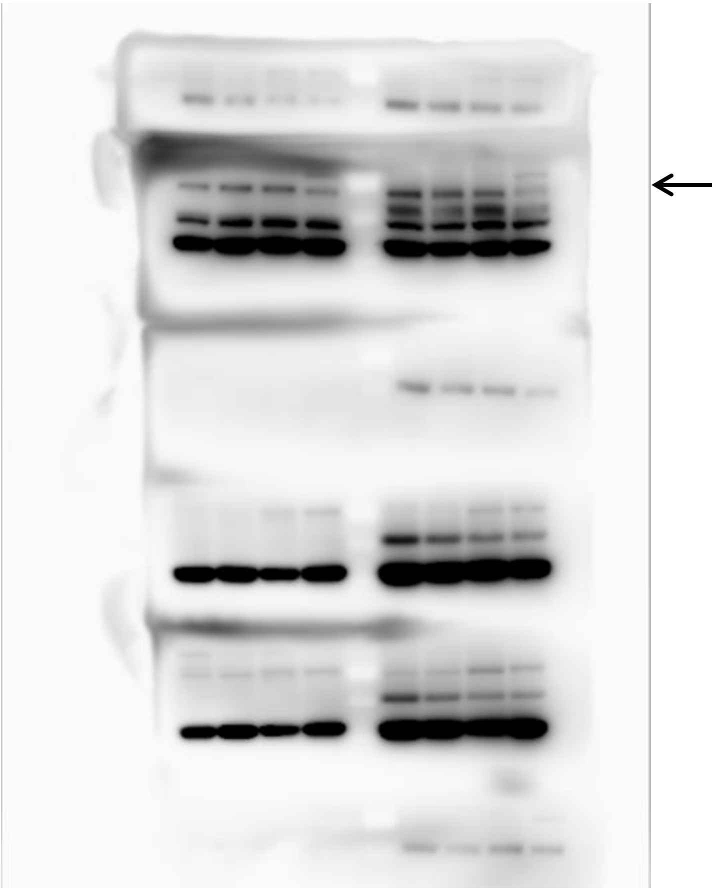

Fig6B actin 45KD

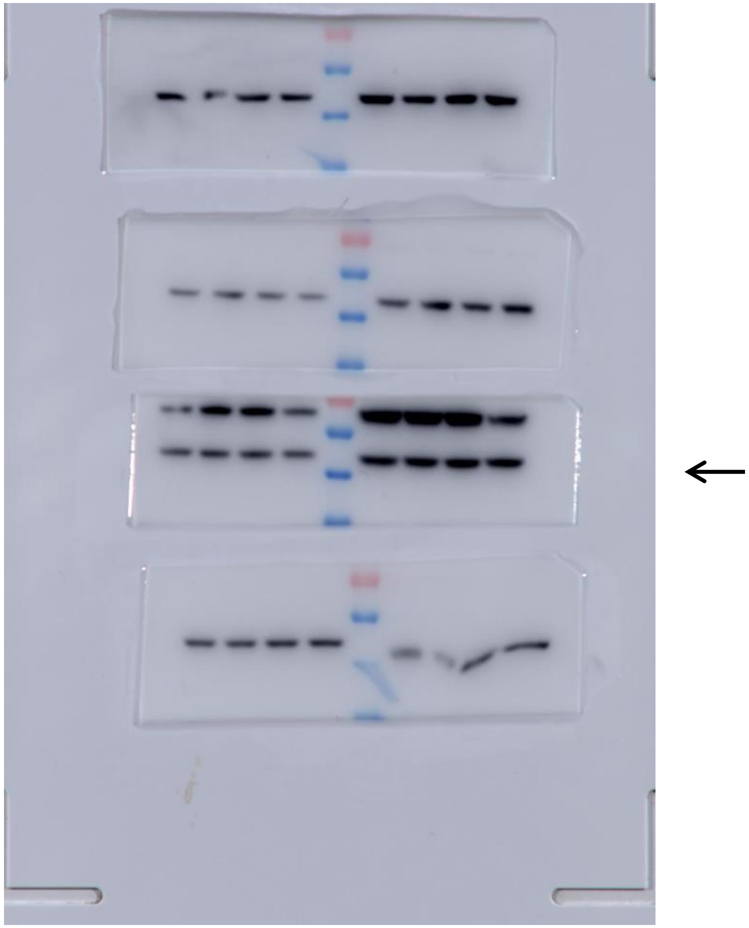

Supplementary 4B Bim 23,12KD

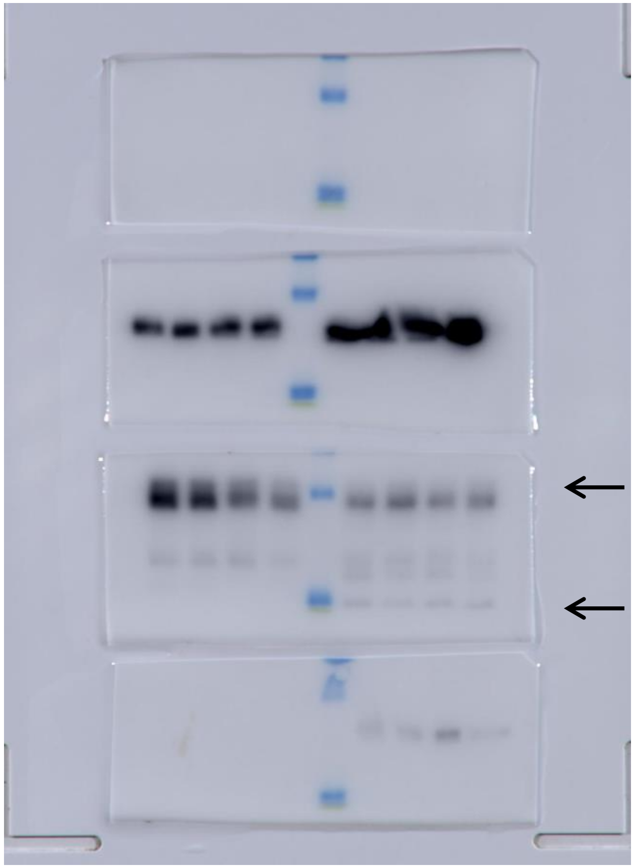

Bim 23,
12KD

Supplementary 4B BAX 20KD

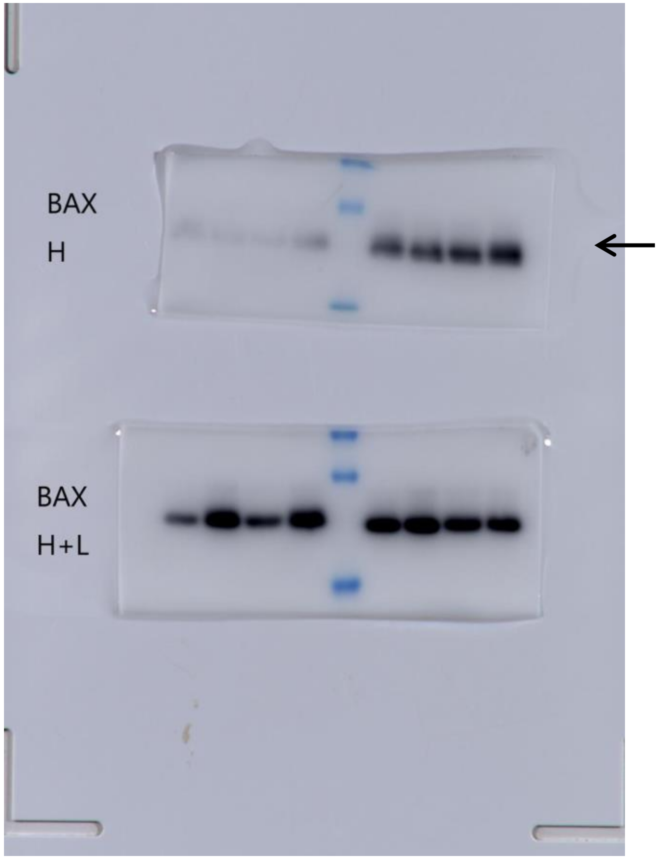

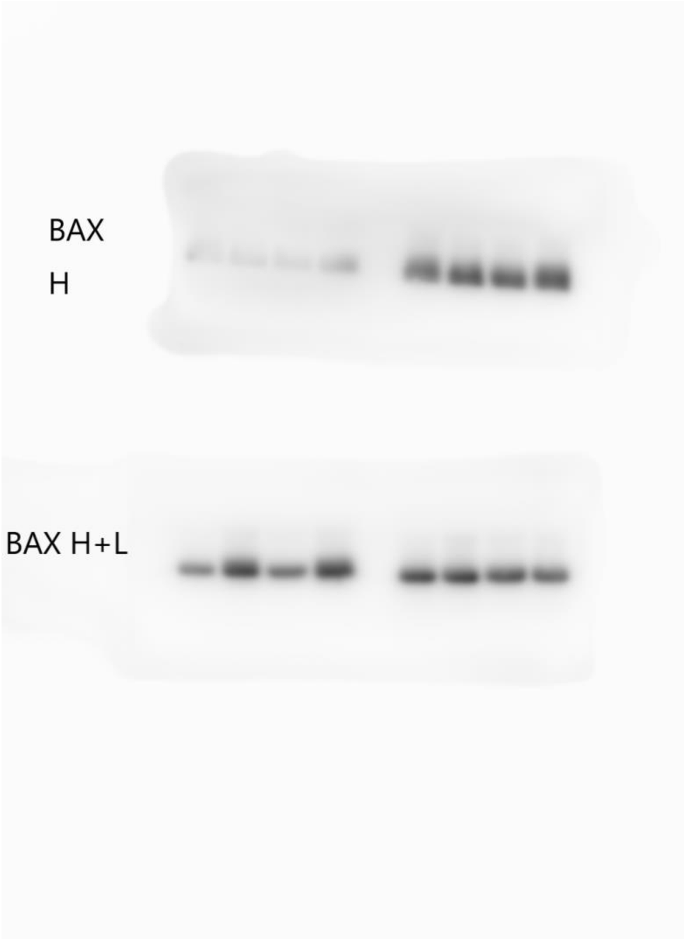

Supplementary 4B BAK25KD

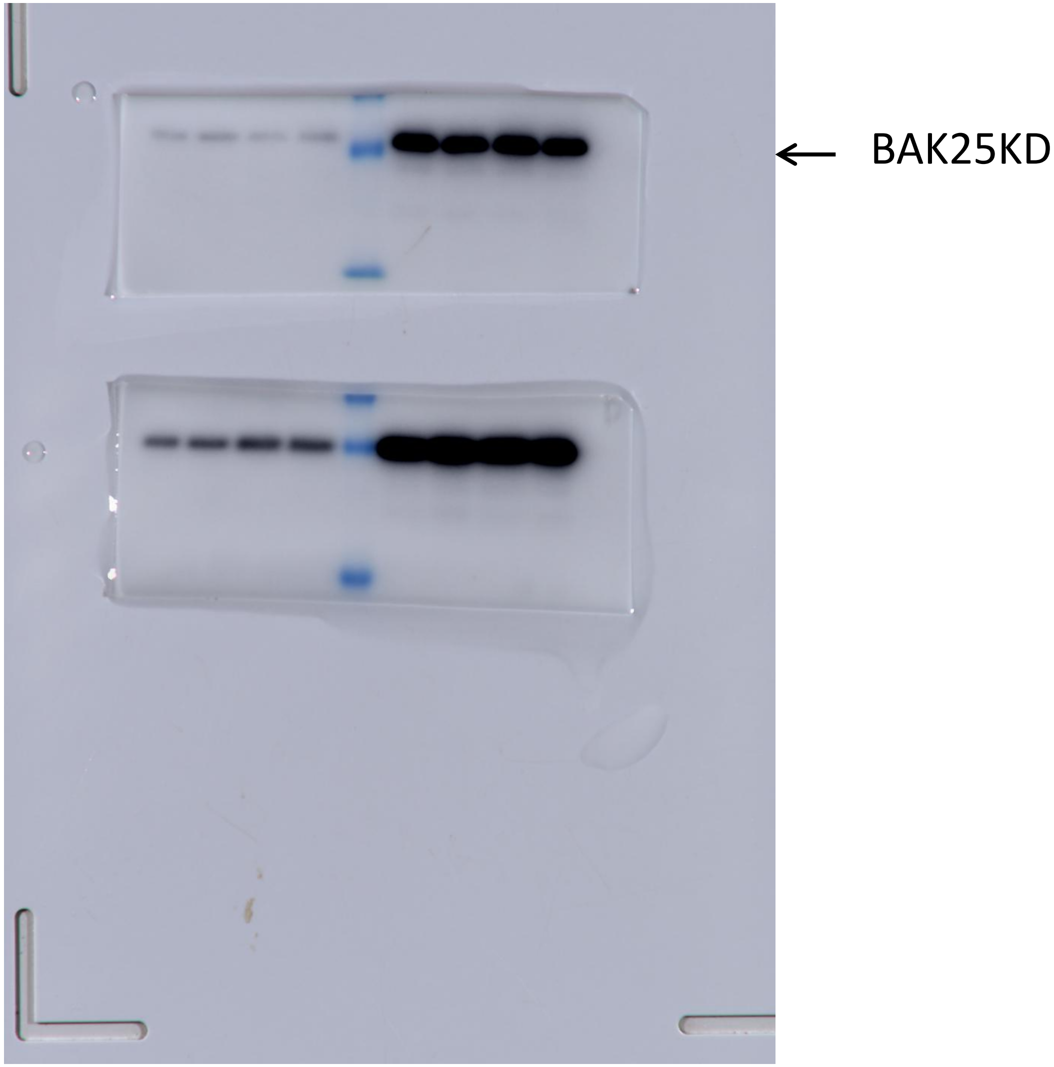

Supplementary 4B Bid 22KD

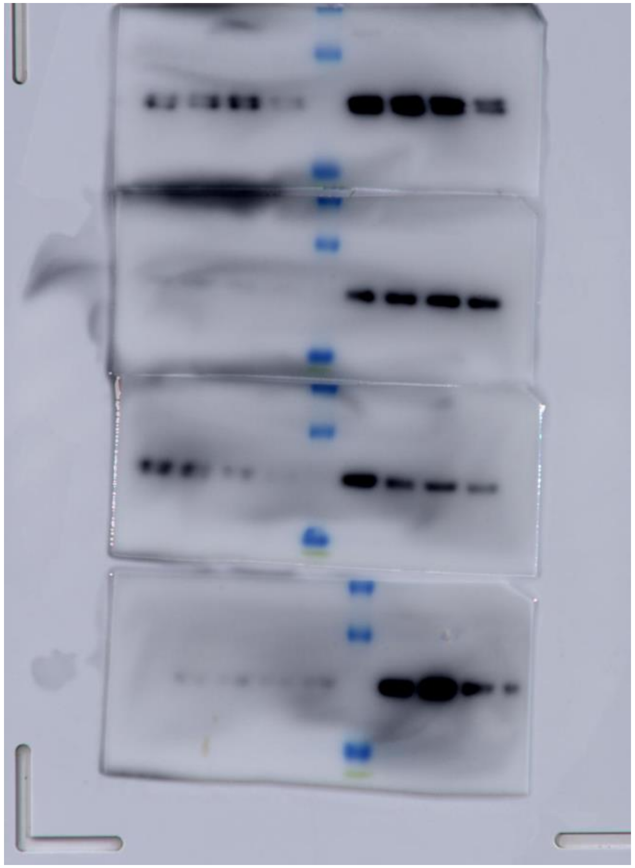

← Bid 22KD

Supplementary 4B Bad23KD

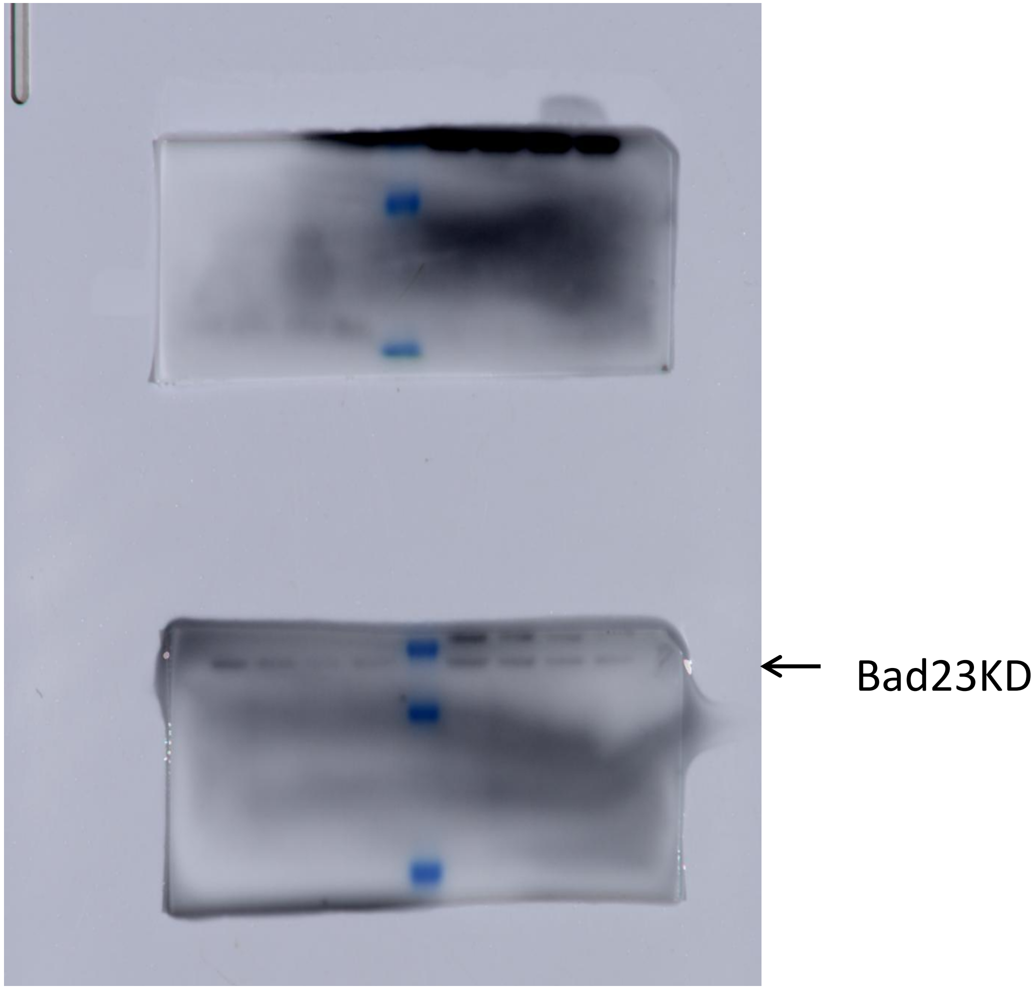

← Bad23KD

Supplementary 4B actin 45KD

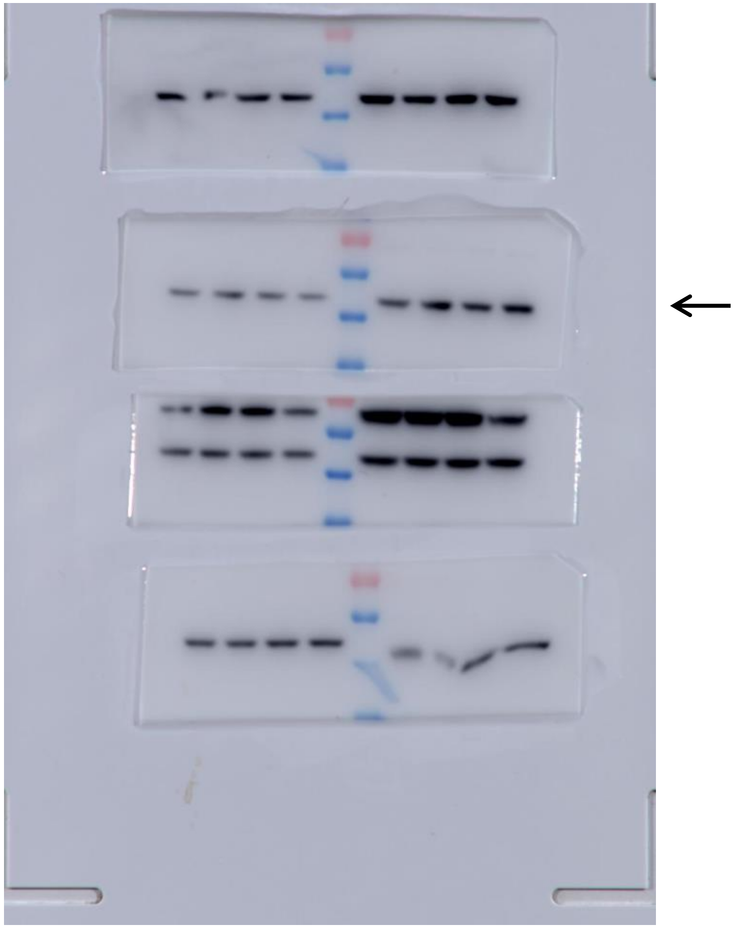

Hyperthermia+Loaplatin (H+L)

Fig4D PARP 116, 89KD

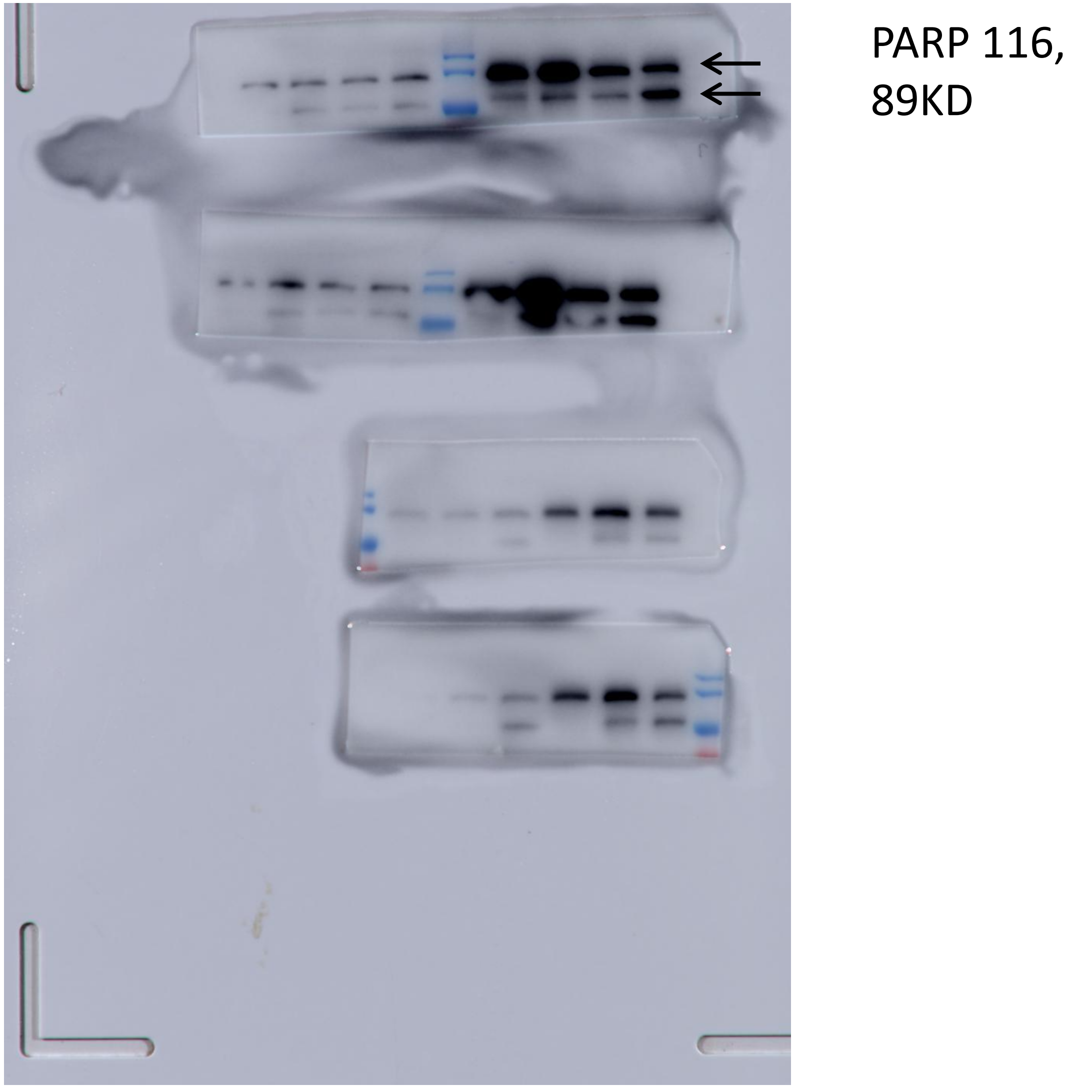

Fig4D Caspase 3 35,19,17KD

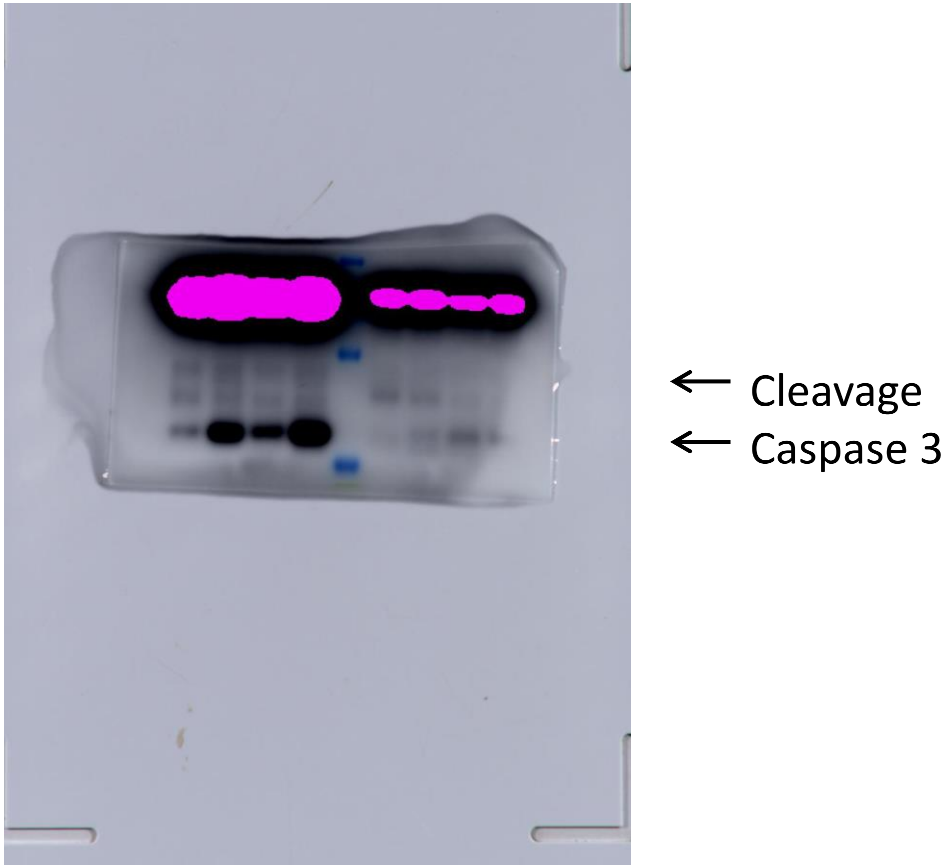

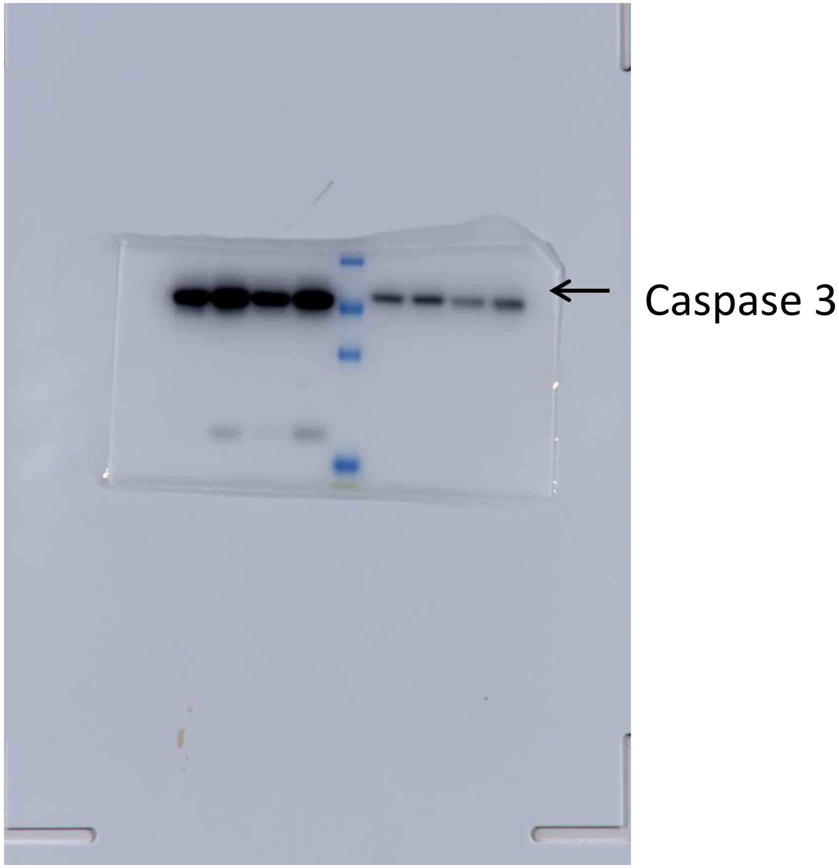

Fig4D Actin 45KD

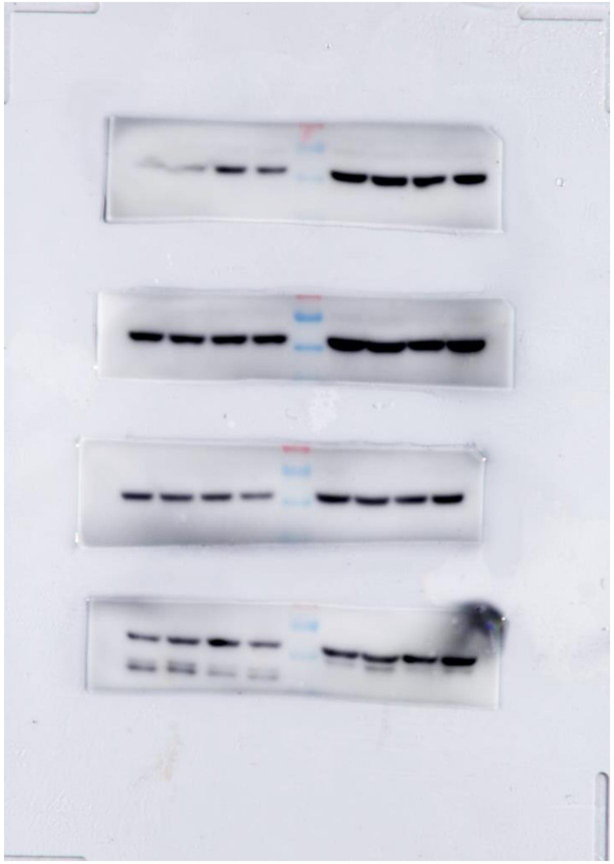

←

Fig4D P62 62KD

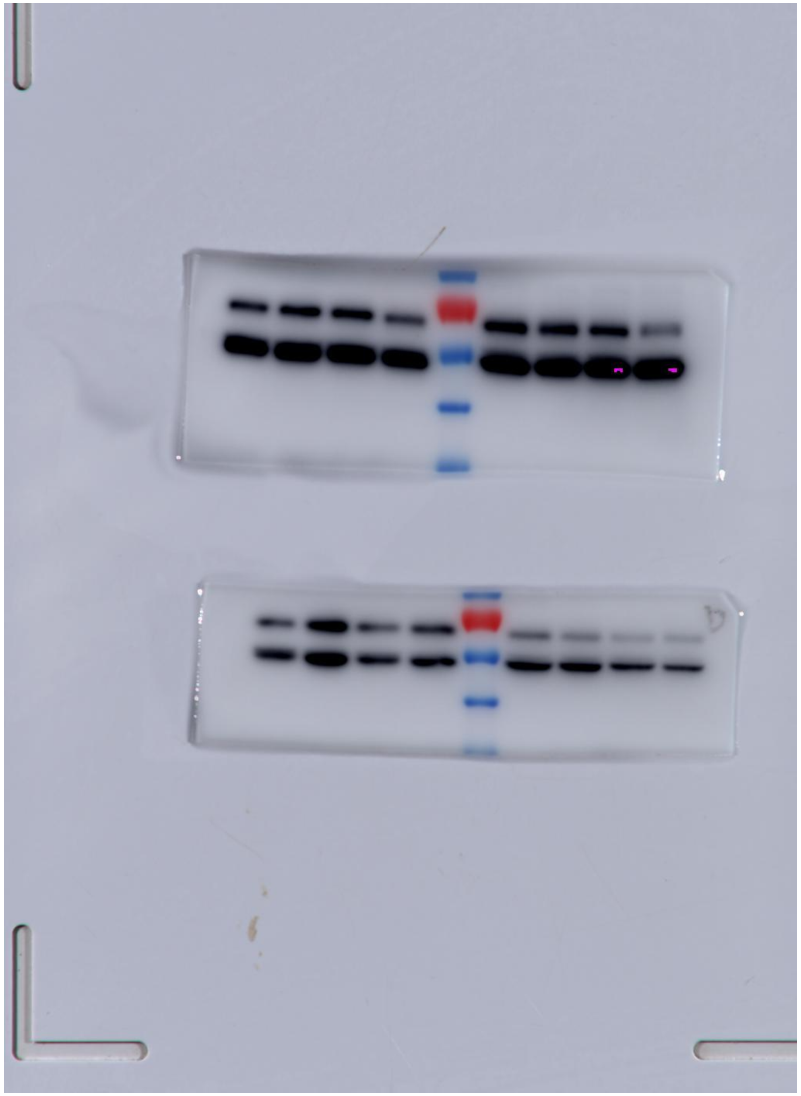

← P62 62KD

Fig4D LC3 16, 14KD

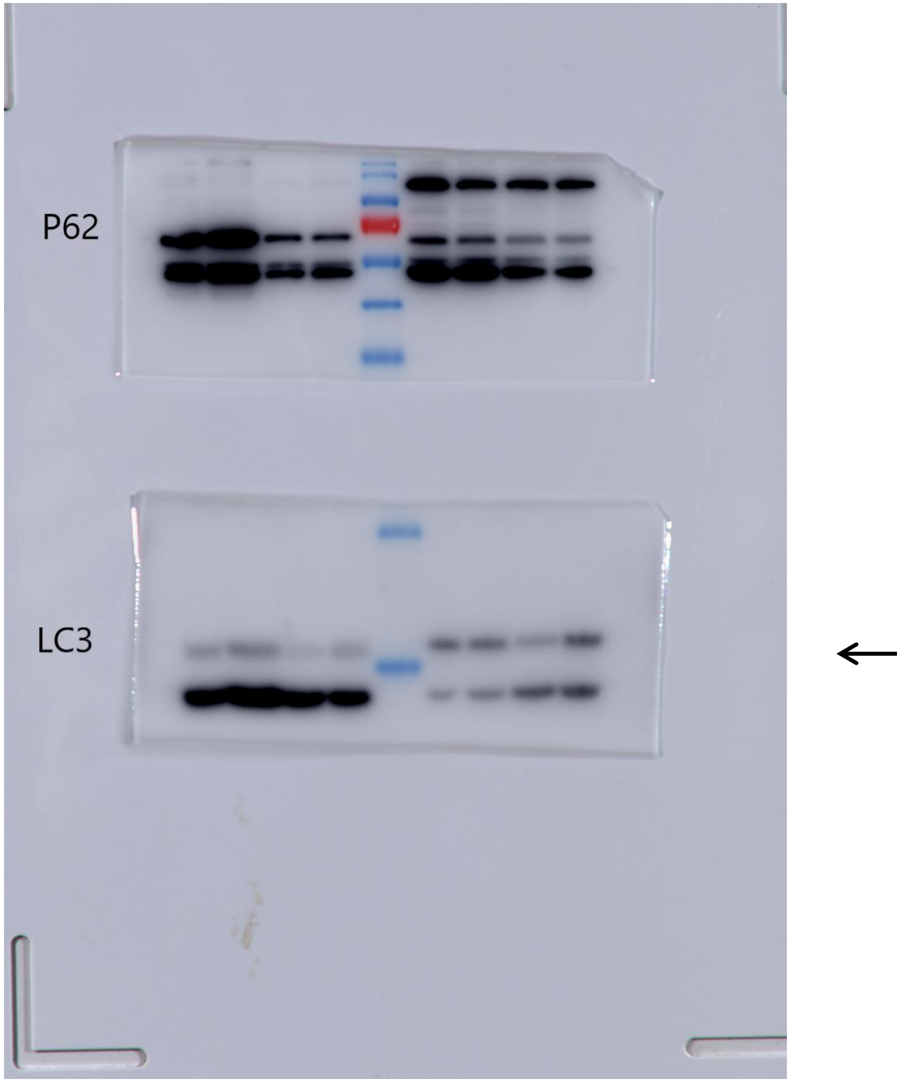

Fig4D Actin 45KD

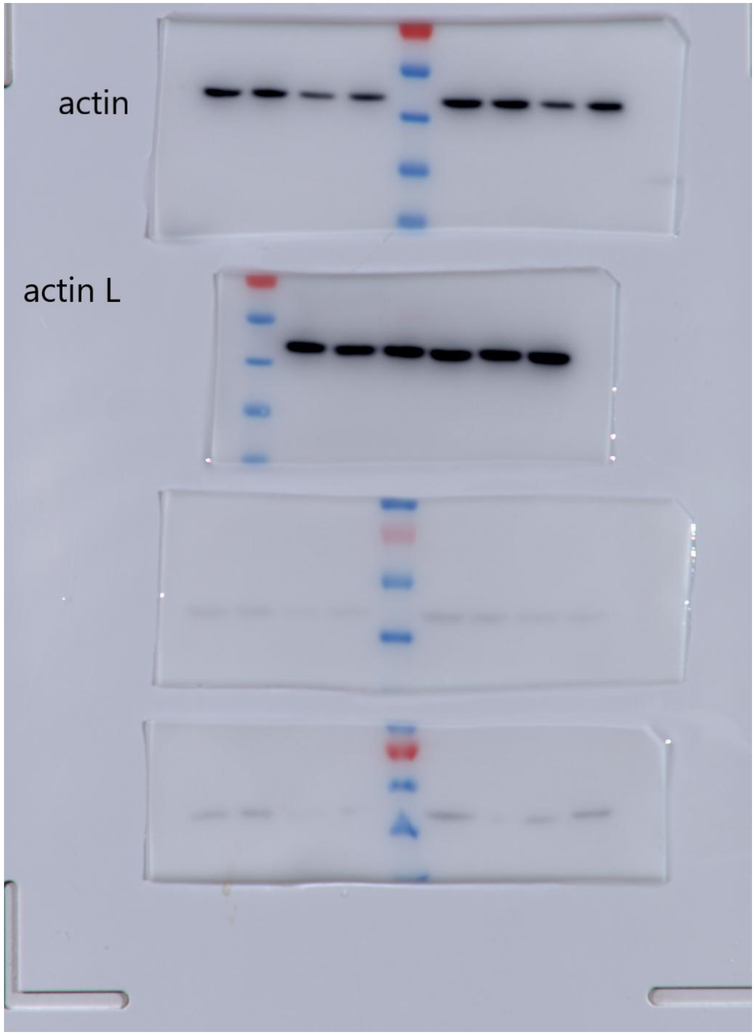

Fig5C P-AMPK 60KD

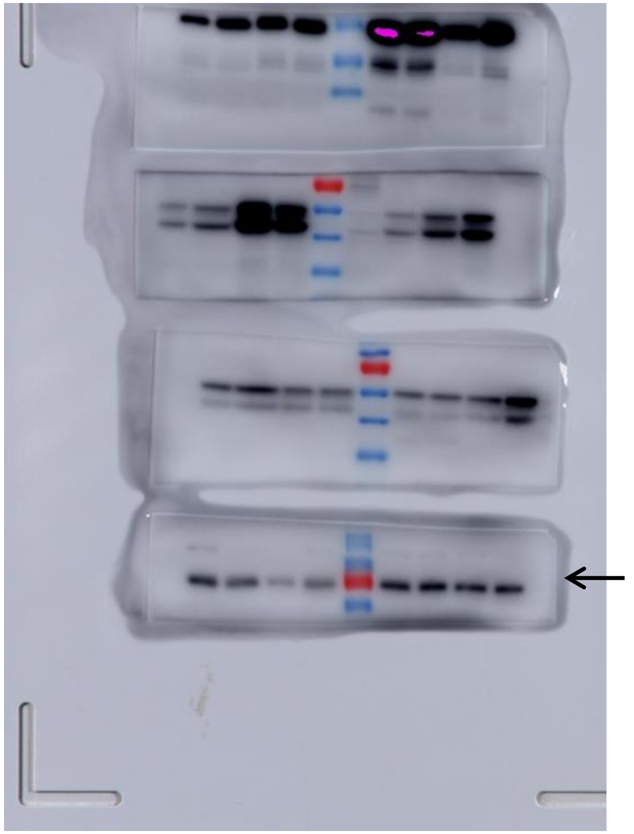

Fig5C AMPK 60KD

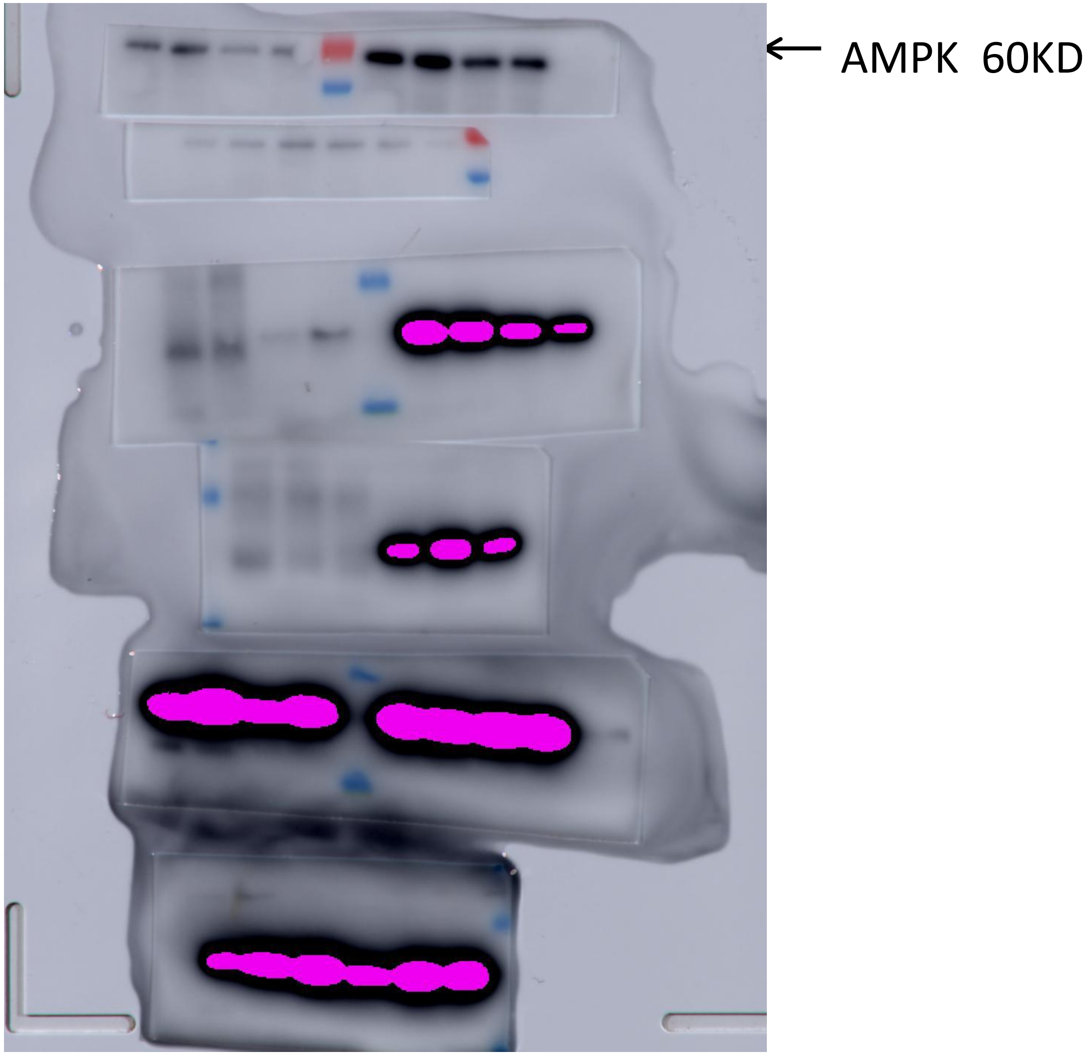

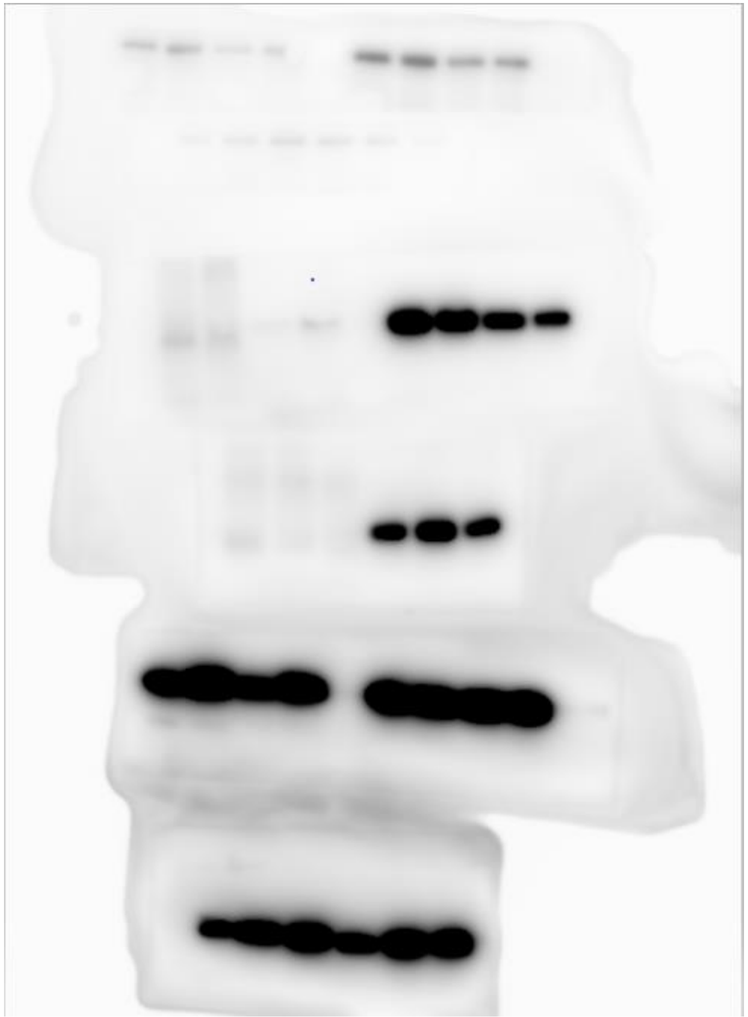

Fig5C P-AKT 473

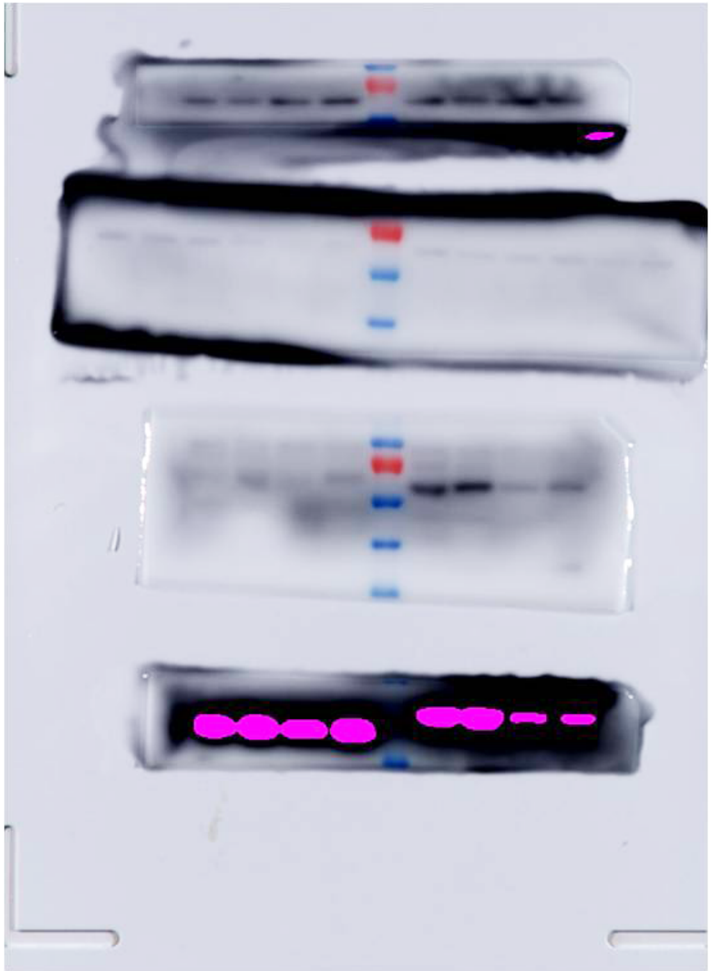

← P-AKT 473

Fig5C P-AKT 308 60KD

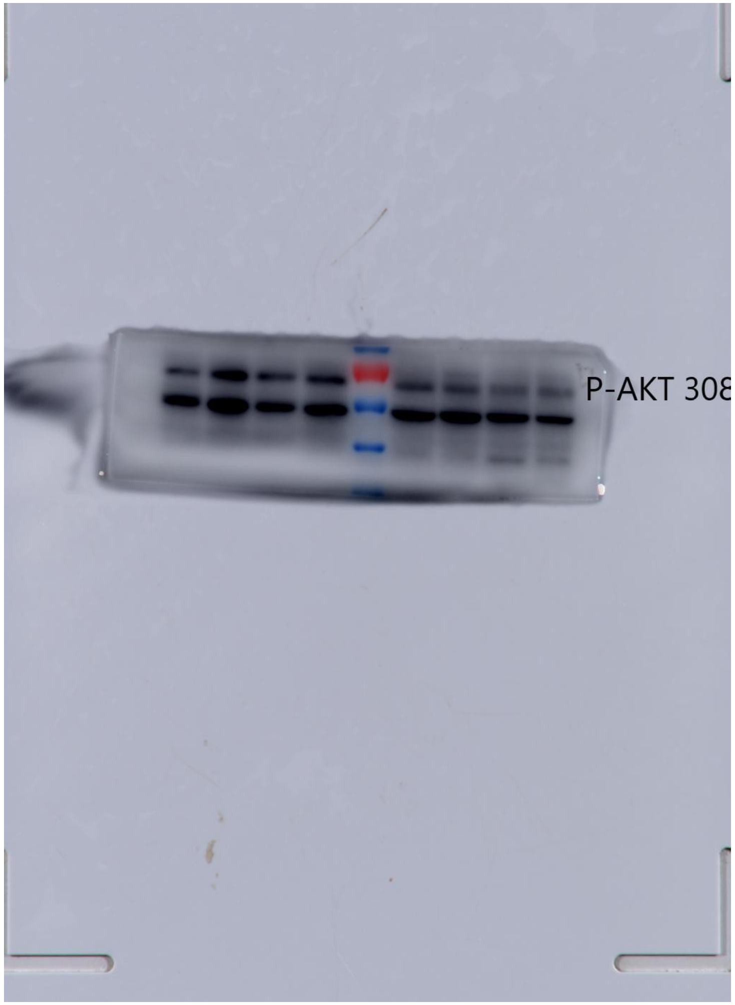

Fig5C AKT 60KD

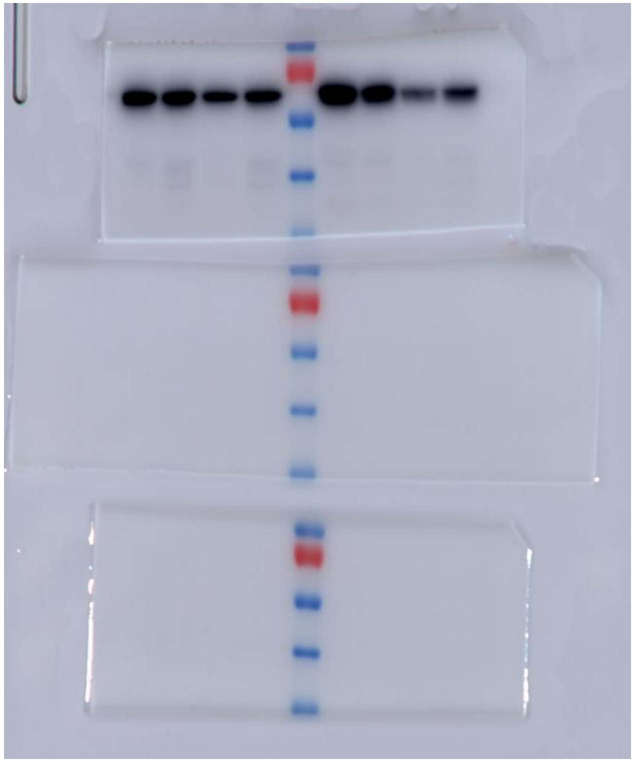

← AKT 60KD

Fig5C mTOR 289KD

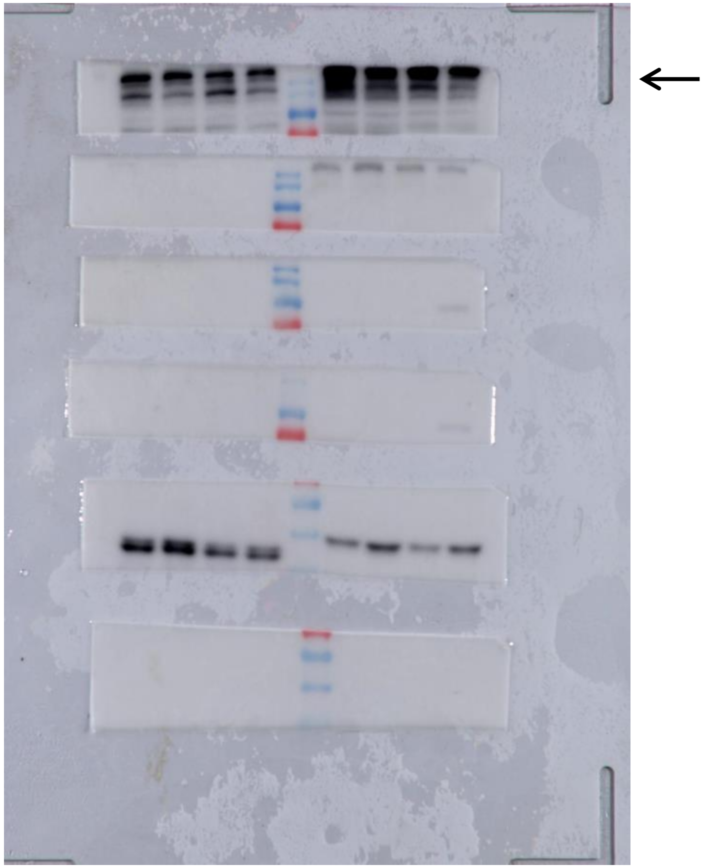

Fig5C P-mTOR2448 289KD

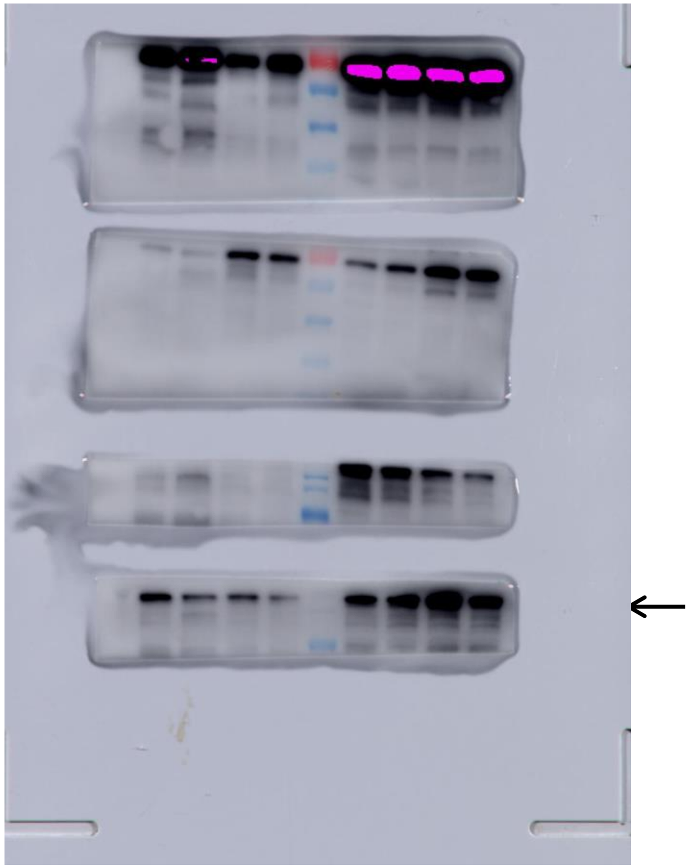

← P-mTOR2448

Fig5C P-mTOR2481

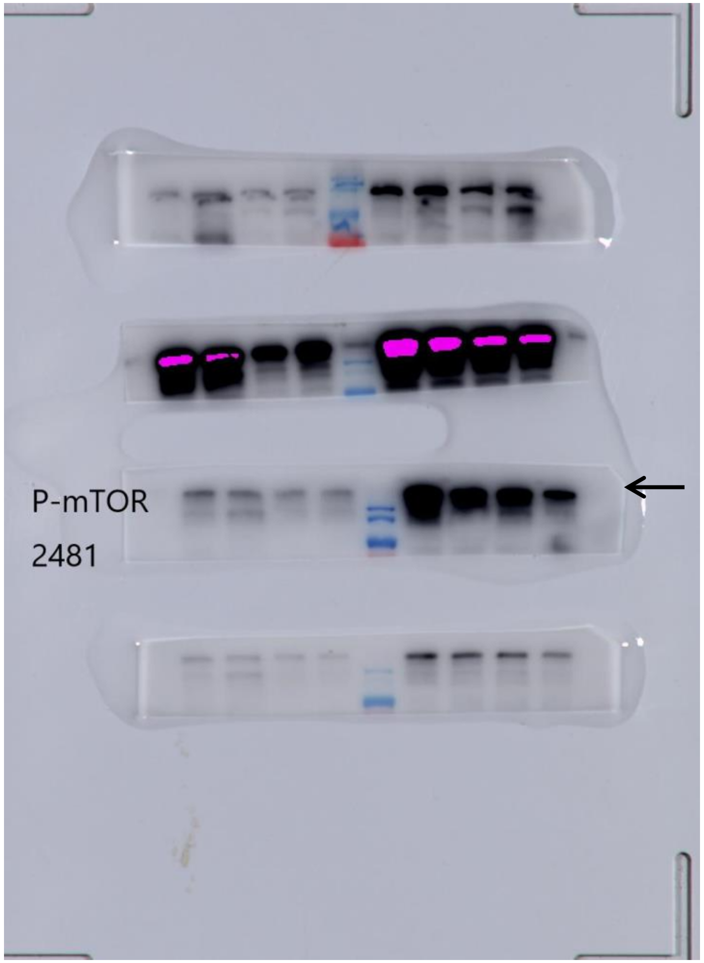

Fig5C P-P70S6K 70KD

P-P70S6K
70KD

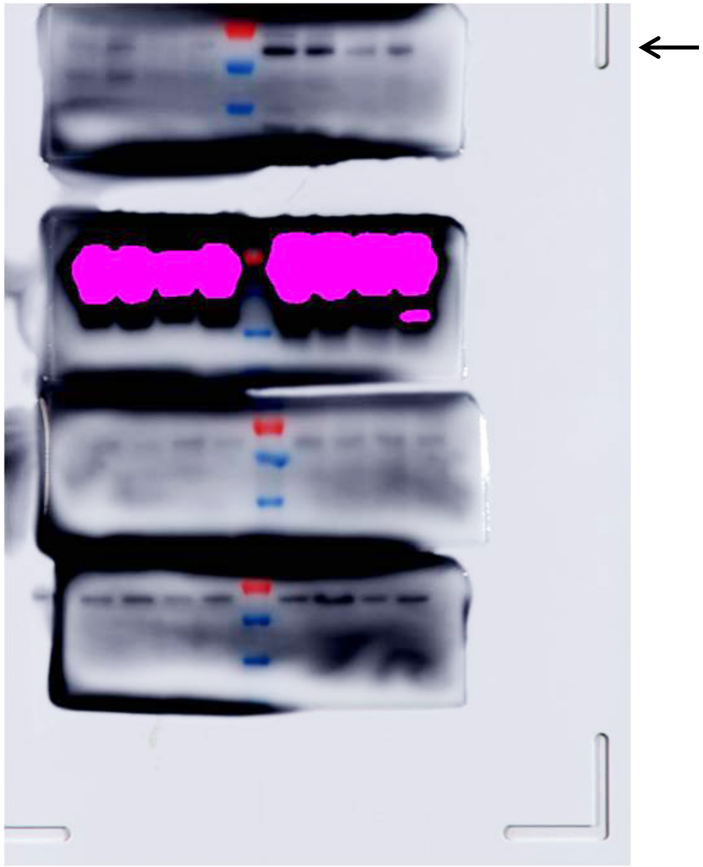

Fig5C P70S6K

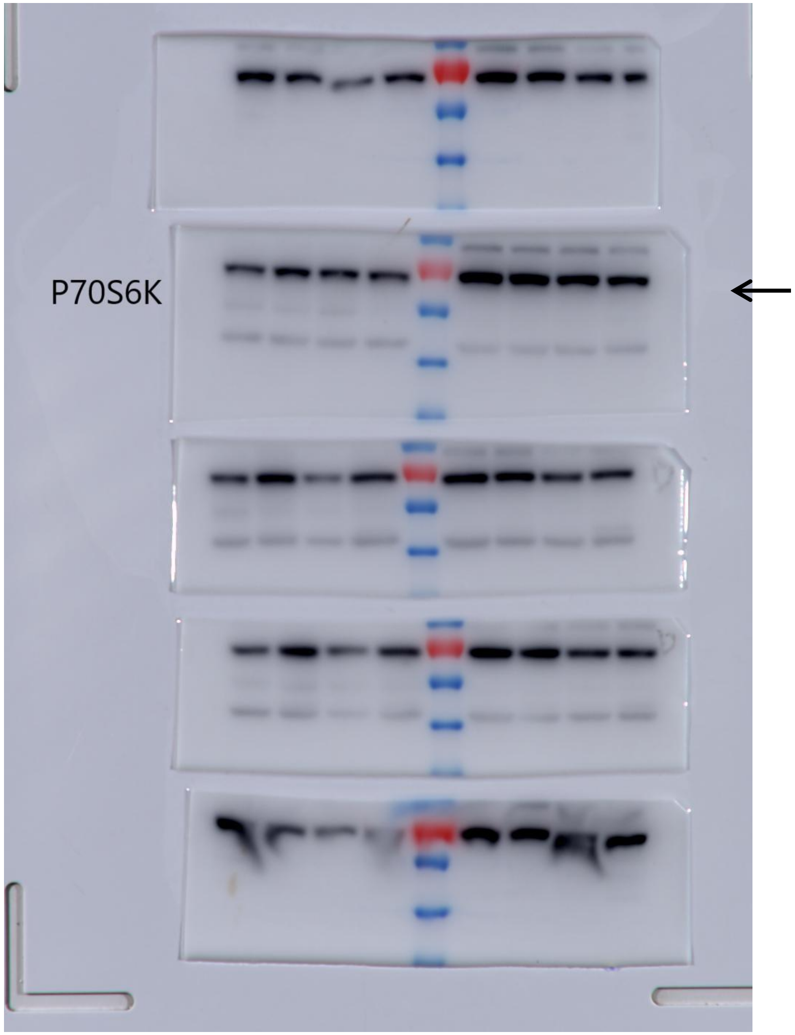

Fig5C Actin 45KD

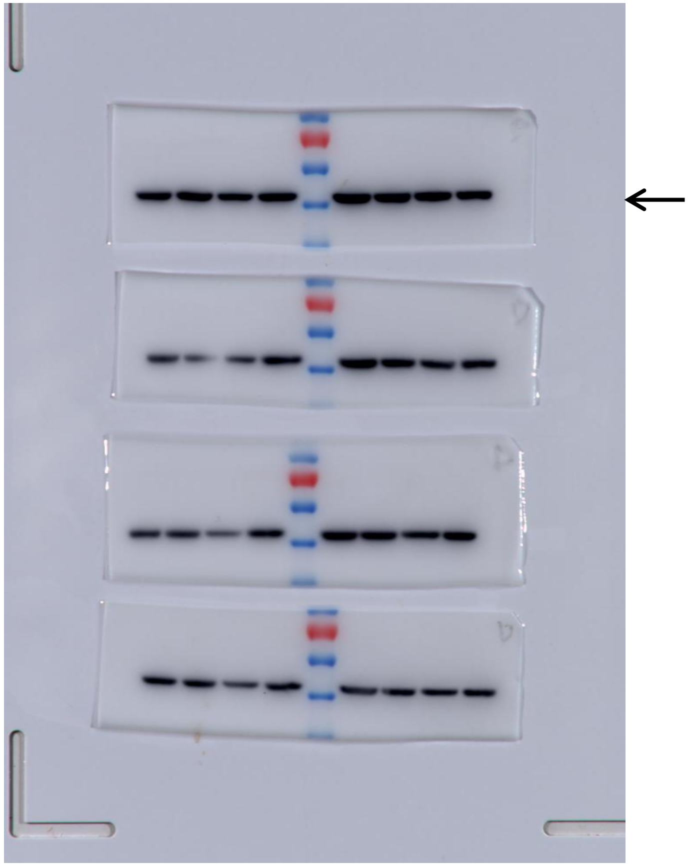

Fig5C p-ERK

p-ERK

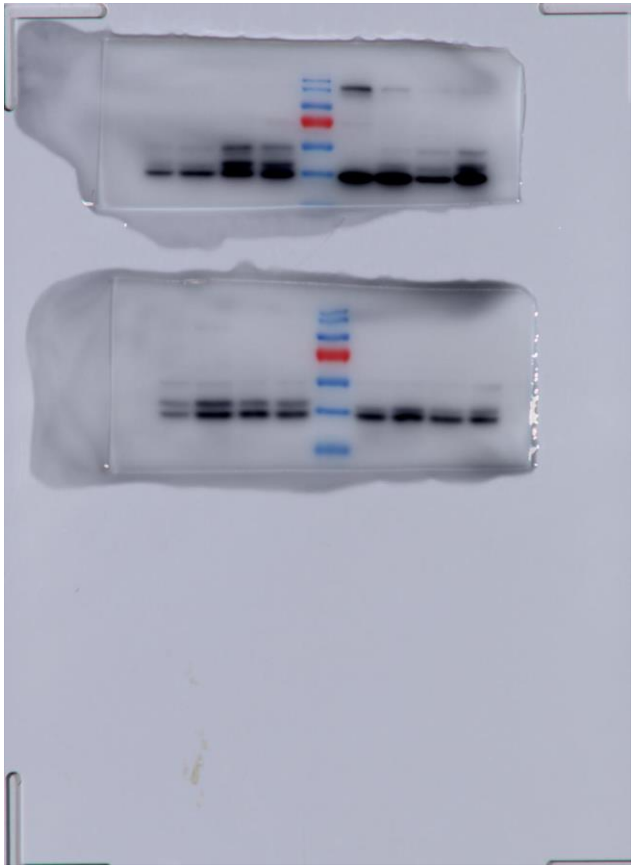

←

Fig5C P-p38

P-p38

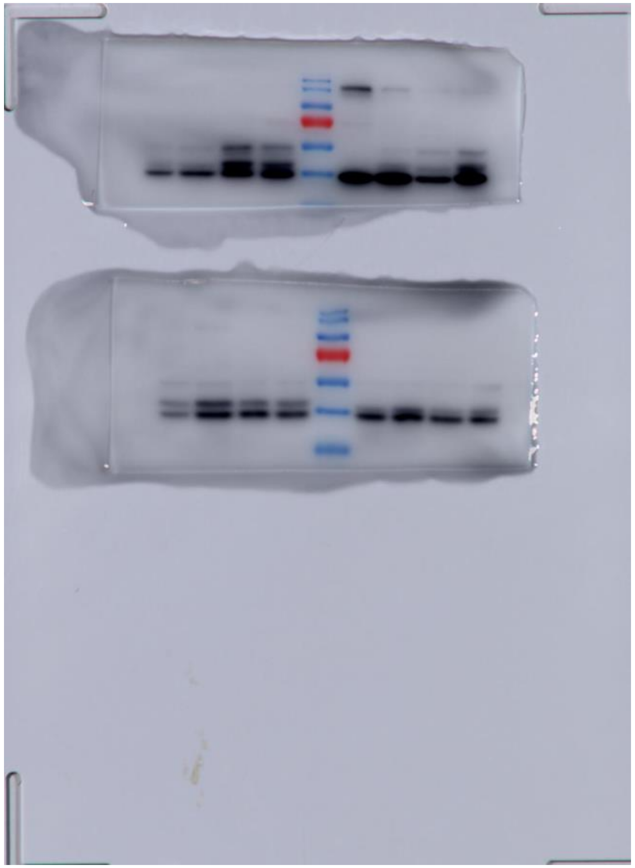

←

Fig5C P-JNK

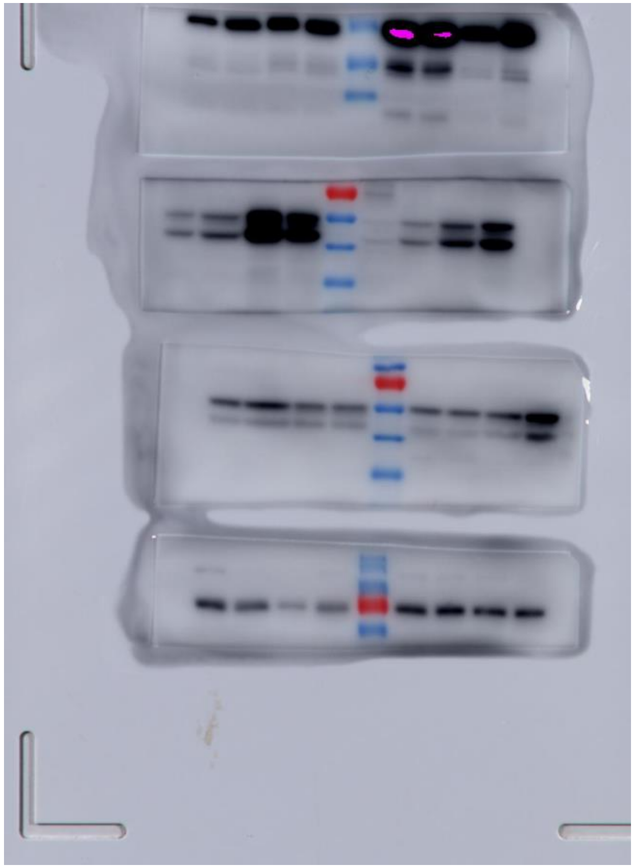

← P-JNK

Fig5C JNK 54,46KD

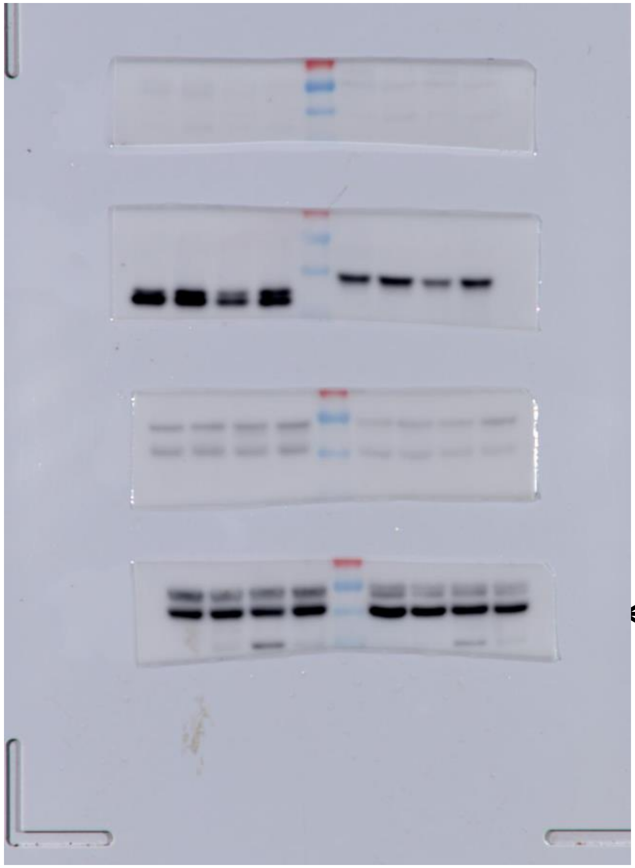

← JNK 54,46KD

Fig5C Actin 45KD

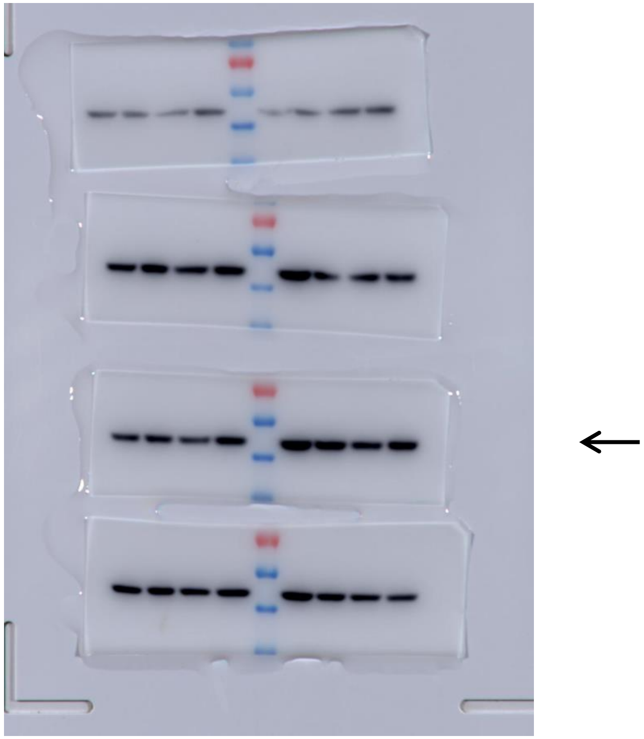

Fig6C Mcl-1 48KD

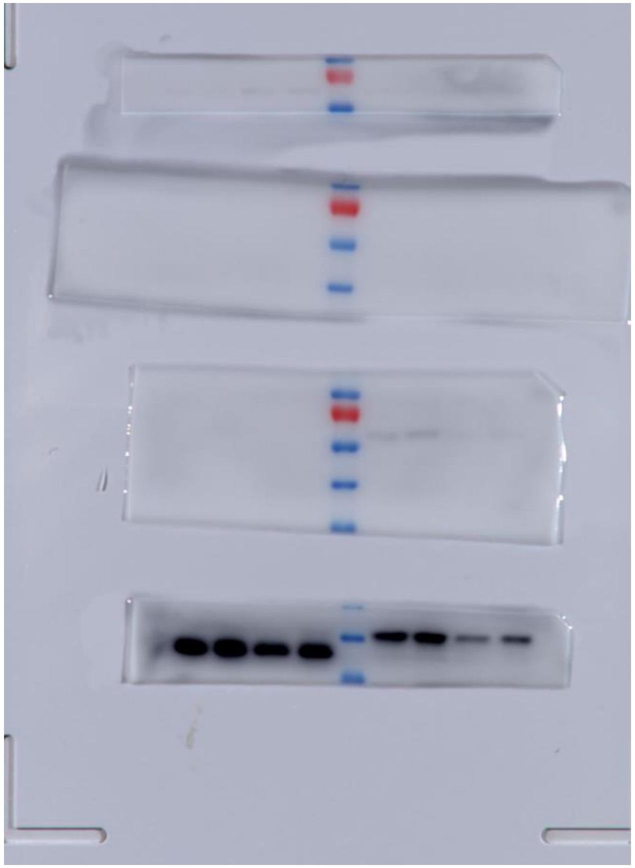

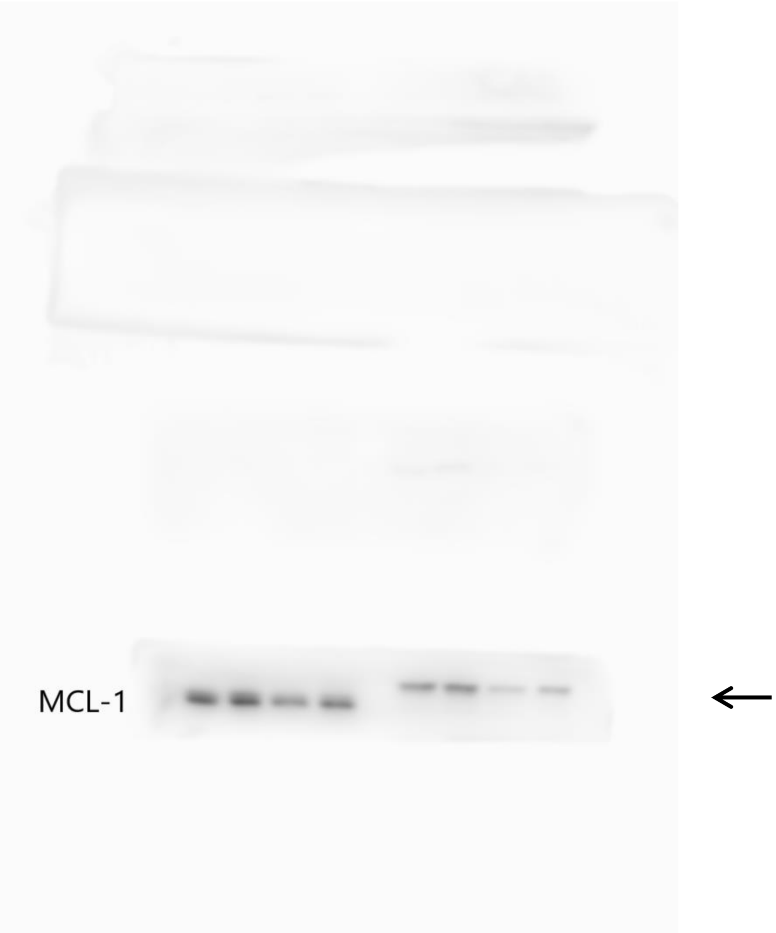

Fig6C Bcl-XL 30KD

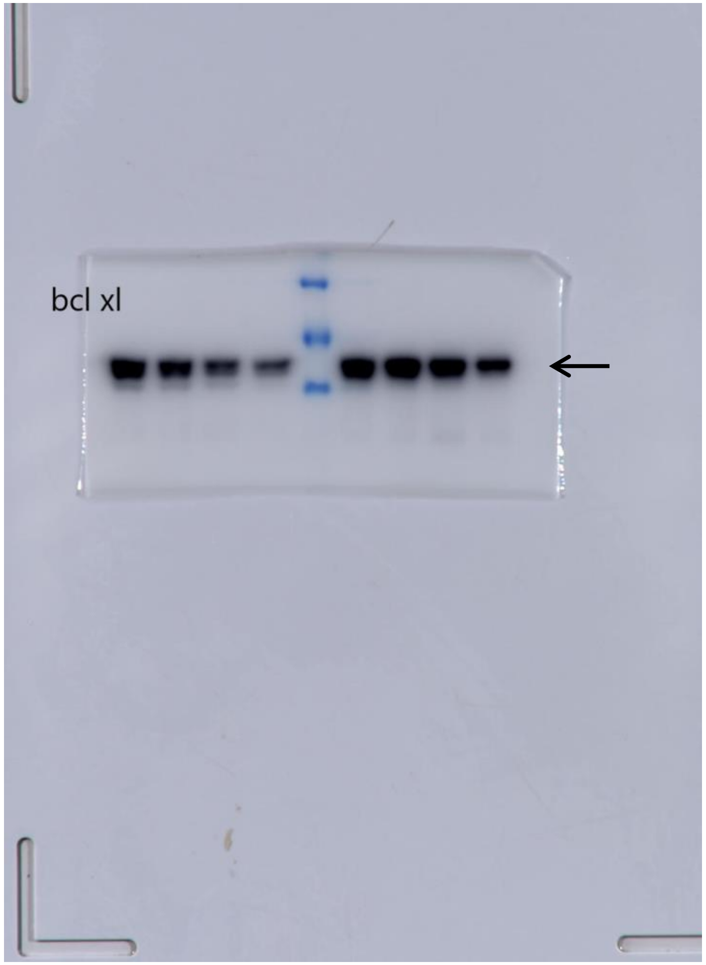

Fig6C Bcl2

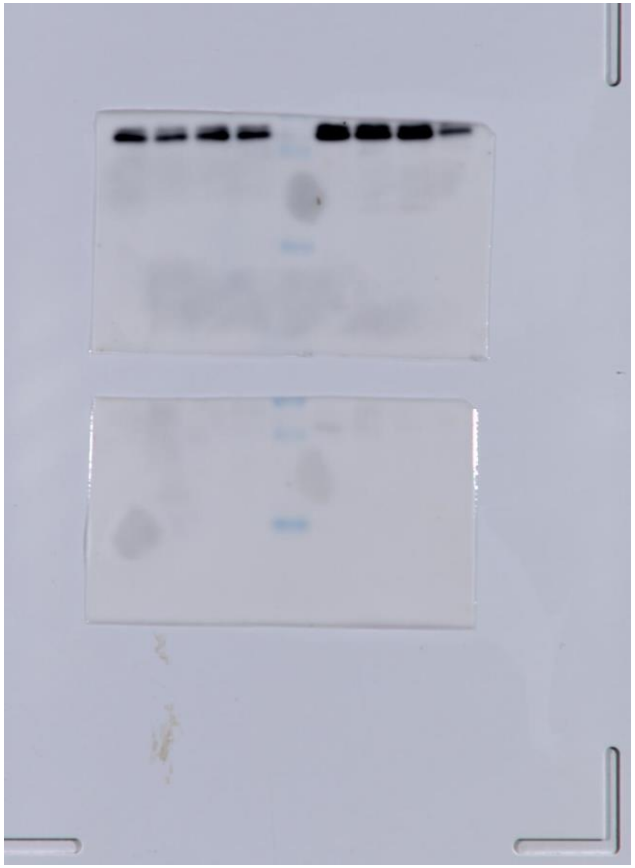

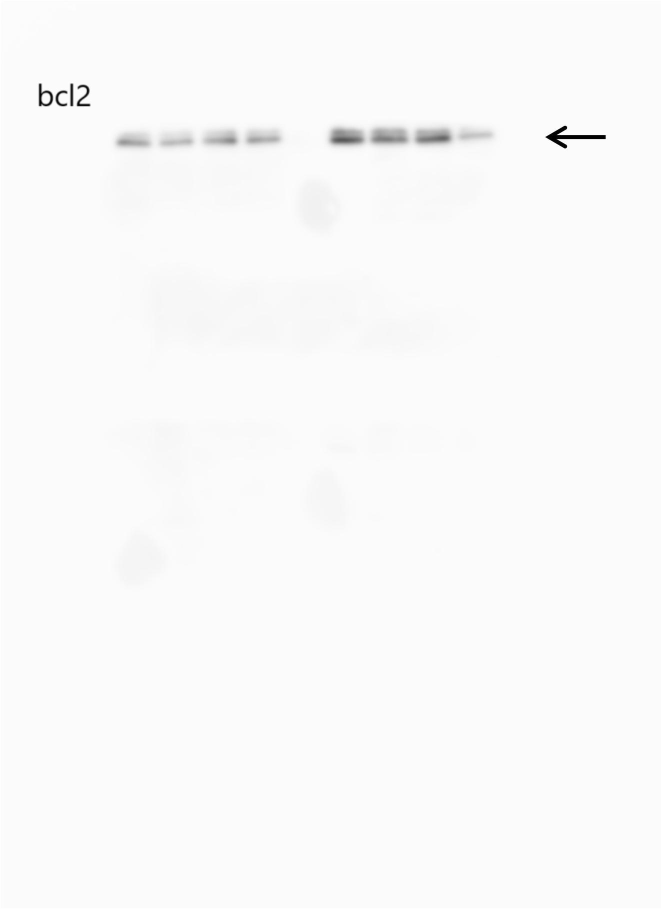

Fig6C Actin 45KD

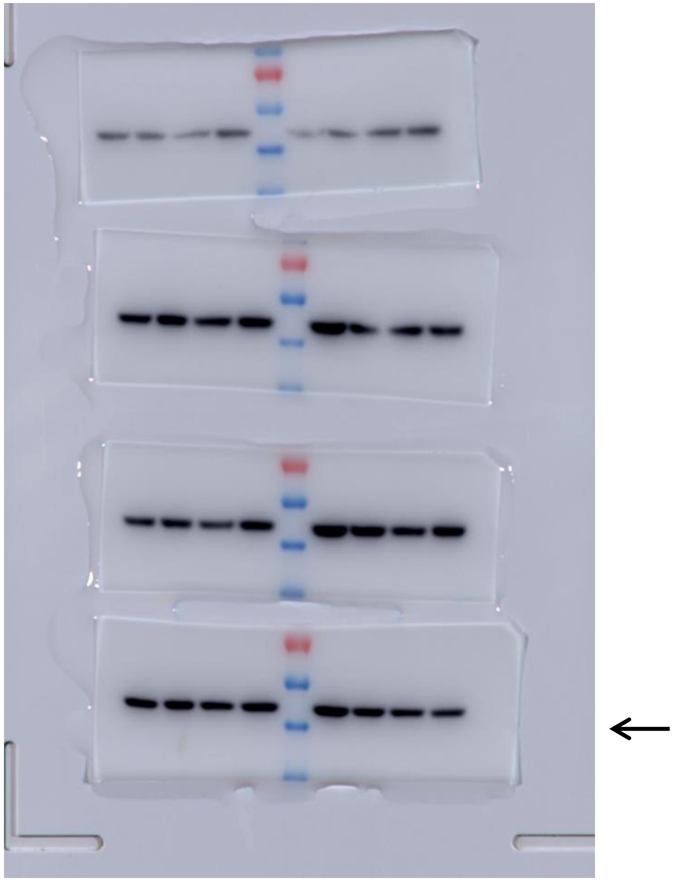

Fig6C c-IAP1 62 KD

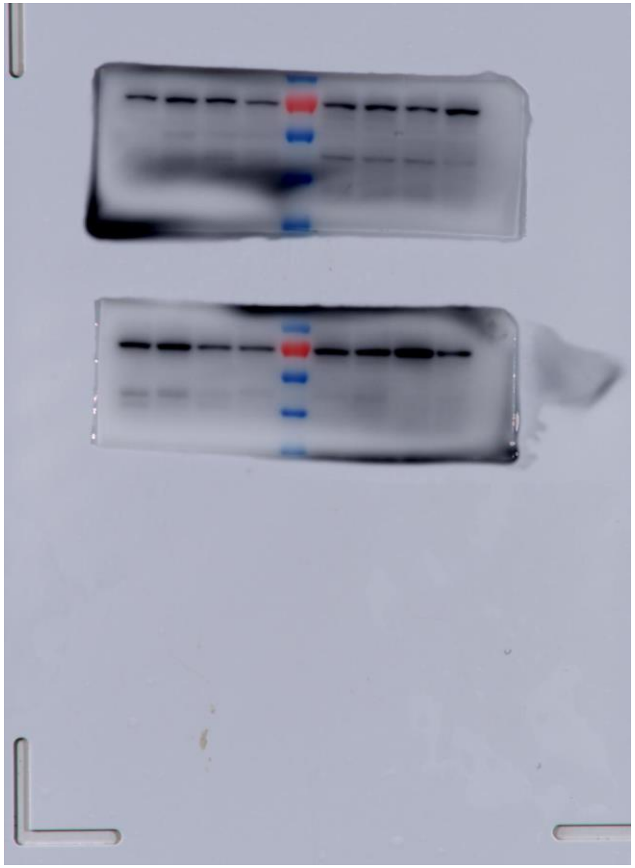

← c-IAP1 62 KD

Fig6C c-IAP2 70KD

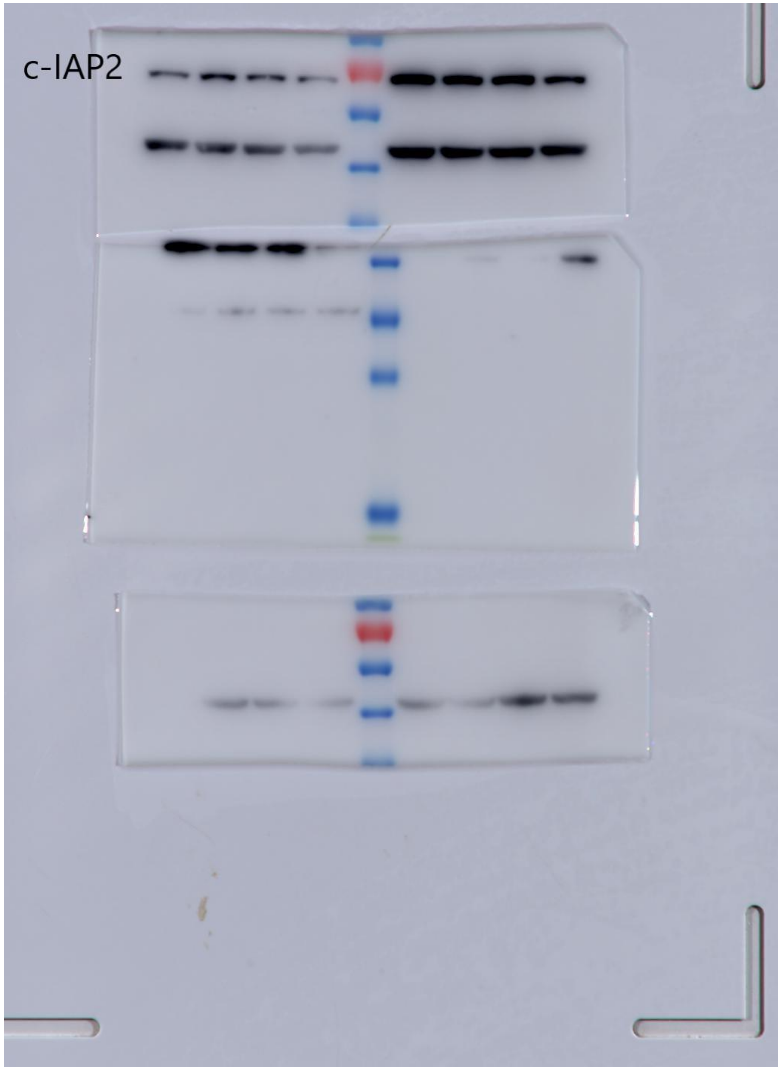

← c-IAP2 70KD

Fig6C XIAP 53KD

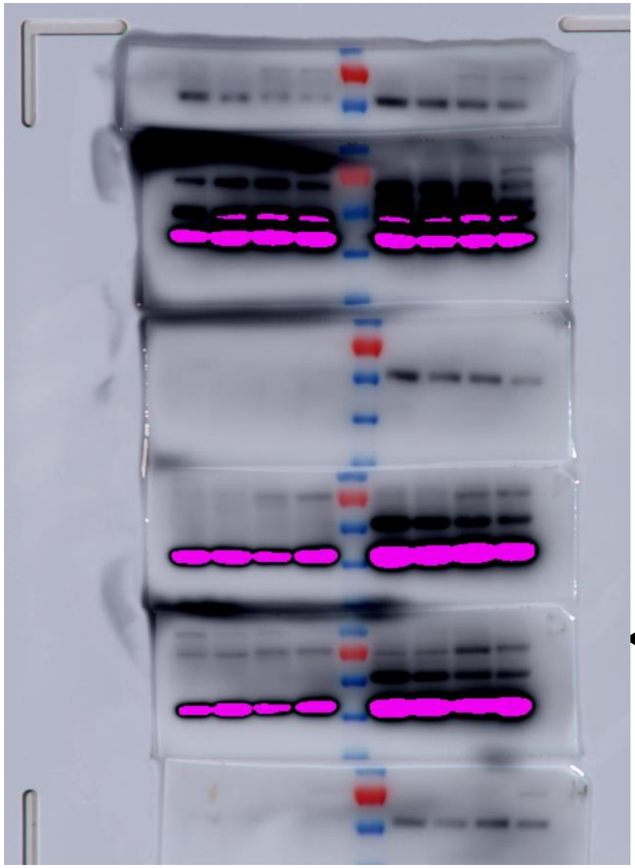

← XIAP 53KD

Fig6C Actin 45KD

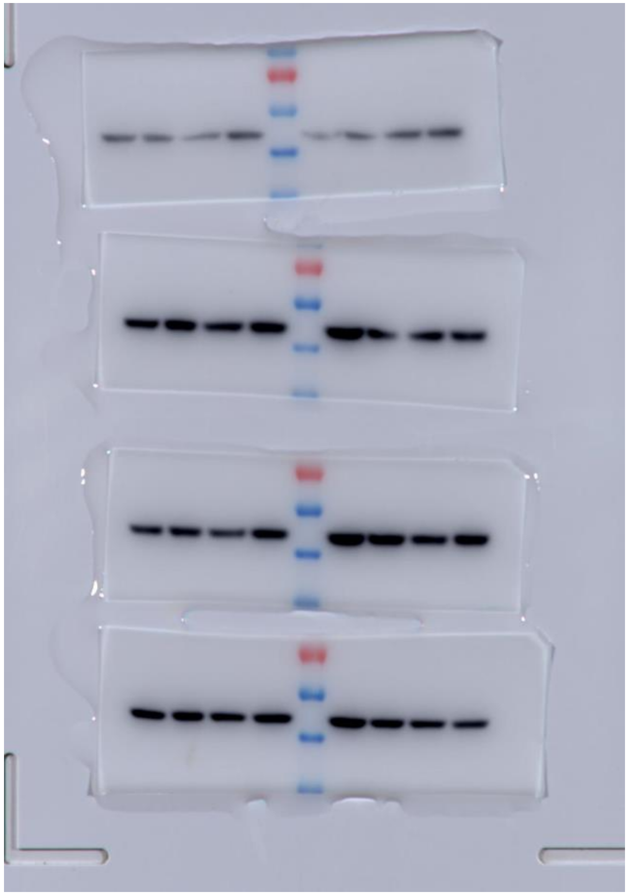

←

Supplementary 4C Bim 23, 12 KD

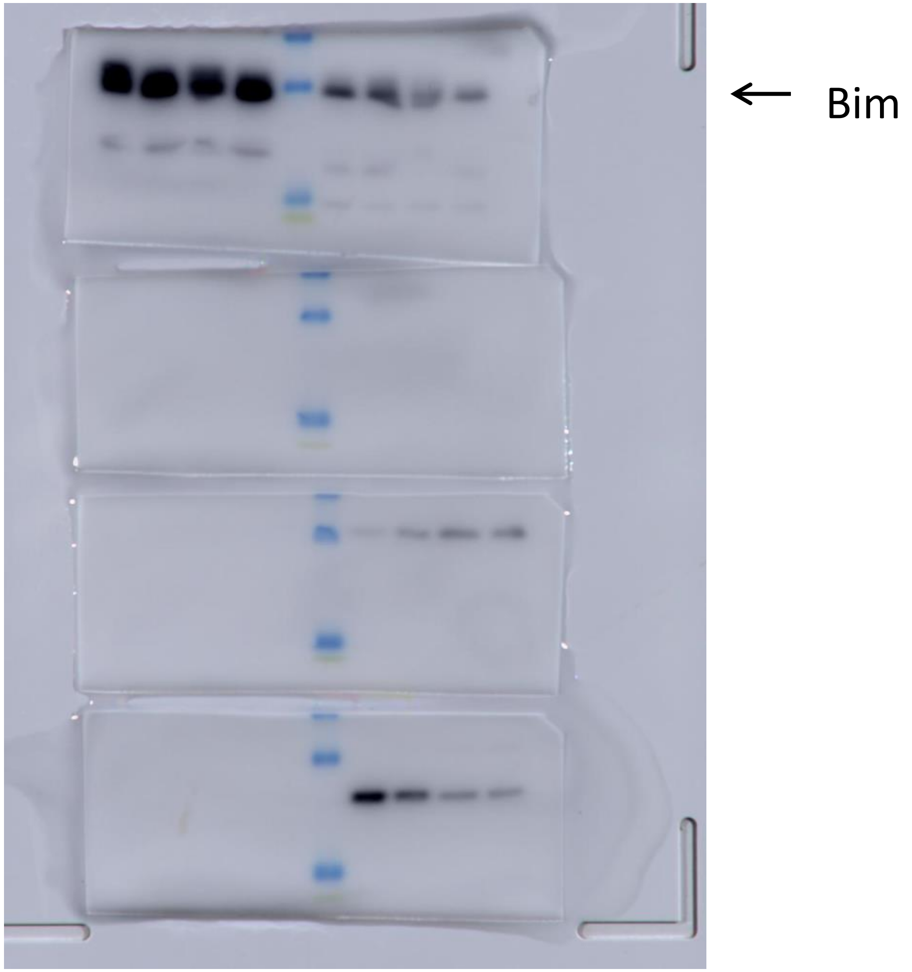

Supplementary 4C BAX20KD

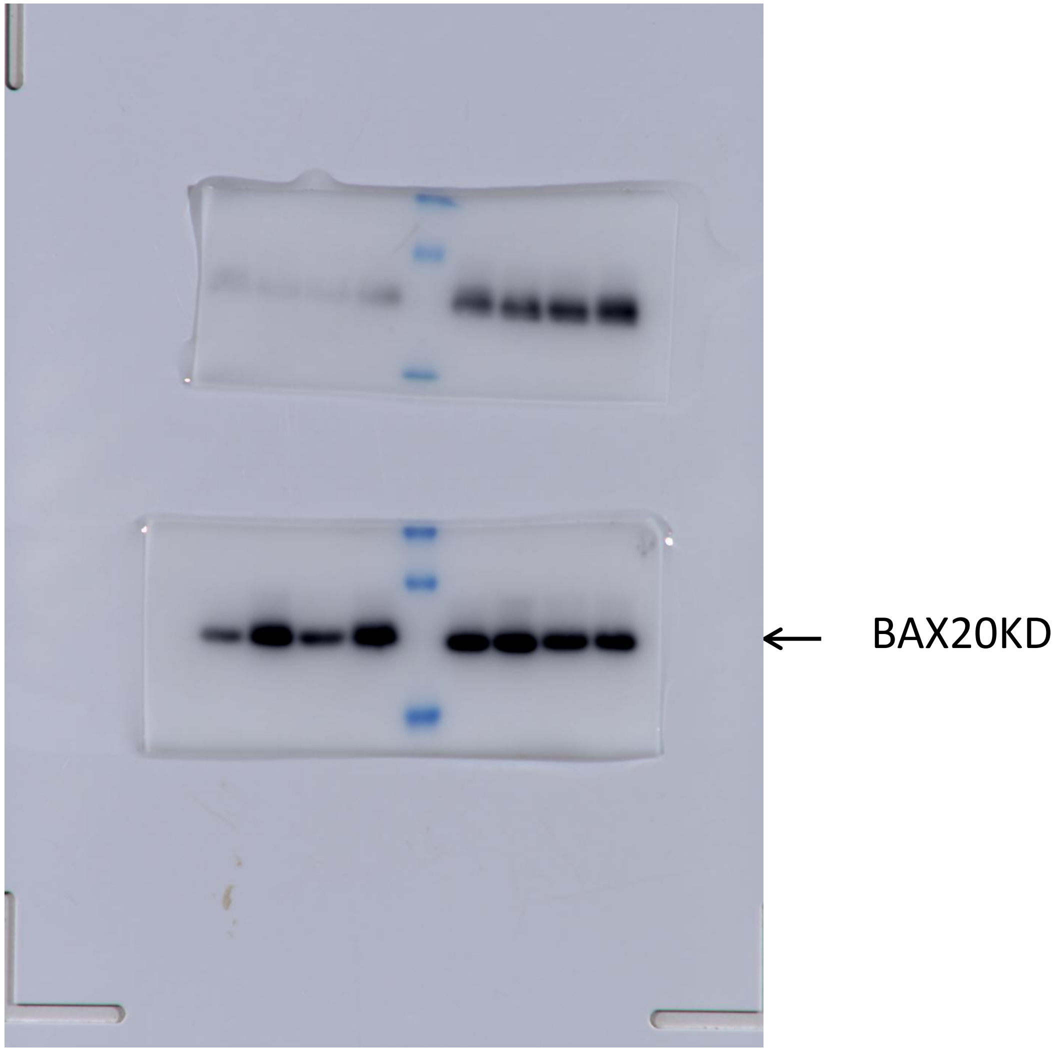

← BAX20KD

Supplementary 4C BAK 25KD

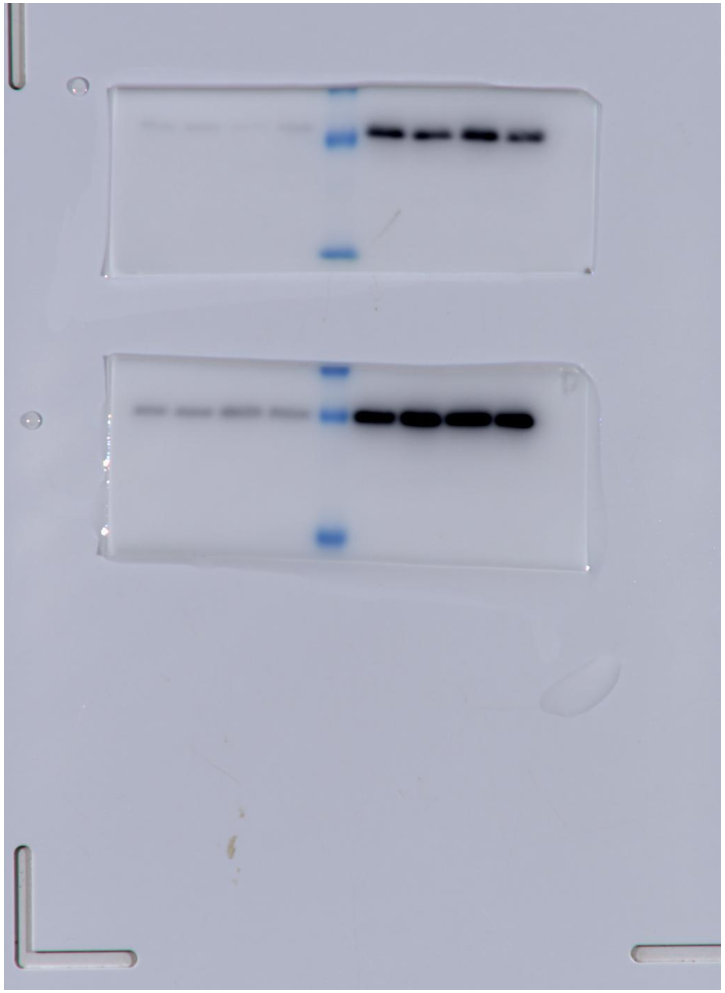

← BAK 25KD

Supplementary 4C Bid 22KD

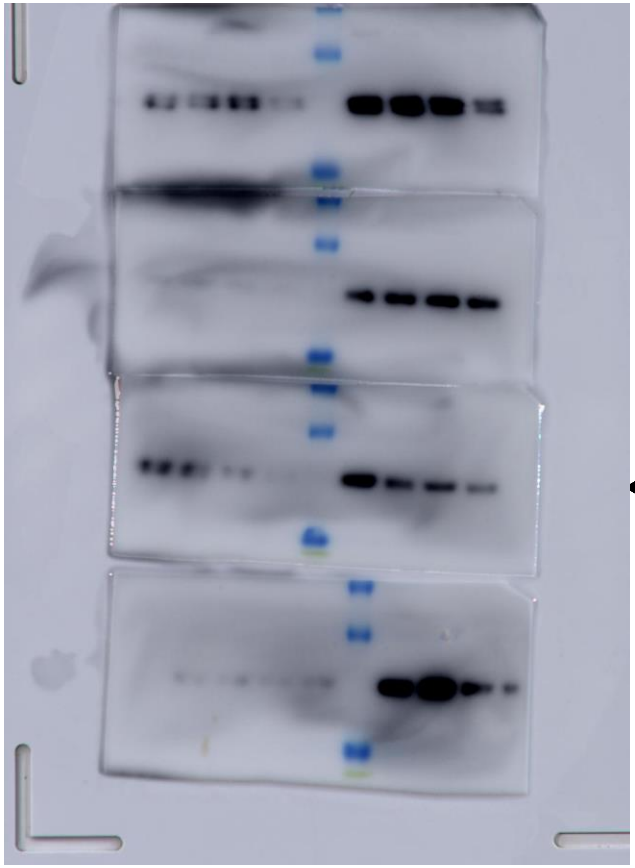

← Bid 22KD

Supplementary 4C Bad23KD

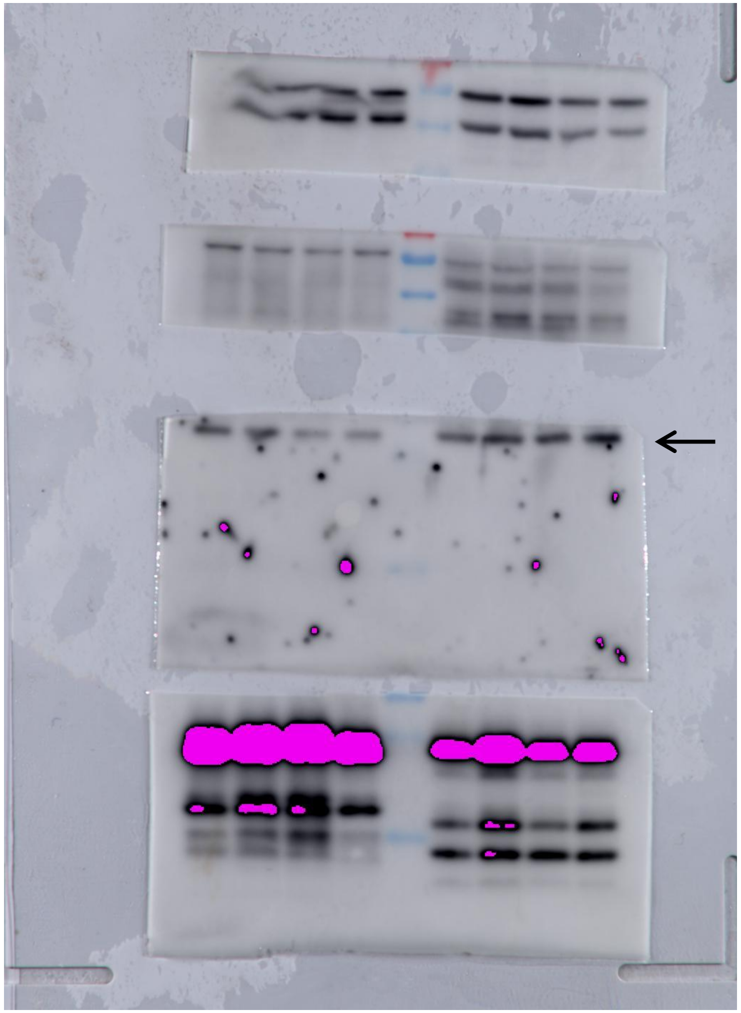

Bad23KD

Supplementary 4C Actin 45KD

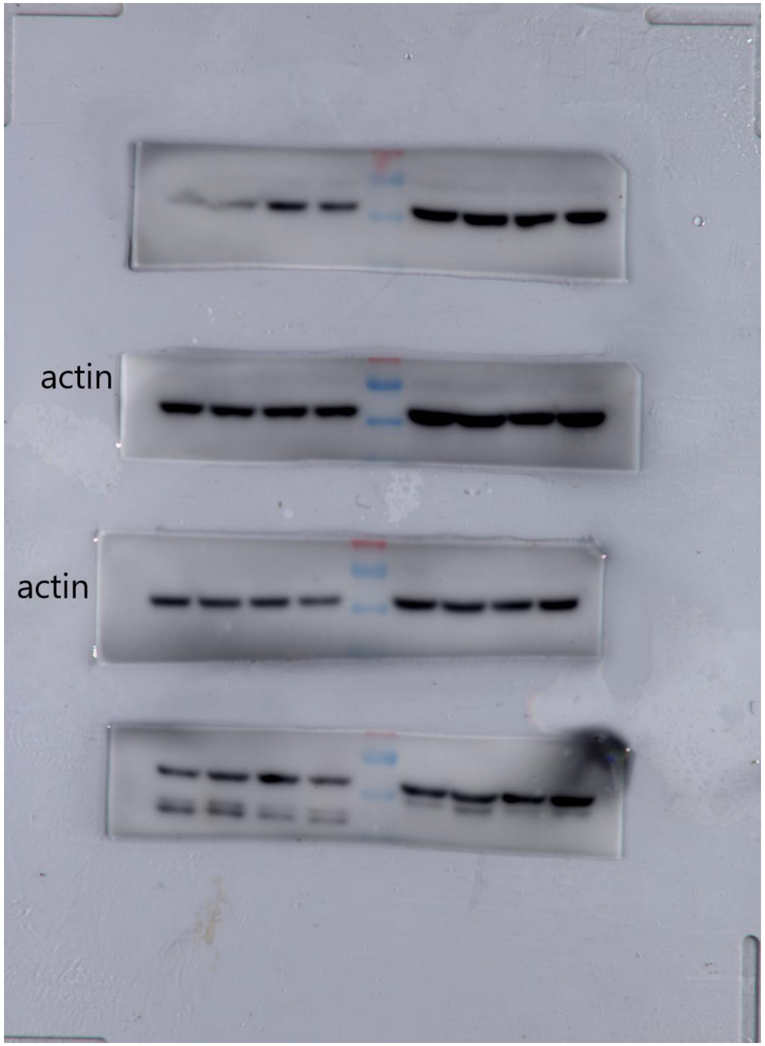

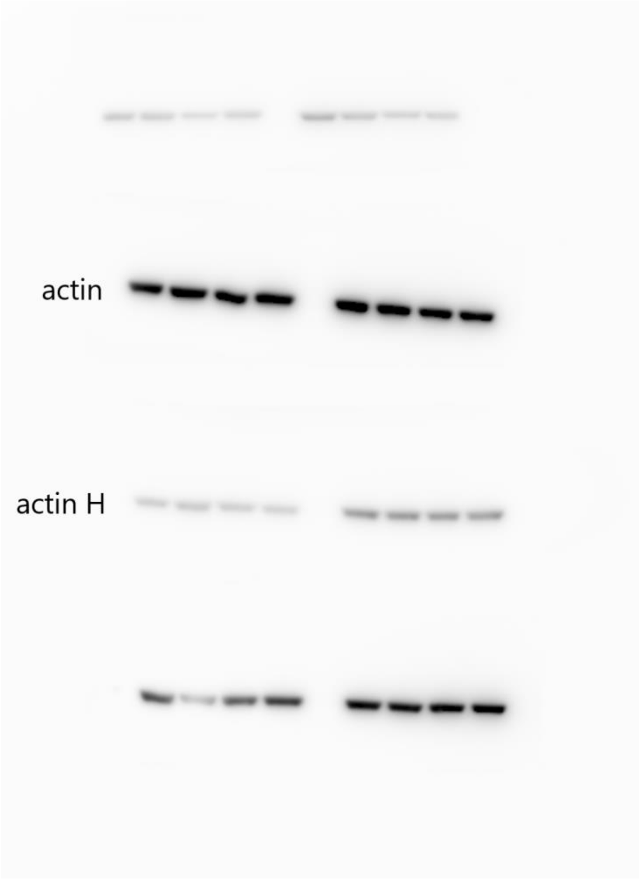

Supplementary6

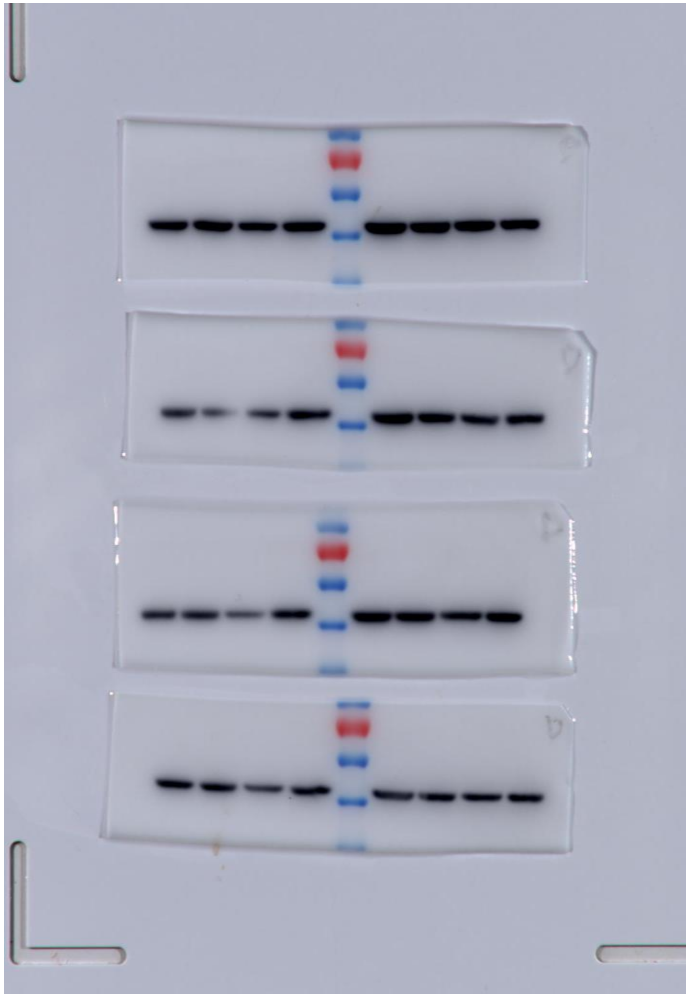

←

Supplementary6 CYPA 17KD

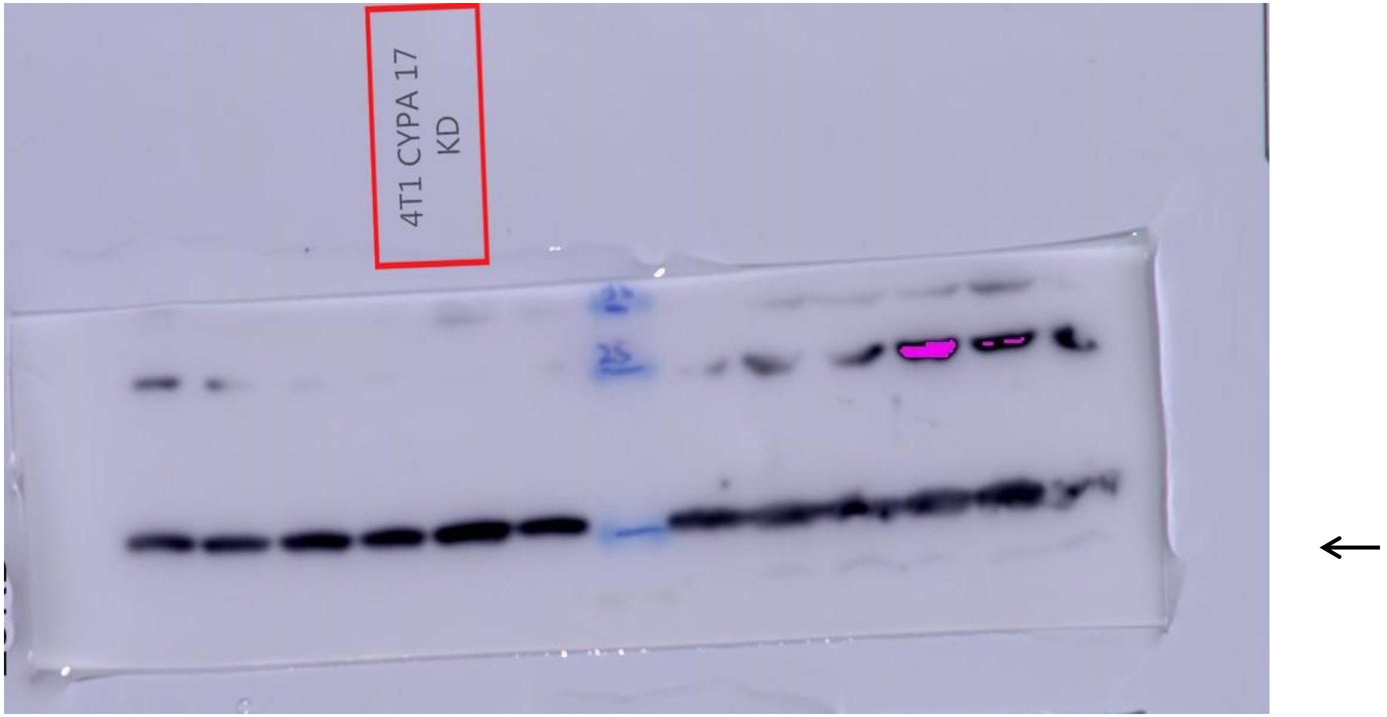

Supplementary6 CYPA, HMGB1

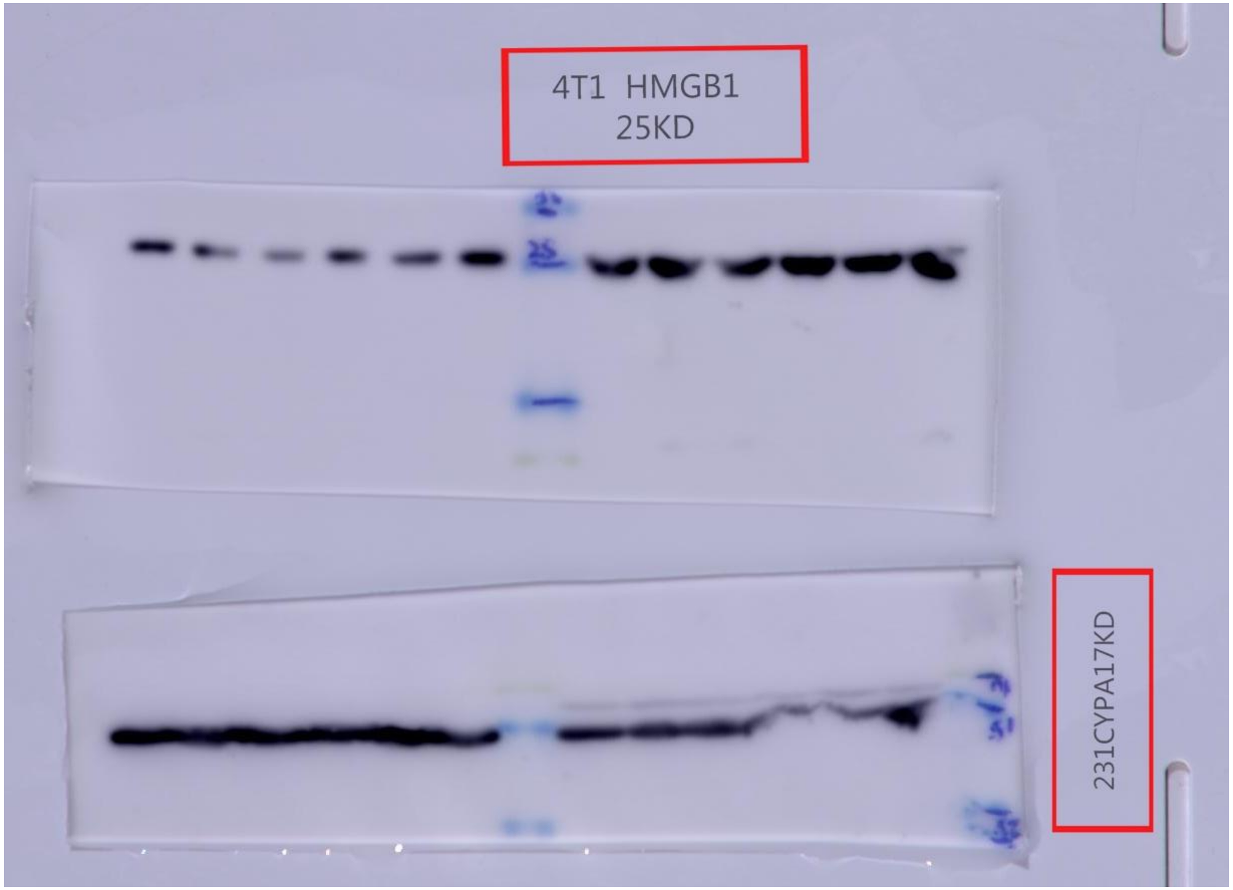

Supplementary6 HMGB1

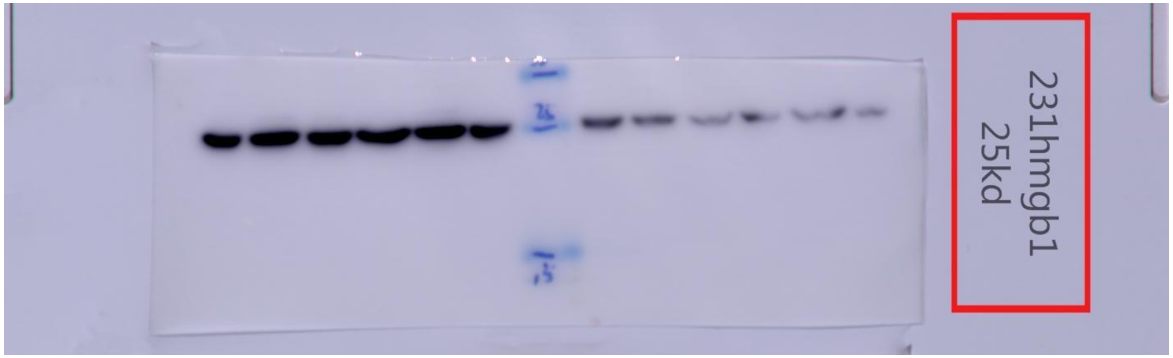

Supplementary6 Actin   45KD

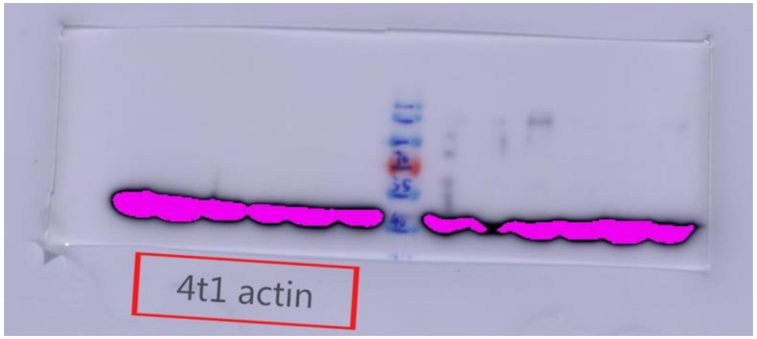

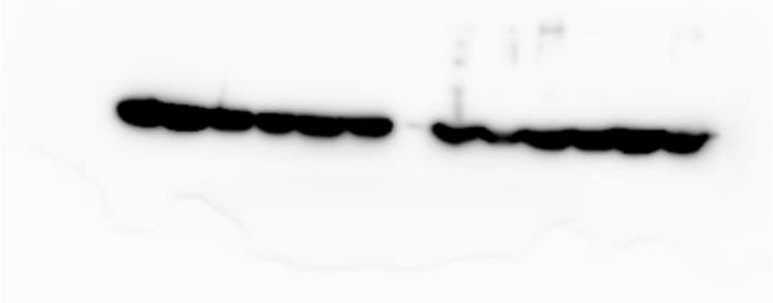

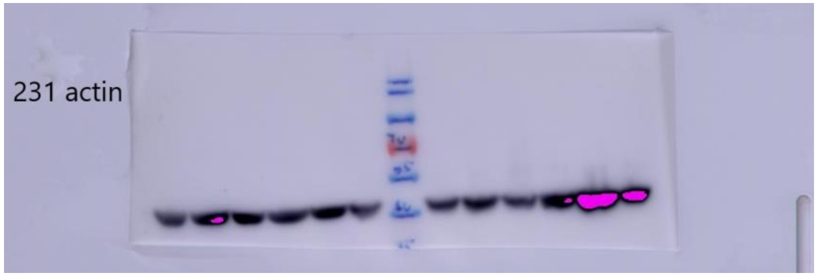

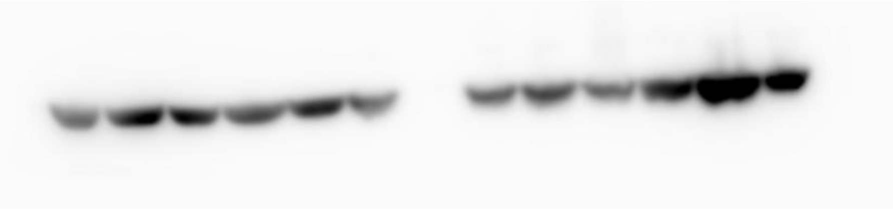

Supplementary7 GSDMD

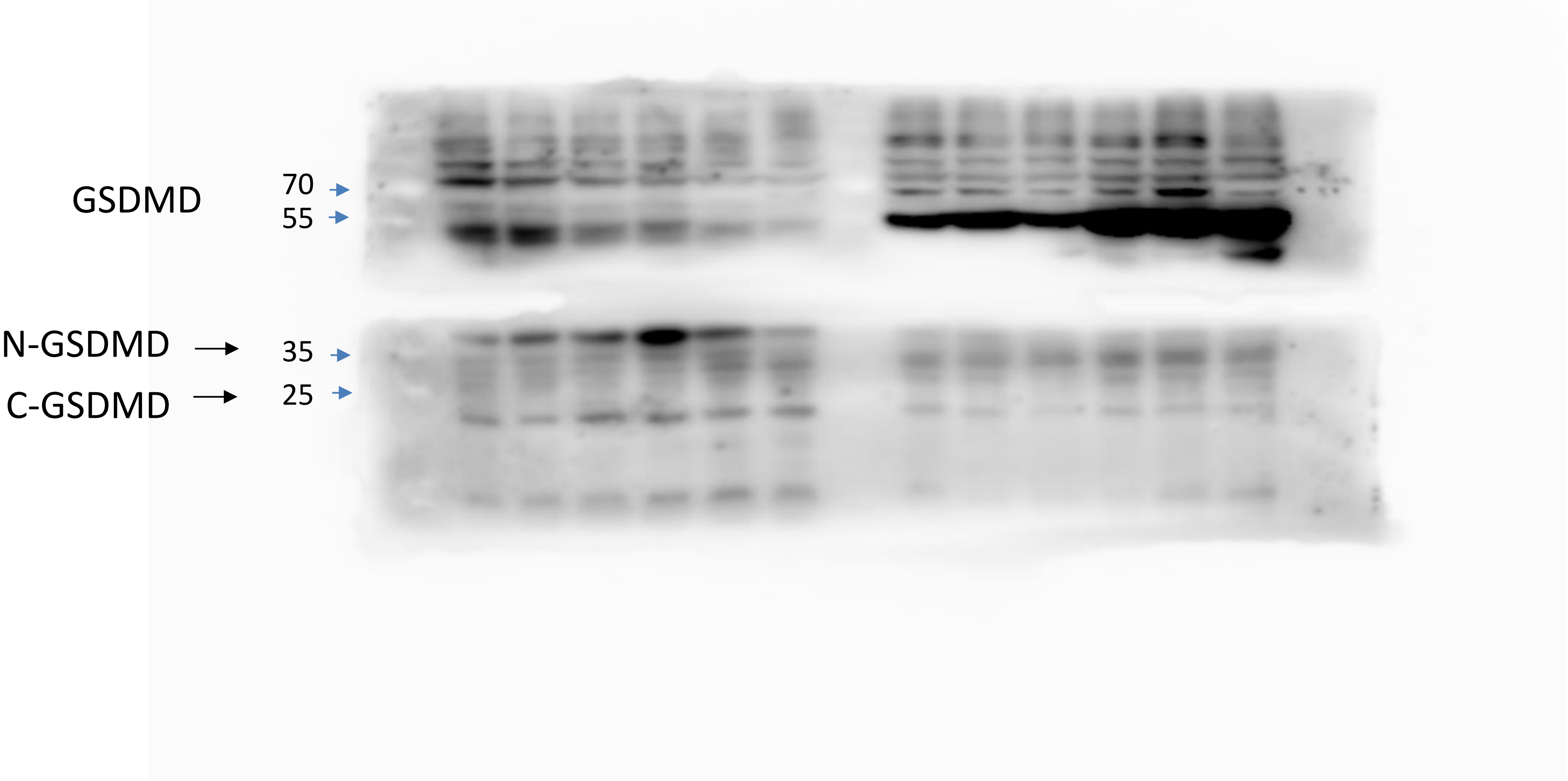

Supplementary7 Actin 45KD

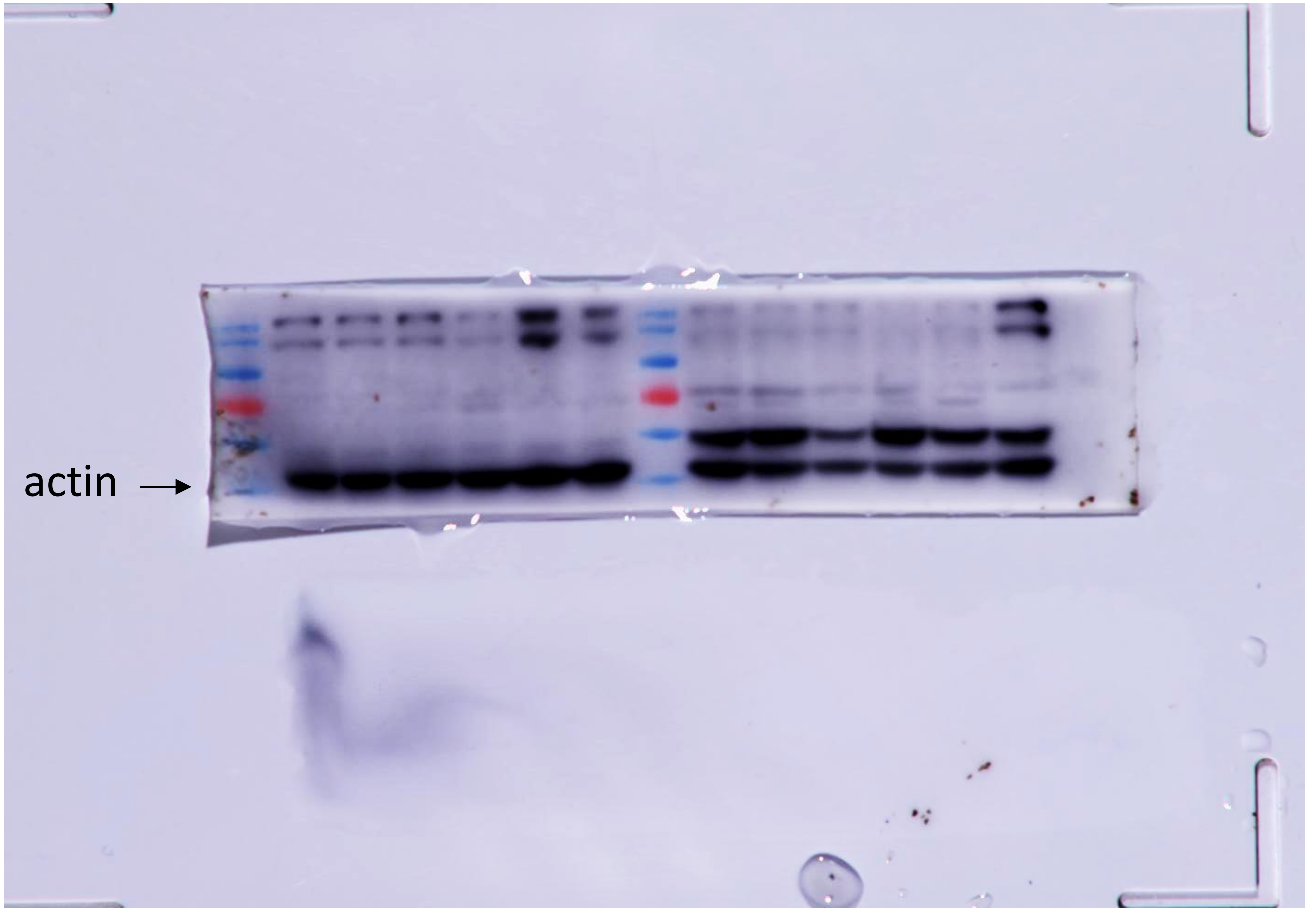

Supplement: Supplementary Files [file BSR-2019-0878_supp1.pdf]
